# Genomic epidemiology of a national outbreak of post-surgical *Mycobacterium abscessus* wound infections in Brazil

**DOI:** 10.1099/mgen.0.000111

**Published:** 2017-05-03

**Authors:** Izzy Everall, Christiane Lourenço Nogueira, Josephine M Bryant, Leonor Sánchez-Busó, Erica Chimara, Rafael da Silva Duarte, Jesus Pais Ramos, Karla Valéria Batista Lima, Maria Luíza Lopes, Moises Palaci, Andre Kipnis, Fernanda Monego, R. Andres Floto, Julian Parkhill, Sylvia Cardoso Leão, Simon R Harris

**Affiliations:** ^1^​ Wellcome Trust Sanger Institute, Hinxton, Cambridge, UK; ^2^​ Departamento de Microbiologia, Imunologia e Parasitologia, Escola Paulista de Medicina, Universidade Federal de São Paulo, Brazil; ^3^​ Molecular Immunity Unit, University of Cambridge Department of Medicine, MRC Laboratory of Molecular Biology, Cambridge, UK; ^4^​ Núcleo de Tuberculose e Micobacterioses, Instituto Adolfo Lutz Av. Dr. Arnaldo, 666 9o andar São Paulo, SP, Brazil; ^5^​ Instituto de Microbiologia Professor Paulo de Góes, Universidade Federal do Rio de Janeiro, Brazil; ^6^​ Centro de Referência Professor Helio Fraga, Fiocruz, Brazil; ^7^​ Bacteriology and Mycology Section, Instituto Evandro Chagas, Para, Brazil; ^8^​ Nucleo de Doencas Infecciosas, Universidade Federal do Espirito Santo, Brazil; ^9^​ Departamento de Microbiologia, Universidade Federal de Goiás, Brazil; ^10^​ Departamento de Medicina Veterinária, Universidade Federal do Paraná, Brazil

**Keywords:** *Mycobacterium abscessus*, transmission, nosocomial infections, outbreak, Genomic epidemiology

## Abstract

An epidemic of post-surgical wound infections, caused by a non-tuberculous mycobacterium, has been on-going in Brazil. It has been unclear whether one or multiple lineages are responsible and whether their wide geographical distribution across Brazil is due to spread from a single point source or is the result of human-mediated transmission. 188 isolates, collected from nine Brazilian states, were whole genome sequenced and analysed using phylogenetic and comparative genomic approaches. The isolates from Brazil formed a single clade, which was estimated to have emerged in 2003. We observed temporal and geographic structure within the lineage that enabled us to infer the movement of sub-lineages across Brazil. The genome size of the Brazilian lineage was reduced relative to most strains in the three subspecies of *Mycobacterium abscessus* and contained a novel plasmid, pMAB02, in addition to the previously described pMAB01 plasmid. One lineage, which emerged just prior to the initial outbreak, is responsible for the epidemic of post-surgical wound infections in Brazil. Phylogenetic analysis indicates that multiple transmission events led to its spread. The presence of a novel plasmid and the reduced genome size suggest that the lineage has undergone adaptation to the surgical niche.

## Abbreviations

AAI, average amino acid identity; ANI, average nucleotide identity; CDS, coding sequence; DCC, dominant circulating clone; MABSC, Mycobacterium abscessus species complex; MST, Minimum Spanning Tree; T7SS, type VII secretion system.

## Data Summary

1. All Genomes sequenced in this study have been deposited in the European Nucleotide Archive; accession number ERP006740 (http://www.ebi.ac.uk/ena/data/view/PRJEB7058).

2. The reference selected from the newly sequenced isolates and its annotation can accessed through European Nucleotide Archive accession number GCA_900169205; chromosome accession FWDG01000001; plasmid accession FWDG01000002 (http://www.ebi.ac.uk/ena/data/view/GCA_900169205.1).

3. This research was supplemented by sequences from a global population study available from the European Nucleotide Archive; accession number ERP001039 (http://www.ebi.ac.uk/ena/data/view/PRJEB2779).

Genome sequence GO 06 is available from GenBank; accession number CP003699.2 (https://www.ncbi.nlm.nih.gov/nuccore/CP003699.2).

Genome sequence CRM-0020 is available from GenBank; accession number ATFQ00000000.1 (https://www.ncbi.nlm.nih.gov/nuccore/ATFQ00000000.1).

Genome Sequence 47J26 is available from GenBank; accession number AGQU00000000.1 (https://www.ncbi.nlm.nih.gov/nuccore/AGQU00000000.1).

The outbreak phylogeny can be viewed using microreact https://microreact.org/project/HksrHGmte.

## Impact Statement

In this work, we used genome data to investigate the epidemic of post-surgical skin and soft tissue infections, caused by *Mycobacterium abscessus* subspecies *massiliense*, in Brazil. It has been unclear whether one or multiple lineages are responsible for the epidemic and how the outbreak has spread throughout Brazil. We show that a single lineage of *M. a. massiliense* is responsible for the epidemic. The geographical clustering of isolates in the phylogeny rules out the possibility of the outbreak lineage being disseminated from a single point source, suggesting that the lineage has been transmitted between outbreak locations. Our study advances the research field by clarifying the causal lineage of this outbreak and highlighting the ability of this pathogen to undergo long distance transmission events and persist in the hospital environment. We also uncovered new evidence that suggests that this lineage has adapted to the surgical environment within Brazil and, with further experimental work, this could reveal novel genes associated with the success of this lineage. More broadly, this study shows how whole genome sequencing can be used for outbreak analysis.

## Introduction

The *Mycobacterium abscessus* species complex (MABSC) consists of three subspecies, *M. abscessus* subspecies *abscessus (M. a. abscessus)*, *M. abscessus* subspecies *bolletii* (*M. a. bolletii*) and *M. abscessus* subspecies *massiliense* (*M. a. massiliense*) [1, 2]. Outbreaks of both skin and soft tissue and pulmonary infections caused by the MABSC are becoming more common, with outbreaks having been reported in the UK, USA, Taiwan and South Korea over the past two decades [3–7]. However, Brazil has seen the largest number, with over 2128 skin and soft tissue infections reported in 23 states since 2004, including large outbreaks in 15 states [8–10]. In contrast, just 13 cases of MABSC were reported in Brazil in the years preceding the epidemic [9].

Molecular typing techniques have suggested that the outbreaks, which occurred in geographically distant locations in Brazil, were caused by a single lineage of *M. a. massiliense*. Isolates sampled from post-surgical wound infections collected during the initial outbreak in Pará (2004–2005) [7] and subsequent outbreaks in Goiás (2005–2007) [11], Rio de Janeiro (2006–2007) [12], Paraná (2007–2008) [13] and Rio Grande do Sul (2007–2011) [14] had identical *rpoB* sequences and pulse field gel electrophoresis (PFGE) patterns, although several isolates were missing a 50 kb band believed to be a recently described plasmid, pMAB01, identified from an isolate belonging to this lineage [7, 9, 11, 15].

The majority of infections occurred after video-assisted surgeries where the equipment had been disinfected using 2 % glutaraldehyde solution. Isolates recovered from these patients were found to be glutaraldehyde-resistant [8]. This, combined with the observation of poor sterilization practices, led to the hypothesis that a lineage of *M. a massiliense* was exposed to non-lethal levels of glutaraldehyde resulting in the selection of a glutaraldehyde-resistant phenotype which could enable it to thrive within the surgical environment in Brazil [7–9, 11–14]. Further evidence that this lineage had adapted to this niche includes increased virulence in mice and the presence of a novel incP-1(beta) plasmid, pMAB01, containing antimicrobial resistance genes [15, 16].

Exactly how the lineage has spread to many hospitals within Brazil remains unclear. Both the involvement of surgeons who carry their own equipment to multiple surgical settings and the dissemination of contaminated commercially available non-activated glutaraldehyde solution have been suggested as possible transmission mechanisms [8, 12]. The outbreak lineage was not solely isolated from post-surgical wound infections, with isolates matching the outbreak PFGE pattern collected from sputum and urine specimens and samples from endoscopes. Some of these isolates were sampled from locations where outbreaks of skin and soft tissue infections had not occurred [10, 17]. This led to speculation that the cause of the outbreaks could be a Brazilian environmental lineage as opposed to a recently introduced lineage that had subsequently adapted to the surgical niche and spread either via surgeons or contaminated disinfectants.

Evidence has also been presented that suggests more than one lineage is responsible for the outbreaks. Whole genome sequencing of isolates from the outbreaks in Goiás and Rio de Janeiro suggested that the outbreak isolates from Rio de Janeiro shared a higher number of core genome SNPs with outbreak isolates obtained from cystic fibrosis patients during an outbreak in the UK as opposed to the outbreak isolate from Goiás, GO-06 [4, 18, 19].

For this study, we sequenced 188 isolates from nine geographically distant states in Brazil in which outbreaks occurred. Our aims were to clarify whether a single or multiple lineages were responsible for these outbreaks and explore whether the genetic data provided support for the spread of an epidemic lineage via the travelling surgeons hypothesis or contamination of inactivated glutaraldehyde hypothesis. We also explored whether there was further genetic evidence that the lineage has specifically adapted to the surgical niche within Brazil.

## Methods

### Isolates and sequencing

DNA was extracted from 188 isolates obtained from skin and soft tissue infections occurring in nine states between 2004 and 2010 with approval from the Universidade Federal de São Paulo ethics committee (0068/12). The isolates were sequenced via Illumina Hiseq 2500 (Table S1). This dataset was supplemented with 526 sequences from a global population study [2] and three publicly available isolates GO-06 (GenBank accession number CP003699.2), CRM-0020 (ATFQ00000000.1) and 47J26 (AGQU00000000.1) (Table S1, available in the online Supplementary Material).

### Mapping, *de novo* assembly and annotation

The sequences were *de novo* assembled using the short read assembler Velvet and Velvet Optimiser [20]. Annotation of the assemblies was performed with the software Prokka via an automated in-house pipeline [21]. The annotations were supplemented by blast searches against the InterPro, Pfam and PHAST databases. Gene Ontology terms (GO-terms) were determined with Blast2GO [22].

The raw reads of the isolates were mapped to *M. abscessus* ATCC 19977 (GenBank accession number CU458896.1) and to a reference selected from the newly sequenced isolates, BRA_PA_42 (GenBank accession number GCA_900169205), using bwa-mem [23]. BRA_PA_42 consisted of two contigs, with the chromosome present in a single contig. Variant sites were identified using Samtools v1.2 and bcftools v1.2 and filtered as previously described [24] (see Supplementary Materials).

### Phylogenetic analysis

Two maximum-likelihood phylogenetic trees, with 100 bootstrap replicates, were reconstructed using RAxML [25]. Minimum Spanning Trees (MST) were reconstructed using the goeBURST algorithm within Phyloviz [26] (see Supplementary Materials).

### Temporal analysis

A recombination-free alignment, 4 686 059 bp in length, of the 173 Brazilian isolates with full day/month/year dates available was produced using Gubbins [27]. A phylogeny was reconstructed from the 144 variant positions, extracted with SNP_sites [28], using RAxML [25]. The phylogenetic root-to-tip distance was regressed against the sampling date. Isolation dates were permuted 1000 times to obtain the statistical significance of the regression [29]. This alignment was also used to date the Brazilian lineage using beast v1.8.3 [30]. BEAUti was used to configure the model and create the xml file required by beast (see Supplementary Materials).

### Genome comparisons

Whole genome comparisons were carried out using nucleotide blast (blastn) (v 2.4.0) [31] and visualised using the Artemis Comparison Tool (act) [32]. Assemblies consisting of fewer than 100 contigs and with lengths that fell within 1.5 times the interquartile range were used to compare the genome size of the three subspecies and the Brazilian lineage. The novel contigs within the Brazilian lineage were compared against the GenBank nr/nt database using blastn. A Local blastn search and a mapping approach were used to determine the prevalence of the novel contigs amongst all the isolates used in this study (see Supplementary Materials). The average nucleotide identity (ANI) and average amino acid identity (AAI) of the type VII secretion system (T7SS) found on one novel contig was compared to those previously described [33, 34].

## Results

### One lineage of *M. a*. *massiliense* is responsible for the epidemic of post-surgical wound infections in Brazil

**Fig. 1.**
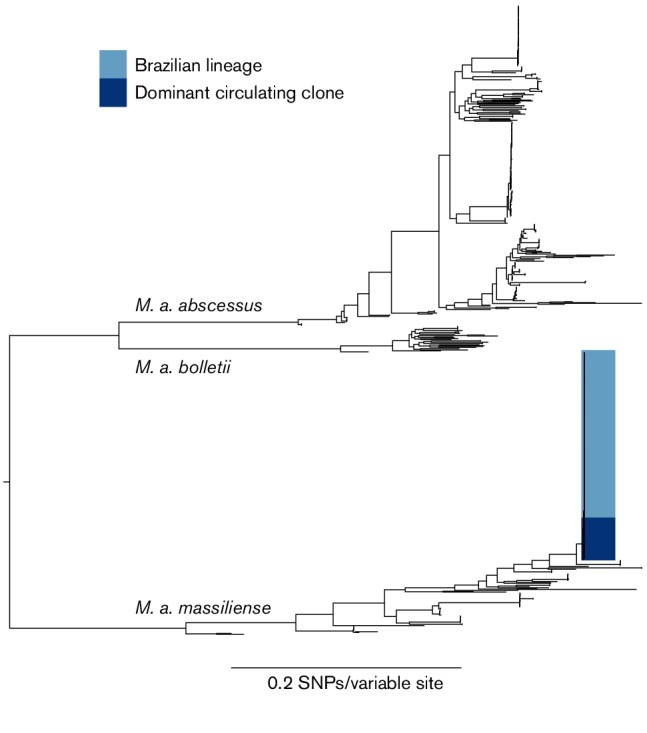
Midpoint rooted maximum-likelihood phylogeny of the global population of the *Mycobacterium abscessus* species complex. The scale bar represents the number of nucleotide substitutions per site. This shows that the Brazilian isolates form a single clade (dark blue) closely related to a recently described dominant circulating clone (DCC) (light blue).

**Fig. 2.**
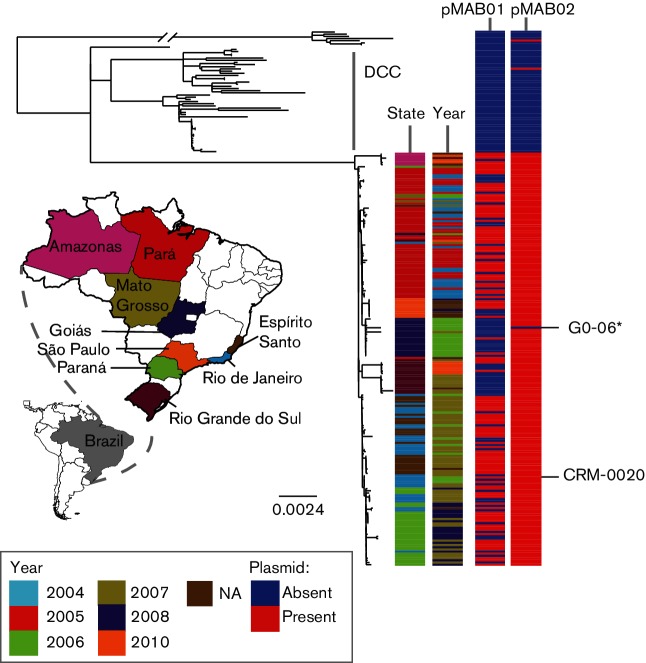
Maximum-likelihood tree, rooted to an outgroup, of the clades containing the Brazilian lineage and dominant circulating clone (DCC). The scale bar represents the number of nucleotide substitutions per site. The first two metadata columns show the state and time (year) when the isolates were collected and reveals evidence of isolates clustering by state and year of collection. Metadata columns 3 and 4 indicate the presence (red) or absence (blue) of the two plasmids associated with the Brazilian lineage, pMAB01 and the newly described pMAB02. This shows that pMAB02 is found throughout the Brazilian lineage, but pMAB01 is not present in all the Brazilian isolates. *GO-06 was re-sequenced in this study, BRA_GO-06, and found to contain pMAB02, however, the current publicly available version did not contain pMAB02.

The Brazilian isolates formed a single clade, to the exclusion of all other isolates of *M. abscessus*, within the MABSC phylogeny, demonstrating that the epidemic was caused by a single lineage. In addition, we found that they were closely related to a recently described dominant circulating clone (DCC) within the *M. a. massiliense* subspecies (Fig. 1) [2]. Re-mapping sequence data from these isolates to a local reference showed that there were 149 SNPs between the Brazilian lineage and the DCC. This phylogeny also showed that there was geographical and temporal structure within the Brazilian lineage (Fig. 2).

### The Brazilian lineage emerged recently

**Fig. 3.**
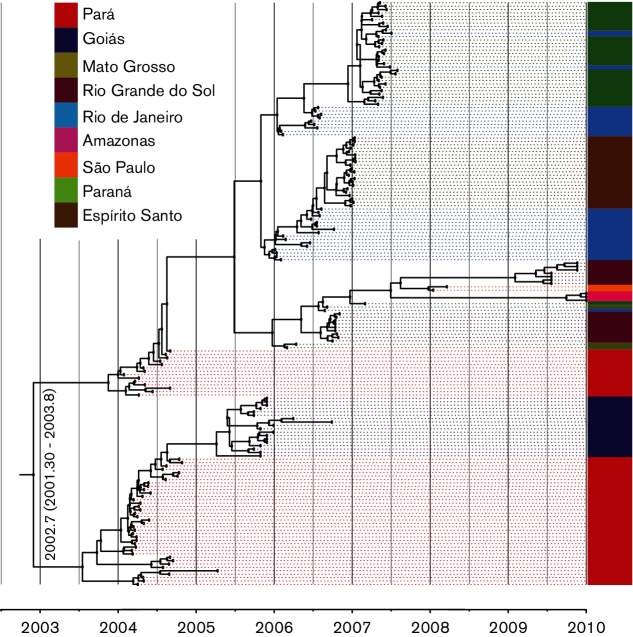
Maximum Clade Credibility tree of the Brazilian lineage inferred from the 27 003 trees produced by beast after removing burnin, showing the estimated date of the emergence of the Brazilian lineage to be around 2003. The colours show the state from which the isolates were collected.

We examined whether there was a temporal signal within the Brazilian lineage using root-to-tip linear regression analysis [29]. This showed that there was a significant signal (r^2^=0.802, *P*-value=0.0009) within the data (Fig. S1) and indicated that there was a strong enough signal to gain a more accurate estimation of the date of the emergence of the lineage via beast [30]. The beast analyses estimated that the Brazilian lineage emerged in 2003 (2002.7; upper 95 %: 2003.8, lower 95 %: 2001.3) (Fig. 3), just prior to the initial outbreak in Pará in 2004, with the mean substitution rate estimated to be 8.25×10^−07^ SNPs/site/year (upper 95 %: 1.07×10^−06^, lower 95 %: 5.31×10^−07^), around 3.8 SNPs/genome/year (2.4–5.0), similar to that previously measured for a DCC within *M. a. abscessus* [3.5×10^−7^ SNPs/site/year (1.83–5.11×10^−7^)] [2].

### Onward spread throughout Brazil

**Fig. 4.**
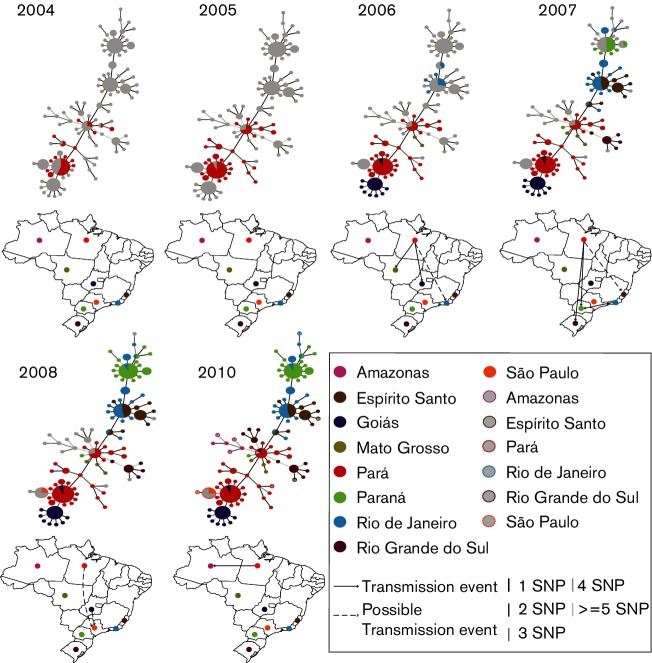
Minimum Spanning Trees (MSTs) showing the transmission route suggested by the genetic data. The colours represent the state from which the isolates were collected and the node sizes are representative of the number of isolates with that genotype. Open circles represent isolates with a known location, but no recorded date. From this analysis, it can be seen that transmission events from Pará introduced the Brazilian lineage to Goiás, Mato Grosso and either Rio de Janeiro or Espírito Santo in 2006, Rio Grande do Sul and Paraná in 2007 and Amazonas in 2010. The outbreak in São Paulo in 2008 was seeded either from Goiás or Pará. A transmission event from Rio de Janeiro to Paraná in 2007 is shown to be responsible for the majority of cases in this region.

Minimum spanning trees were used to identify the potential transmission route of the lineage between states. Fig. 4 shows that Pará was the main hub for transmission and that transmission events from Pará seeded the cases seen in Goiás (2006), Mato Grosso (2006), Rio Grande do Sul (2007) and Amazonas (2010). There is evidence that a transmission event also occurred between Pará and Paraná. However, the majority of the cases in Paraná represented a transmission event from Rio de Janeiro. The transmission event(s) that resulted in the introduction of the lineage to Espírito Santo and Rio de Janeiro is less clear. Whilst the source was Pará, it is not possible to identify whether this was initially to Espírito Santo or Rio de Janeiro. Similarly, it is not possible to tell whether the lineage was introduced into São Paulo from Pará or Goiás.

### Genomic Evidence that the Brazilian lineage has undergone adaption to a novel niche

**Fig. 5.**
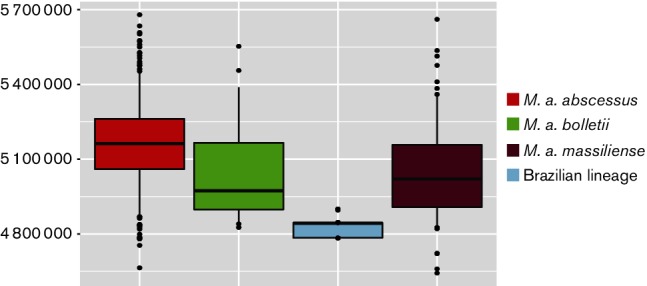
Boxplots comparing the genome sizes of 352 *M* *. a. abscessus* genomes, 30 *M* *. a. bolletii* genomes, 130 *M* *. a. massiliense* genomes and 188 Brazilian lineage genomes. The borders of the boxplot represent the 25^th^ and 75^th^ percentile. The bold line represents the median genome size. The whiskers mark the 5^th^ and 95^th^ percentile. This shows that the genome size of the Brazilian lineage (light blue) is significantly smaller than the genome sizes of the three subspecies.

Given that the genetic data demonstrates that the outbreaks in Brazil are caused by a single lineage, we investigated whether there was evidence that it has adapted to the surgical niche within Brazil. Fig. 5 shows that the Brazilian lineage genomes are 298 431 bps smaller on average than the average genome size of the three subspecies. This is potentially an indication that the Brazilian lineage has undergone reductive evolution.

**Fig. 6.**
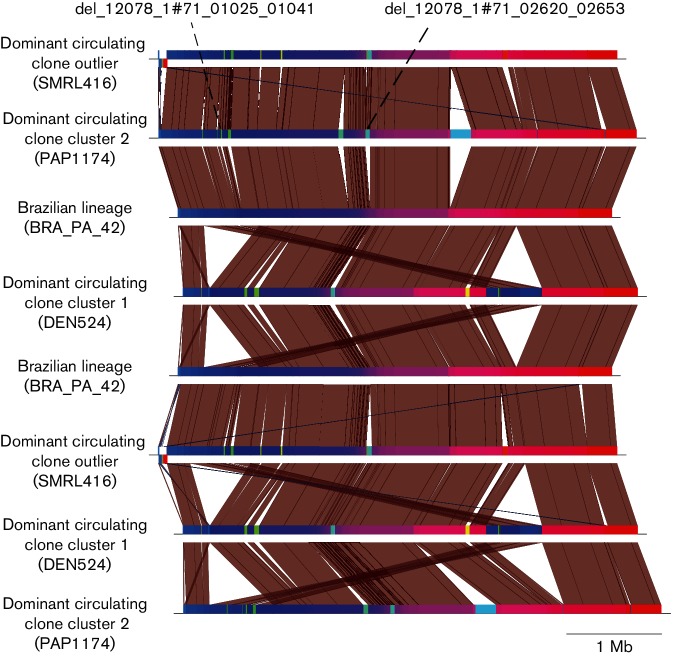
A nucleotide blast comparison between a representative of the Brazilian lineage, a representative of the dominant circulating clone (DCC) cluster 1 and cluster 2 and a sporadic *M. a. massiliense* isolate. This shows the deletions that have occurred in the Brazilian lineage that could be indicative of its adaption to a novel environment.

Genome comparisons between representatives of the two sub-clusters within DCC (Fig. S2), the Brazilian lineage and the out-group of these clades revealed that 14 insertions and deletions had occurred (Fig. 6), but that only two deletions were unique to the Brazilian lineage. The two deletions, del_12078_1#71_01025_01041 and del_12078_1#71_02620_02653, consisted of 17 and 52 coding sequences (CDSs), respectively. Tables S2 and S3 show the Prokka, InterPro and GO-term predictions for the two deletions. An annotation was predicted for seven and 49 of the CDSs, respectively. A blastn search against the PHAST database showed that neither of these deletions had nucleotide similarity to any known phage genes. Del_12078_1#71_02620_02653 had 89 % ANI identity to the variable region containing insertion sequence ISMab1 [35]. GO-term analysis showed that 13 CDSs in this deletion were involved in oxidation-reduction processes and nine CDSs were associated with metabolic processes. Del_12078_1#71_01025_01041 begins with a CDS predicted to be a site-specific recombinase. Three CDSs were shown to be involved in DNA integration processes and seven were associated with DNA binding via GO-term analysis. The annotation or potential function of six of the 17 CDSs within this deletion could not be determined.

**Fig. 7.**
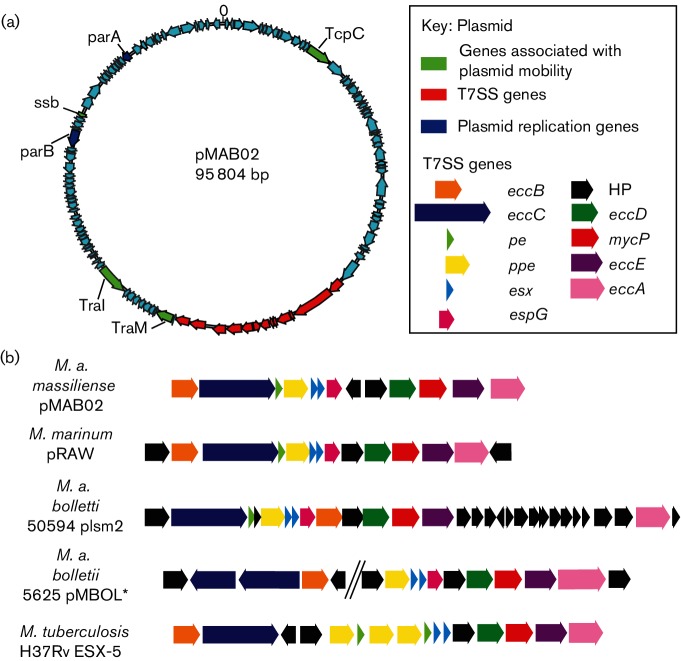
(a) Plasmid map showing the 121 predicted CDSs encoded by pMAB02. The type VII secretion system (T7SS) is shown in red, genes associated with the stable inheritance of pMAB02 are shown in blue, whilst genes that are associated with the mobility of pMAB02 are shown in green. (b) A comparison of the gene order of the T7SS found on pMAB02 with the esxP1 T7SS found on *Mycobacterium marinum* plasmid pRAW and the T7SS that have been described on other *M. abscessus* plasmids plsm2 (*M. a. bolletii* 50 594) and pMBOL1 (*M. a. bolletii* 5625) and the ESX-5 T7SS found on the chromosome of *Mycobacterium tuberculosis* H37Rv. *The assembly of pMBOL is not available and therefore the gene order shown here was based upon that reported by Dumas *et al.* [33].

There was also evidence that the Brazilian lineage had gained genetic material; a newly described 95 804 kb plasmid (Fig. 7a), found within BRA_PA_42, in addition to plasmid pMAB01 [15]. A blastn search of the GenBank database with the second contig showed a complete match (sequence identity 99 %, query coverage 100 %) to a sequence described as *Mycobacterium* phage *Adler* (GenBank accession number KC960489.1). The presence of plasmid genes such as *parA*, *parB*, *traI* and *traM,* a lack of core phage genes (Table S4) and evidence from read pair information that it was circular suggested this contig was most likely a plasmid, which we designated pMAB02.

A local blast search for pMAB01 and pMAB02 within the global population dataset, including the Brazilian isolates, showed that pMAB01 was unique to the Brazilian lineage but was only present in 117 of the Brazilian isolates. Although it is likely that this plasmid may be being lost in culture, there is some evidence of geographical loss of pMAB01, with a cluster of isolates from Rio Grande do Sul all having lost the plasmid (Fig. 2). In contrast, pMAB02 was present in all Brazilian lineage genomes (*n*=189), except the previously sequenced G0-06 [36]. However, this isolate was re-sequenced in this study (BRA_G0_06) and was found to contain pMAB02. pMAB02 was found in five other isolates in the global population (with >99 % ANI) (Figs 2, S2 and S3). The presence of pMAB02 in all Brazilian lineage isolates suggested that it could be conferring a selective advantage upon this lineage and therefore we analysed the gene content of the plasmid to see if there were any genes that could be advantageous. pMAB02 was found to contain 121 CDSs (Fig. 7a and Table S5), 89 of which were hypothetical. It was found to encode plasmid partitioning proteins, *parA* and *parB*, as well as genes involved in conjugation. We also observed the presence of a complete T7SS (Fig. 7a). The T7SS had no significant (E-value <0.05) ANI with the T7SSs found on *M. a. bolletii* 50 594 plasmid 2, *Mycobacterium marinum* plasmid pRAW or the chromosomal ESX-5 T7SS found on *M. tuberculosis* H37Rv. However, the AAI between the T7SS found on pMAB02 and these T7SSs was 36, 38 and 37 %, respectively. The gene order was most similar to the recently described ESX-P1 T7SS, like that encoded by the *M. marinum* plasmid pRAW [34] (Fig. 7b).

## Discussion

Since 2004 there have been over 2000 cases of post-surgical wound infections caused by *M. a. massiliense* in Brazil, including several local outbreaks. To understand the evolutionary success of the outbreak, we performed whole genome sequencing on 188 isolates spanning both the temporal and geographical ranges of the outbreak. Phylogenetic analysis confirmed that a single lineage, which included two previously sequenced isolates, GO-06 and CRM-0020, was responsible for all cases despite their isolation from geographically distant regions of Brazil.

The Brazilian lineage was found to be closely related to a recently described DCC (Fig. 1) that includes isolates from two cystic fibrosis centre pulmonary infection outbreaks [2–4, 18, 19]. The recent emergence of the Brazilian lineage, estimated by beast to be c. 2003, and its close relationship to a DCC in cystic fibrosis patients, suggests that this lineage was recently introduced to Brazil and subsequently adapted to the surgical niche. This supports the hypothesis, suggested previously, that poor practices led to the selection of a glutaraldehyde-tolerant *M. a. massiliense* lineage, which subsequently spread throughout hospitals in Brazil [7, 12, 13]. Whilst the last common ancestor of this lineage could be present in the Brazilian environment, the recent emergence of the lineage and the high level of genetic similarity between isolates from different outbreak locations and between isolates that were collected several years apart is not consistent with this hypothesis. The most likely scenario is direct transmission of the lineage between hospitals rather than the lineage being independently introduced from the environment.

The mechanism by which the outbreak lineage has spread throughout Brazil has been unclear, with two main hypotheses suggested [11, 12]. The lack of epidemiological information on our samples means that it is not possible to conclusively resolve this question; however, the clear geographical and temporal structure within the phylogeny (Fig. 2), is a pattern more consistent with the introduction of the lineage to different locations occurring through transmission, rather than dissemination from a point source, which would be indicated by a star phylogeny without geographical substructure. The vehicle enabling the transmission of the lineage remains unclear, although the contamination of surgeons’ equipment has been suggested previously. However, it was not possible to obtain samples directly from surgeons’ equipment in order to test this hypothesis. Further support for the outbreak lineage being transmitted across Brazil is provided by the outbreaks not occurring contemporaneously, as might be expected if there was contamination originating from a single source of inactivated glutaraldehyde. MSTs showed that the lineage was introduced to the majority of states from Pará, but due to incomplete sampling, it is impossible, from this data, to establish the transmission route that led to every outbreak.

The MSTs also highlight the lineage’s ability to persist in the hospital environment [14], as the outbreak in Pará occurred from 2004–2005 but transmission events continued to emanate from Pará until 2010. This implies that the lineage has adapted to survive within the hospital environment and suggests that there is the potential for further outbreaks to occur if poor practices were to re-emerge [10]. The combination of the ability to persist in the hospital environment and undergo long-distance spread has serious implications for the global spread of *M. a. massiliense* lineages.

We also uncovered evidence that suggests the lineage has begun adapting to a novel environment. The smaller genome size of the Brazilian lineage may be an indication of adaptation to the surgical niche within Brazil (Figs 5, 6 and S2) through reductive evolution. Similar processes have previously been observed on a comparable timescale in pathogens such as *Salmonell* *a* *enterica* subsp. *enterica* serovar Typhimurium and *Salmonella enteritidis*, and on a much longer timescale in the mycobacterial pathogens *Mycobacterium leprae* and *Mycobacterium ulcerans* [37–40]. However, given that the median genome size of the Brazilian lineage falls between the 5th and 95th percentile of the genome sizes for *M. a. massiliense*, it could be argued that this is not outside the expected variation of the genome size across this subspecies and consequently is not evidence of reductive evolution.

Only a small number of unique deletions were detected in the Brazilian lineage, which is in contrast to the large number of insertions in the DCC, which could suggest that the Brazilian lineage now inhabits an environment not as conducive to DNA uptake. GO-term analysis found that 13 and nine CDSs within del_12078_1#71_02620_02653 had functions related to oxidation-reduction and metabolic processes, respectively, suggesting a potential role for this deletion in the degradation of an environmental metabolite, and consequently that its loss could be seen as evidence that the lineage is losing genetic material it no longer requires in its new niche, and thus is undergoing reductive evolution. However, experimental analysis is required to determine the roles the two deleted regions in the Brazilian lineage were performing, and consequently whilst the loss of these genes could suggest this lineage is beginning to adapt to a novel niche, on the basis of the current understanding of the functions of the deletions, coupled with the genome size falling within the 5^th^ and 95^th^ percentile of the genome sizes of *M. a. massiliense*, there isn’t compelling evidence that reductive evolution has occurred.

The gain of genetic material is also often important in adaptation to novel niches and the two plasmids, pMAB01 and pMAB02, in the Brazilian lineage seem likely to be associated with its success. pMAB01, which encodes resistance to antibiotics, mercury and quaternary ammonium compounds, was found to be only present within the Brazilian lineage and was lost in 71 isolates [15]. Although there is potentially evidence of geographical loss (Fig. 2), this is believed to have occurred during subculturing as the Goiás isolates used in this study initially were found to contain the ~50 bp band in PFGE profiles, which corresponds to pMAB01 in the analysis of Cardoso *et al.* [11]. pMAB01 is not responsible for the glutaraldehyde-resistant phenotype displayed by the Brazilian lineage as all the outbreak isolates have been shown to be resistant even if they did not harbour pMAB01. Consequently, it is likely that a chromosomal genetic change, possibly involving porins as is the case in *Mycobacterium chelonae* [41], is responsible for the glutaraldehyde-resistant phenotype although it is also possible that pMAB02 may be involved.

Analysis of pMAB02 showed that 89 out of the 121 CDSs were hypothetical proteins making it difficult to determine the functions this plasmid might perform. The presence of the genes *traI* and *traM* and the fact that pMAB01 is conjugative, suggests pMAB02 is mobilisable. The most interesting finding on pMAB02 was the presence of a complete T7SS, as T7SSs are critical to pathogenicity in *M. tuberculosis*. The pMAB02 T7SS shared 30 % AAI and a conserved gene order with a recently described ESX-P1 T7SS (Fig. 7b) suggesting that these two T7SSs diverged a very long time ago [33]. Experimental analysis is required to determine both the function of the T7SS and the role pMAB02 may be playing in the success of the Brazilian lineage.

We have shown that a single lineage is responsible for the outbreaks of *M. a. massiliense* in geographically distant regions of Brazil, with the genetic data suggesting that the lineage spread to different regions of Brazil via transmission as opposed to dissemination from a single point source. The evidence presented that *M. a. massiliense* is adapting to this new niche and of its ability to undergo long distance transmission events, emphasises its importance as an emerging nosocomial pathogen that is a global threat.

## Data bibliography

1. European Nucleotide Archive ERP006740. http://www.ebi.ac.uk/ena/data/view/PRJEB7058.
2. Bryant JM, Grogono DM, Rodriguez-Rincon D, Everall I, Brown KP *et al*. European Nucleotide Archive ERP001039. http://www.ebi.ac.uk/ena/data/view/PRJEB2779 (2016).
3. Raiol T, Ribeiro GM, Oliveira JVA, Sandes EFO, Melo ACMA *et al.* GenBank. https://www.ncbi.nlm.nih.gov/nuccore/CP003699.2 (2014).
4. Davidson RM, Reynolds PR, Farias-Hesson E, Duarte RS, Jackson M *et al.* GenBank. https://www.ncbi.nlm.nih.gov/nuccore/ATFQ00000000.1 (2013).
5. Chan J, Halachev M, Yates E, Smith G, Pallen M. GenBank. https://www.ncbi.nlm.nih.gov/nuccore/AGQU00000000.1 (2012).

## Supplementary Data

1238 Supplementary File 1
