# Supplementary material for: Genomic epidemiology of a national outbreak of post-surgical Mycobacterium abscessus wound infections in Brazil
Source: Microb Genom. 2017 May 3;3(5):e000111. doi: 10.1099/mgen.0.000111 (PMC5562415; doi:10.1099/mgen.0.000111)
Supplement: Supplementary File 1 [file mgen-3-111-s001.pdf]

**Supplementary Information**

Supplementary information for the manuscript titled: *Genomic epidemiology of a national outbreak of post-surgical Mycobacterium abscessus wound infections in Brazil*

**Supplementary Methods**

**DNA extraction and sequencing**

DNA was prepared from isolated colonies grown in liquid Middlebrook 7H9 medium supplemented with OADC (oleic acid, albumin, dextrose and catalase) (Becton Dickinson) using QIAamp DNA mini kit (Qiagen) according to the manufacturer's recommendations. Illumina libraries were constructed with a 450bp insert size according to Illumina protocols, and used to generate 125bp paired-end sequences on a HiSeq 2500.

**Mapping and SNP calling**

The raw reads of the 188 Brazilian isolates and 526 global population isolates (Bryant et al., manuscript in preparation) (n=714) were mapped to the type strain *M. abscessus* ATCC 19977 (CU458896.1) using BWA-MEM [1], with GATK indel realignment and the minimum identity threshold to report a mapping set at 95%. Variant sites were identified using Samtools v1.2 and bcftools v1.2 [2] and filtered as previously described [3]. To investigate the diversity between the Brazilian isolates and their closest relatives at a higher resolution the Brazilian isolates, the clades containing their closest relatives and publically available sequences from Brazil, GO-06 [4] and CRM-0020 [5], and the UK, 47J26 [6] (n=246), were mapped using BWA-MEM [1], with the parameters described above, to BRA_PA_42, a reference selected from the newly sequenced isolates that consisted of 2 contigs with the chromosome present in a single contig.

**Phylogenetic analysis**

Two maximum likelihood phylogenetic trees, with 100 bootstrap replicates performed were constructed using RAxML [7]. The first was inferred from the 326,792 variable positions extracted, using SNP_sites [8], from the alignment of 714

isolates. The second was inferred from the 1,127 variable positions extracted, using SNP_sites [8], from the alignment of 246 isolates. Minimum Spanning Trees (MST) were inferred using the goeBURST algorithm within Phyloviz [9]. An alignment of the variable positions between 188 Brazilian isolates was prepared using bcftools v1.2 [2], with variants called when the quality score was greater than 10 and at least 4 high quality reads supported the alternative allele. The final alignment consisted of 197 variant sites, which were assigned to 97 genotypes using the adegenet and poppr R packages [10, 11].

**Temporal analysis**

To gain a more accurate estimation of the date of emergence of the Brazilian lineage we used BEAST v1.8.3[12]. BEAUti was used to configure the model and create the xml file required by BEAST. A generalised time reversible substitution model and a GAMMA site heterogeneity model with a relaxed log normal clock and constant population size were selected. A diffuse gamma distribution (shape 0.001, scale 1000) was selected as prior for the substitution rate (ucld mean). The length of the Monte Carlo Markov chain (MCMC) was set at 100,000,000 sampling every 10,000$^{th}$ generation. Three independent BEAST instances were run, with results compared using Tracer and combined using the LogCombiner. A maximum clade credibility (MCC) tree was constructed using TreeAnnotator from the 27,003 trees remaining after a burnin of 1,000 trees per run and keeping a posterior probability limit of 0.5l.

**Identification and analysis of the novel plasmid**

The reference selected from the isolates sequenced in this study, BRA_PA_42, consisted of two contigs with the chromosome present in a single contig. A nucleotide BLAST (BLASTn) comparison was carried out between the second contig found in

our chosen reference, BRA_PA_42, and pMAB01 (CP003376.1). This contig was found not to be pMAB01 so we investigated whether it was a novel plasmid. The second contig was compared, using BLAST, against the NCBI Genbank nt/nr database and against the other Brazilian isolates and the global population dataset. The gene order, average nucleotide identity (ANI) and average amino acid identity (AAI) of the Type VII secretions system (T7SS) found on the second contig was compared to the T7SSs found on plasmids recently described by Ummels et al., (2014) and Dumas et al., (2016) as well as the chromosomal ESX-5 T7SS found in *M. tuberculosis* H37Rv [13, 14]. The ANI and AAI were calculated using nucleotide BLAST and tBLASTx (Evalue <=1E-05 and match length >= 100).

The prevalence of pMAB01 within our newly sequenced Brazilian isolates and across our global population dataset was determined by both mapping the raw reads, as described above, to the reference of pMAB01, and by performing a BLAST search of all the assemblies for this plasmid.

**Figure preparation**

All the phylogenetic trees presented in this study were visualised using Figtree v.1.4.2 [15]. The maps used in figure 2 were downloaded from d-maps[16]. Figure 6 was produced by aligning the genomes with progressive Mauve [17] and plotted using genoPlotR [18]. The boxplots to compare the genome sizes were created using the ggplot2 [19] package in R. The plasmid map in figure 7 was drawn using SnapGene [20]. Supplementary Figure 2 was produced with ITOL v.3.2.4[21].

**References:**


1. **Li H**. Aligning sequence reads, clone sequences and assembly contigs with BWA-MEM. *arXiv*. 2013;0:1-3.

2. **Li H, Handsaker B, Wysoker A, Fennell T, Ruan J *et al.*** The Sequence Alignment/Map format and SAMtools. *Bioinformatics*. 2009;25(16):2078-9.

3. **Harris SR, Feil EJ, Holden MTG, Quail MA, Nickerson EK *et al.*** Evolution of MRSA During Hospital Transmission and Intercontinental Spread. *Science (New York, NY)*. 2010;327(5964):469-74.

4. **Raiol T, Ribeiro GM, Maranhão AQ, Bocca AL, Silva-Pereira I *et al.*** Complete Genome Sequence of Mycobacterium massiliense. *Journal of Bacteriology*. 2012;194(19):5455.

5. **Davidson RM, Reynolds PR, Farias-Hesson E, Duarte RS, Jackson M *et al.*** Genome Sequence of an Epidemic Isolate of Mycobacterium abscessus subsp. bolletii from Rio de Janeiro, Brazil. *Genome Announc*. 2013;1(4).

6. **Chan J, Halachev M, Yates E, Smith G, Pallen M**. Whole-genome sequence of the emerging pathogen Mycobacterium abscessus strain 47J26. *J Bacteriol*. 2012;194(2):549.

7. **Stamatakis A**. RAxML version 8: a tool for phylogenetic analysis and post-analysis of large phylogenies. *Bioinformatics*. 2014;30(9):1312-3.

8. **Page AJ, Taylor B, Delaney AJ, Soares J, Seemann T *et al.*** SNP-sites: rapid efficient extraction of SNPs from multi-FASTA alignments. *Microbial Genomics*. 2016;2(4).

9. **Francisco AP, Vaz C, Monteiro PT, Melo-Cristino J, Ramirez M *et al.*** PHYLOViZ: phylogenetic inference and data visualization for sequence based typing methods. *BMC Bioinformatics*. 2012;13(1):1-10.

10. **Jombart T**. adegenet: a R package for the multivariate analysis of genetic markers. *Bioinformatics*. 2008;24(11):1403-5.

11. **Kamvar ZN, Tabima JF, Grunwald NJ**. Poppr: an R package for genetic analysis of populations with clonal, partially clonal, and/or sexual reproduction. *PeerJ*. 2014;2:e281.

12. **Drummond AJ, Rambaut A**. BEAST: Bayesian evolutionary analysis by sampling trees. *BMC Evolutionary Biology*. 2007;7(1):1-8.

13. **Ummels R, Abdallah AM, Kuiper V, Aâjoud A, Sparrius M *et al***. Identification of a Novel Conjugative Plasmid in Mycobacteria That Requires Both Type IV and Type VII Secretion. *mBio*. 2014;5(5):e01744.

14. **Dumas E, Boritsch EC, Vandenbogaert M, Rodríguez de la Vega RC, Thiberge J-M *et al***. Mycobacterial Pan-Genome Analysis Suggests Important Role of Plasmids in the Radiation of Type VII Secretion Systems. *Genome Biology and Evolution*. 2016;8(2):387-402.

15. **Rambaut A.** FigTree. Available from: http:// tree.bio.ed.ac.uk/software/figtree/.FigTree version 1.2.4

16. d-maps. Available from: http://www.d-maps.com/.d-maps

17. **Darling AE, Mau B, Perna NT**. progressiveMauve: Multiple Genome Alignment with Gene Gain, Loss and Rearrangement. *PLoS ONE*. 2010;5(6):e11147.

18. **Guy L, Roat Kultima J, Andersson SGE**. genoPlotR: comparative gene and genome visualization in R. *Bioinformatics*. 2010;26(18):2334-5.

19. **Wickham H.** ggplot2: Elegant Graphics for Data Analysis: Springer-Verlag New York; 2009 2009.

20. SnapGene. [Available from: http://www.snapgene.com/products/snapgene_viewer/.SnapGene software

21. **Letunic I, Bork P**. Interactive tree of life (iTOL) v3: an online tool for the display and annotation of phylogenetic and other trees. *Nucleic Acids Res*. 2016;44(W1):W242-5.

**Supplementary Tables:**

**Supplementary Table 1: Information on sequences**

File name: *supplementary_table_1.docx*

**Supplementary Table 2: Annotation of Del_12078_1#71_01025_01041**

File name: *supplementary_table_2.docx*

**Supplementary Table 3: Annotation of Del_12078_1#71_02620_02653**

File name: *supplementary_table_3.docx*

**Supplementary Table 4: Annotation of the CDSs present on pMAB02**

File name: *supplementary_table_4.docx*

**Supplementary Figures:**

**Supplementary Figure 1:**

A linear regression plot of the root-to-tip distance against the sampling dates. This shows that there was a significant temporal signal within the Brazilian lineage ($r^2$=0.8, permutation test p-value = 0.0009) and suggests that the Brazilian lineage emerged in 2003.

**Supplementary Figure 2:**

Rooted maximum likelihood phylogeny of the Brazilian lineage (light blue), closely related dominant circulating clone (DCC) (dark blue), which has two subclusters, and an outgroup clade (light grey) with the genome sizes of the isolates displayed in the metadata column. The bars coloured gold where those used for the ACT comparison figure (Figure 6).

**Supplementary Figure 3:**

Midpoint rooted maximum likelihood phylogeny of 714 *M. abscessus* species complex isolates with a metadata column showing the presence (red) or absence (blue) of pMAB02 and pMAB01 across the global population. The scale bar represents the number of nucleotide substitutions per site. pMAB02 was found within 189 Brazilian lineage isolates along with 5 isolates within the rest of the global population, 4 *M. a. massiliense* isolates and 1 *M. a. abscessus* isolate. pMAB01 was only present in 117 the Brazilian lineage isolates and no other isolates in the rest of the global population.

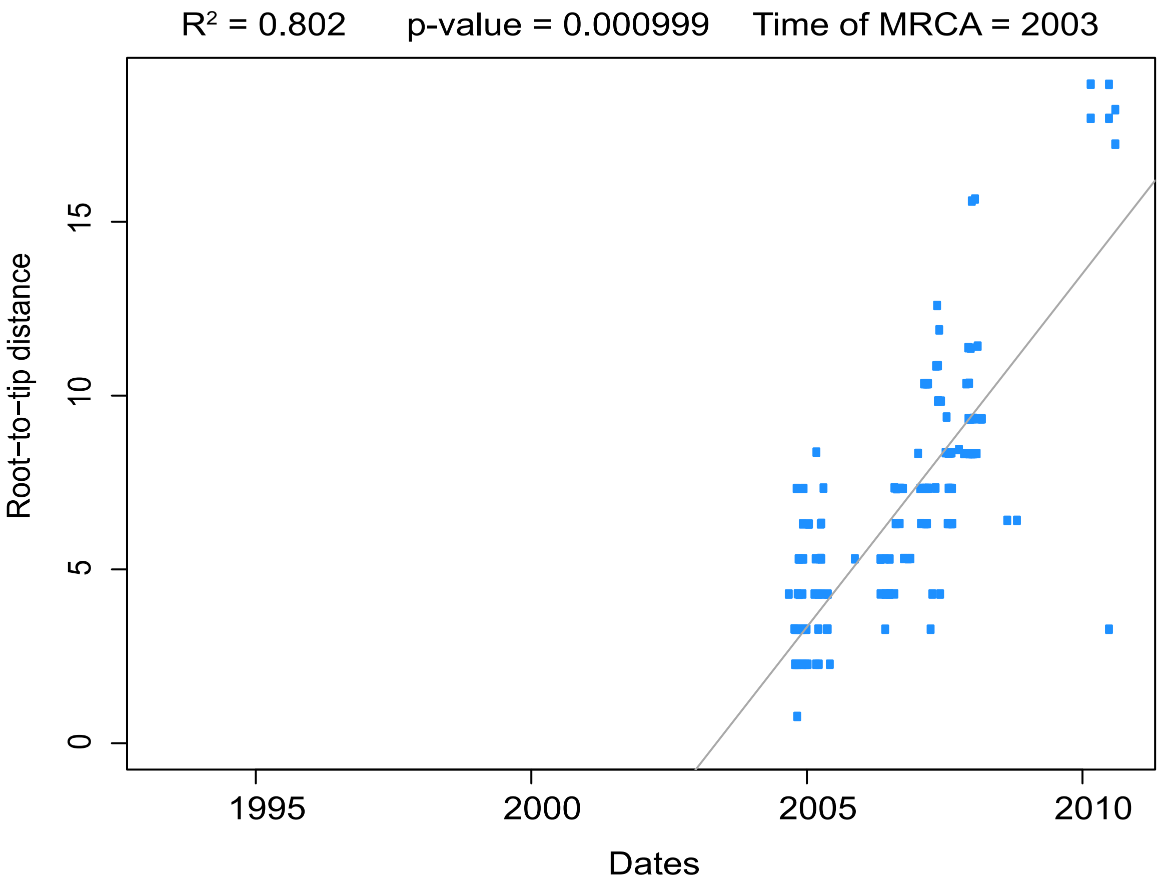

R² = 0.802
p-value = 0.000999
Time of MRCA = 2003
Root–to–tip distance
0
5
10
15
1995
2000
2005
2010
Dates

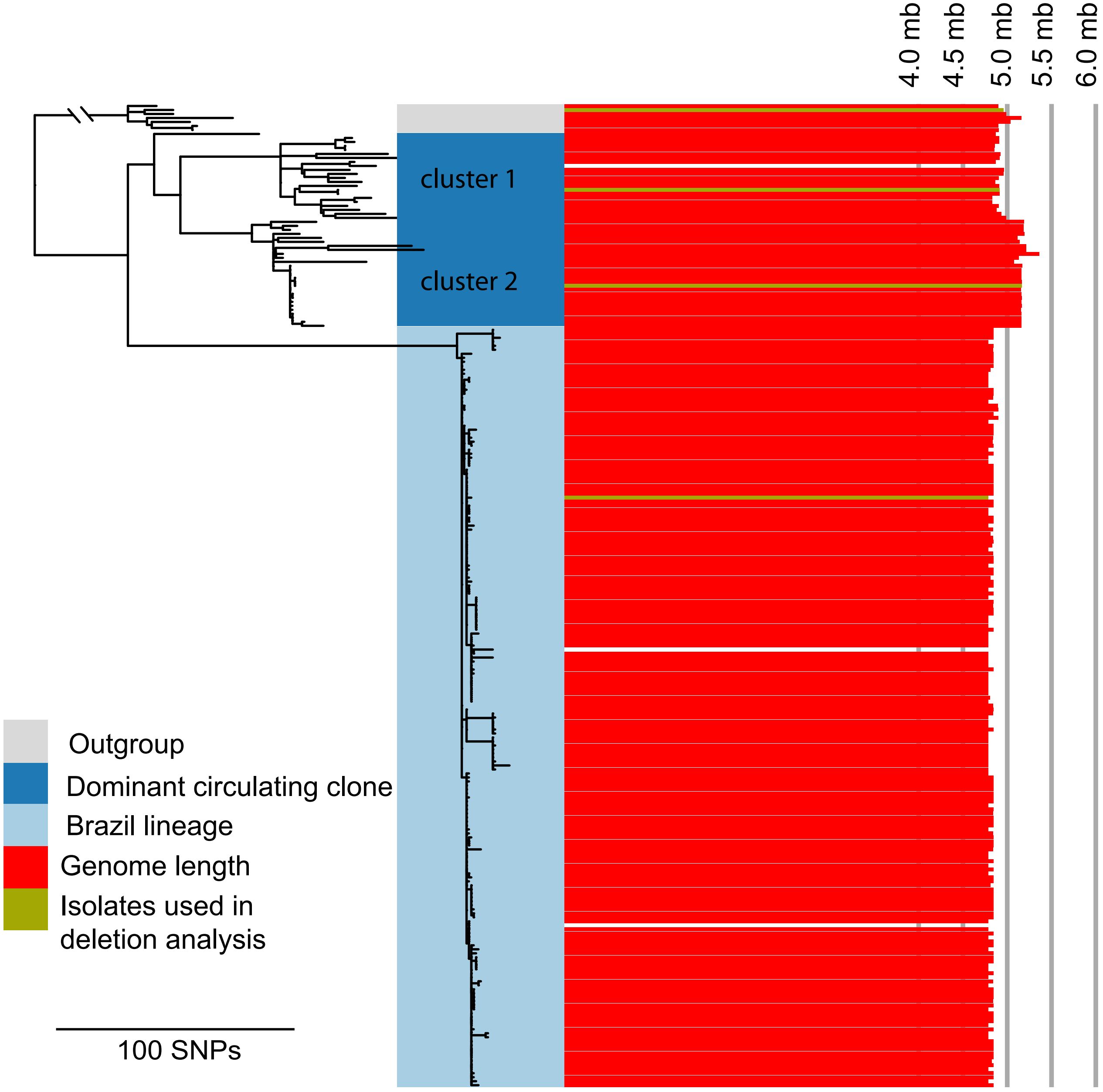
4.0 mb
4.5 mb
5.0 mb
5.5 mb
6.0 mb
cluster 1
cluster 2
Outgroup
Dominant circulating clone
Brazil lineage
Genome length
Isolates used in deletion analysis
100 SNPs

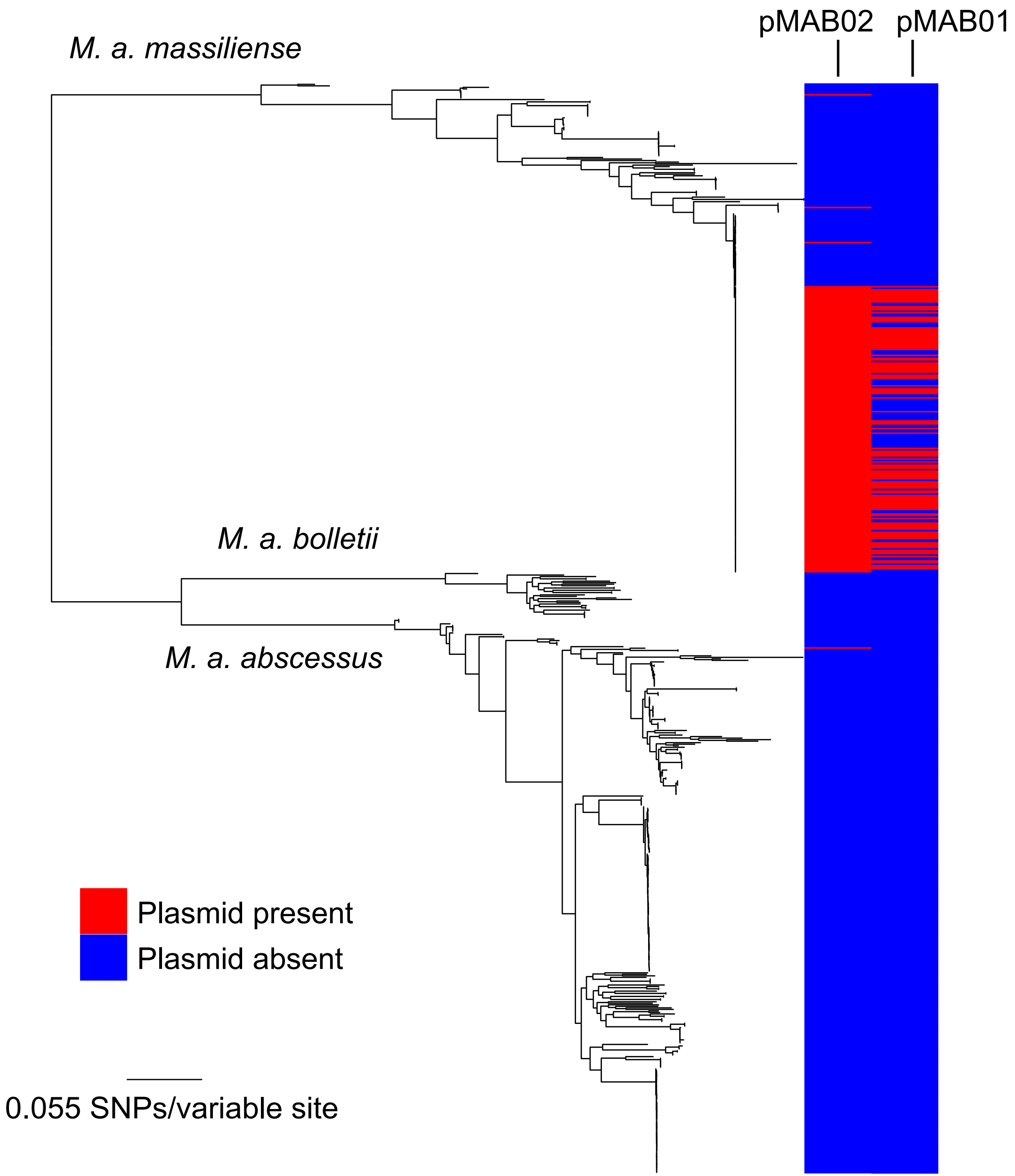

pMAB02
pMAB01
M. a. massiliense
M. a. bolletii
M. a. abscessus
Plasmid present
Plasmid absent
0.055 SNPs/variable site

**Supplementary Table 1:** Summary of the whole genome sequences used in this study

| Lane | Accession | Supplier | Lancet id† | City | State | Country | Day/Month/Year | Reference | Sequencing platform |
|---|---|---|---|---|---|---|---|---|---|
| NA | AGQU00000000.1 | 47J26* | NA | NA | NA | UK | 2009 | Chan et al., (2011) | GS flx (454) |
| NA | ATFQ00000000.1 | CRM_020* | NA | NA | RJ | Brazil | 2006 | Davidson et al., (2013) | abi solid |
| NA | CP003699.2 | GO_06* | NA | NA | GO | Brazil | 2006 | Raiol et al., (2012) | GS flx (454) |
| 7520_7#40 | ERR119097 | PAP020n | 3n | NA | NA | UK | 2010 | Bryant et al., (2013) | illumina HiSeq |
| 7520_7#38 | ERR119095 | PAP017b | 18b | NA | NA | UK | 2009 | Bryant et al., (2013) | illumina HiSeq |
| 7396_8#92 | ERR115104 | PAP012d | 9d | NA | NA | UK | 2007 | Bryant et al., (2013) | illumina HiSeq |
| 7396_8#88 | ERR115100 | PAP009b | 8b | NA | NA | UK | 2007 | Bryant et al., (2013) | illumina HiSeq |
| 7396_8#80 | ERR115092 | PAP025c | 29c | NA | NA | UK | 2011 | Bryant et al., (2013) | illumina HiSeq |
| 7396_8#78 | ERR115090 | PAP026a | 30a | NA | NA | UK | 2011 | Bryant et al., (2013) | illumina HiSeq |
| 7396_8#74 | ERR115086 | PAP031ad | 2ad | NA | NA | UK | 2011 | Bryant et al., (2013) | illumina HiSeq |
| 7396_8#69 | ERR115081 | PAP001a | 27a | NA | NA | UK | 2010 | Bryant et al., (2013) | illumina HiSeq |
| 7396_8#67 | ERR115079 | PAP011g | 1g | NA | NA | UK | 2010 | Bryant et al., (2013) | illumina HiSeq |
| 7396_8#65 | ERR115077 | PAP021b | 31b | NA | NA | UK | 2011 | Bryant et al., (2013) | illumina HiSeq |
| 7396_8#64 | ERR115076 | PAP030i | 22i | NA | NA | UK | 2011 | Bryant et al., (2013) | illumina HiSeq |
| 7396_8#63 | ERR115075 | PAP028i | 14i | NA | NA | UK | 2011 | Bryant et al., (2013) | illumina HiSeq |
| 7396_8#62 | ERR115074 | PAP022f | 28f | NA | NA | UK | 2011 | Bryant et al., (2013) | illumina HiSeq |
| 7396_8#58 | ERR115070 | PAP027a | 15a | NA | NA | UK | 2010 | Bryant et al., (2013) | illumina HiSeq |
| 7396_8#57 | ERR115069 | PAP024r | 19r | NA | NA | UK | 2010 | Bryant et al., (2013) | illumina HiSeq |
| 7396_8#56 | ERR115068 | PAP019h | 20h | NA | NA | UK | 2010 | Bryant et al., (2013) | illumina HiSeq |
| 7396_8#54 | ERR115066 | PAP033p | 5p | NA | NA | UK | 2010 | this study | illumina HiSeq |
| 7396_8#51 | ERR115063 | PAP032a | 10a | NA | NA | UK | 2010 | Bryant et al., (2013) | illumina HiSeq |
| 7396_7#42 | ERR115054 | PAP006a | 26a | NA | NA | UK | 2010 | Bryant et al., (2013) | illumina HiSeq |

| | | | | | | | | | |
|---|---|---|---|---|---|---|---|---|---|
| 7396_7#40 | ERR115052 | PAP002a | NA | NA | NA | UK | 2010 | this study | illumina HiSeq |
| 7396_7#38 | ERR115050 | PAP007g | 12g | NA | NA | UK | 2010 | Bryant et al., (2013) | illumina HiSeq |
| 7396_7#37 | ERR115049 | PAP003a | 24a | NA | NA | UK | 2010 | Bryant et al., (2013) | illumina HiSeq |
| 7396_7#35 | ERR115047 | PAP004d | 21d | NA | NA | UK | 2010 | Bryant et al., (2013) | illumina HiSeq |
| 7396_7#27 | ERR115039 | PAP005a | 23a | NA | NA | UK | 2010 | Bryant et al., (2013) | illumina HiSeq |
| 7396_7#23 | ERR115035 | PAP008b | 32b | NA | NA | UK | 2007 | Bryant et al., (2016) Manuscript in preperation | illumina HiSeq |
| 7396_7#22 | ERR115034 | PAP023b | 13b | NA | NA | UK | 2010 | Bryant et al., (2013) | illumina HiSeq |
| 7396_7#14 | ERR115026 | PAP015e | 11e | NA | NA | UK | 2009 | Bryant et al., (2016) Manuscript in preperation | illumina HiSeq |
| 7396_7#10 | ERR115022 | PAP010a | 17a | NA | NA | UK | 2009 | Bryant et al., (2016) Manuscript in preperation | illumina HiSeq |
| 7396_6#69 | ERR114985 | PAP016b | NA | NA | NA | UK | 2007 | Bryant et al., (2016) Manuscript in preperation | illumina HiSeq |
| 7396_6#68 | ERR114984 | PAP018d | 7d | NA | NA | UK | 2007 | Bryant et al., (2013) | illumina HiSeq |
| 7396_6#52 | ERR114968 | PAP013a | 6a | NA | NA | UK | 2007 | Bryant et al., (2013) | illumina HiSeq |
| 16933_5#95 | ERR1045722 | BRA_SP_09 | NA | Assis | SP | Brazil | NA | this study | Illumina HiSeq |
| 16933_5#94 | ERR1045721 | BRA_SP_08 | NA | Assis | SP | Brazil | 01/09/2008 | this study | Illumina HiSeq |
| 16933_5#93 | ERR1045720 | BRA_SP_07 | NA | Assis | SP | Brazil | NA | this study | Illumina HiSeq |
| 16933_5#92 | ERR1045719 | BRA_SP_06 | NA | Assis | SP | Brazil | 04/11/2008 | this study | Illumina HiSeq |
| 16933_5#91 | ERR1045718 | BRA_SP_05 | NA | Assis | SP | Brazil | NA | this study | Illumina HiSeq |
| 16933_5#90 | ERR1045717 | BRA_SP_04 | NA | Assis | SP | Brazil | NA | this study | Illumina HiSeq |
| 16933_5#9 | ERR1045637 | BRA_PA_51 | NA | Belém | PA | Brazil | 16/12/2004 | this study | Illumina HiSeq |
| 16933_5#89 | ERR1045716 | BRA_SP_03 | NA | Assis | SP | Brazil | NA | this study | Illumina HiSeq |
| 16933_5#88 | ERR1045715 | BRA_SP_02 | NA | Assis | SP | Brazil | NA | this study | Illumina HiSeq |
| 16933_5#87 | ERR1045714 | BRA_SP_01 | NA | Assis | SP | Brazil | NA | this study | Illumina HiSeq |
| 16933_5#86 | ERR1045713 | BRA_RS2_07 | NA | Carazinho | RS | Brazil | 08/03/2010 | this study | Illumina HiSeq |
| 16933_5#85 | ERR1045712 | BRA_RS2_06 | NA | Carazinho | RS | Brazil | 08/03/2010 | this study | Illumina HiSeq |
| 16933_5#84 | ERR1045711 | BRA_RS2_05 | NA | Carazinho | RS | Brazil | 08/03/2010 | this study | Illumina HiSeq |

| | | | | | | | | | |
|---|---|---|---|---|---|---|---|---|---|
| 16933_5#83 | ERR1045710 | BRA_RS2_04 | NA | Carazinho | RS | Brazil | 08/03/2010 | this study | Illumina HiSeq |
| 16933_5#82 | ERR1045709 | BRA_RS2_03 | NA | Carazinho | RS | Brazil | 07/07/2010 | this study | Illumina HiSeq |
| 16933_5#81 | ERR1045708 | BRA_RS2_02 | NA | Carazinho | RS | Brazil | 07/07/2010 | this study | Illumina HiSeq |
| 16933_5#80 | ERR1045707 | BRA_RS2_01 | NA | Carazinho | RS | Brazil | 07/07/2010 | this study | Illumina HiSeq |
| 16933_5#8 | ERR1045636 | BRA_PA_50 | NA | Belém | PA | Brazil | 14/12/2004 | this study | Illumina HiSeq |
| 16933_5#79 | ERR1045706 | BRA_RS1_10 | NA | Santo Ãngelo | RS | Brazil | 31/05/2007 | this study | Illumina HiSeq |
| 16933_5#78 | ERR1045705 | BRA_RS1_09 | NA | Santo Ãngelo | RS | Brazil | NA | this study | Illumina HiSeq |
| 16933_5#77 | ERR1045704 | BRA_RS1_08 | NA | Santo Ãngelo | RS | Brazil | 31/05/2007 | this study | Illumina HiSeq |
| 16933_5#76 | ERR1045703 | BRA_RS1_07 | NA | Santo Ãngelo | RS | Brazil | 31/05/2007 | this study | Illumina HiSeq |
| 16933_5#75 | ERR1045702 | BRA_RS1_06 | NA | Santo Ãngelo | RS | Brazil | 31/05/2007 | this study | Illumina HiSeq |
| 16933_5#74 | ERR1045701 | BRA_RS1_05 | NA | Santo Ãngelo | RS | Brazil | 20/06/2007 | this study | Illumina HiSeq |
| 16933_5#73 | ERR1045700 | BRA_RS1_03 | NA | Santo Ãngelo | RS | Brazil | 08/06/2007 | this study | Illumina HiSeq |
| 16933_5#72 | ERR1045699 | BRA_RS1_02 | NA | Santo Ãngelo | RS | Brazil | 05/06/2007 | this study | Illumina HiSeq |
| 16933_5#71 | ERR1045698 | BRA_RS1_01 | NA | Santo Ãngelo | RS | Brazil | 18/05/2007 | this study | Illumina HiSeq |
| 16933_5#70 | ERR1045697 | BRA_RJ_30 | NA | Rio de Janeiro | RJ | Brazil | 25/05/2007 | this study | Illumina HiSeq |
| 16933_5#7 | ERR1045635 | BRA_PA_49 | NA | Belém | PA | Brazil | 13/12/2004 | this study | Illumina HiSeq |
| 16933_5#68 | ERR1045696 | BRA_RJ_28 | NA | Angra dos Reis | RJ | Brazil | NA | this study | Illumina HiSeq |
| 16933_5#67 | ERR1045695 | BRA_RJ_27 | NA | Angra dos Reis | RJ | Brazil | 11/02/2008 | this study | Illumina HiSeq |
| 16933_5#66 | ERR1045694 | BRA_RJ_26 | NA | Angra dos Reis | RJ | Brazil | 18/02/2008 | this study | Illumina HiSeq |
| 16933_5#65 | ERR1045693 | BRA_RJ_25 | NA | Rio de Janeiro | RJ | Brazil | 26/03/2007 | this study | Illumina HiSeq |
| 16933_5#64 | ERR1045692 | BRA_RJ_24 | NA | Rio de Janeiro | RJ | Brazil | 27/02/2007 | this study | Illumina HiSeq |
| 16933_5#63 | ERR1045691 | BRA_RJ_23 | NA | Rio de Janeiro | RJ | Brazil | 12/03/2007 | this study | Illumina HiSeq |
| 16933_5#62 | ERR1045690 | BRA_RJ_22 | NA | Rio de Janeiro | RJ | Brazil | 19/03/2007 | this study | Illumina HiSeq |
| 16933_5#61 | ERR1045689 | BRA_RJ_21 | NA | Rio de Janeiro | RJ | Brazil | 07/02/2007 | this study | Illumina HiSeq |
| 16933_5#60 | ERR1045688 | BRA_RJ_20 | NA | Rio de Janeiro | RJ | Brazil | 06/03/2007 | this study | Illumina HiSeq |

| | | | | | | | | | |
|---|---|---|---|---|---|---|---|---|---|
| 16933_5#6 | ERR1045634 | BRA_PA_48 | NA | Belém | PA | Brazil | 10/12/2004 | this study | Illumina HiSeq |
| 16933_5#59 | ERR1045687 | BRA_RJ_18 | NA | Rio de Janeiro | RJ | Brazil | 13/03/2007 | this study | Illumina HiSeq |
| 16933_5#58 | ERR1045686 | BRA_RJ_17 | NA | Rio de Janeiro | RJ | Brazil | 07/02/2007 | this study | Illumina HiSeq |
| 16933_5#57 | ERR1045685 | BRA_RJ_16 | NA | Rio de Janeiro | RJ | Brazil | 19/01/2007 | this study | Illumina HiSeq |
| 16933_5#56 | ERR1045684 | BRA_RJ_15 | NA | Rio de Janeiro | RJ | Brazil | 22/02/2007 | this study | Illumina HiSeq |
| 16933_5#55 | ERR1045683 | BRA_RJ_14 | NA | Rio de Janeiro | RJ | Brazil | 08/03/2007 | this study | Illumina HiSeq |
| 16933_5#54 | ERR1045682 | BRA_RJ_13 | NA | Rio de Janeiro | RJ | Brazil | 06/03/2007 | this study | Illumina HiSeq |
| 16933_5#53 | ERR1045681 | BRA_RJ_12 | NA | Rio de Janeiro | RJ | Brazil | 14/03/2007 | this study | Illumina HiSeq |
| 16933_5#52 | ERR1045680 | BRA_RJ_11 | NA | Rio de Janeiro | RJ | Brazil | 19/03/2007 | this study | Illumina HiSeq |
| 16933_5#51 | ERR1045679 | BRA_RJ_09 | NA | Rio de Janeiro | RJ | Brazil | 12/04/2007 | this study | Illumina HiSeq |
| 16933_5#50 | ERR1045678 | BRA_RJ_08‡ | NA | Rio de Janeiro | RJ | Brazil | 28/09/2006 | this study | Illumina HiSeq |
| 16933_5#5 | ERR1045633 | BRA_PA_47 | NA | Belém | PA | Brazil | 30/11/2004 | this study | Illumina HiSeq |
| 16933_5#49 | ERR1045677 | BRA_RJ_07 | NA | Rio de Janeiro | RJ | Brazil | 20/09/2006 | this study | Illumina HiSeq |
| 16933_5#48 | ERR1045676 | BRA_RJ_06 | NA | Rio de Janeiro | RJ | Brazil | 30/08/2006 | this study | Illumina HiSeq |
| 16933_5#47 | ERR1045675 | BRA_RJ_05 | NA | Rio de Janeiro | RJ | Brazil | 11/10/2006 | this study | Illumina HiSeq |
| 16933_5#46 | ERR1045674 | BRA_RJ_04 | NA | Rio de Janeiro | RJ | Brazil | 29/09/2006 | this study | Illumina HiSeq |
| 16933_5#45 | ERR1045673 | BRA_RJ_02 | NA | Rio de Janeiro | RJ | Brazil | 29/08/2006 | this study | Illumina HiSeq |
| 16933_5#44 | ERR1045672 | BRA_RJ_01 | NA | Rio de Janeiro | RJ | Brazil | 23/08/2006 | this study | Illumina HiSeq |
| 16933_5#43 | ERR1045671 | BRA_PR_30 | NA | Curitiba | PR | Brazil | 21/12/2007 | this study | Illumina HiSeq |
| 16933_5#42 | ERR1045670 | BRA_PR_29 | NA | Curitiba | PR | Brazil | 19/11/2007 | this study | Illumina HiSeq |
| 16933_5#41 | ERR1045669 | BRA_PR_28 | NA | Curitiba | PR | Brazil | 05/01/2008 | this study | Illumina HiSeq |
| 16933_5#40 | ERR1045668 | BRA_PR_27 | NA | Curitiba | PR | Brazil | 19/12/2007 | this study | Illumina HiSeq |
| 16933_5#4 | ERR1045632 | BRA_PA_46 | NA | Belém | PA | Brazil | 30/11/2004 | this study | Illumina HiSeq |
| 16933_5#39 | ERR1045667 | BRA_PR_26 | NA | Curitiba | PR | Brazil | 26/12/2007 | this study | Illumina HiSeq |
| 16933_5#38 | ERR1045666 | BRA_PR_25 | NA | Curitiba | PR | Brazil | 17/10/2007 | this study | Illumina HiSeq |

| | | | | | | | | | |
|---|---|---|---|---|---|---|---|---|---|
| 16933_5#37 | ERR1045665 | BRA_PR_24 | NA | Curitiba | PR | Brazil | 11/01/2008 | this study | Illumina HiSeq |
| 16933_5#36 | ERR1045664 | BRA_PR_23 | NA | Curitiba | PR | Brazil | 09/01/2008 | this study | Illumina HiSeq |
| 16933_5#35 | ERR1045663 | BRA_PR_22 | NA | Curitiba | PR | Brazil | 22/12/2007 | this study | Illumina HiSeq |
| 16933_5#34 | ERR1045662 | BRA_PR_21 | NA | Curitiba | PR | Brazil | 04/12/2007 | this study | Illumina HiSeq |
| 16933_5#33 | ERR1045661 | BRA_PR_20 | NA | Curitiba | PR | Brazil | 17/12/2007 | this study | Illumina HiSeq |
| 16933_5#32 | ERR1045660 | BRA_PR_19 | NA | Curitiba | PR | Brazil | 31/01/2008 | this study | Illumina HiSeq |
| 16933_5#31 | ERR1045659 | BRA_PR_18 | NA | Curitiba | PR | Brazil | 10/01/2008 | this study | Illumina HiSeq |
| 16933_5#30 | ERR1045658 | BRA_PR_17 | NA | Curitiba | PR | Brazil | 26/12/2007 | this study | Illumina HiSeq |
| 16933_5#3 | ERR1045631 | BRA_PA_45 | NA | Belém | PA | Brazil | 26/11/2005 | this study | Illumina HiSeq |
| 16933_5#29 | ERR1045657 | BRA_PR_16 | NA | Curitiba | PR | Brazil | 21/01/2008 | this study | Illumina HiSeq |
| 16933_5#28 | ERR1045656 | BRA_PR_15 | NA | Curitiba | PR | Brazil | 21/01/2008 | this study | Illumina HiSeq |
| 16933_5#27 | ERR1045655 | BRA_PR_14 | NA | Curitiba | PR | Brazil | 23/01/2008 | this study | Illumina HiSeq |
| 16933_5#26 | ERR1045654 | BRA_PR_13 | NA | Curitiba | PR | Brazil | 14/01/2008 | this study | Illumina HiSeq |
| 16933_5#25 | ERR1045653 | BRA_PR_12 | NA | Curitiba | PR | Brazil | 03/01/2008 | this study | Illumina HiSeq |
| 16933_5#24 | ERR1045652 | BRA_PR_11 | NA | Curitiba | PR | Brazil | 05/01/2008 | this study | Illumina HiSeq |
| 16933_5#23 | ERR1045651 | BRA_PR_10 | NA | Curitiba | PR | Brazil | 26/01/2008 | this study | Illumina HiSeq |
| 16933_5#22 | ERR1045650 | BRA_PR_09 | NA | Curitiba | PR | Brazil | 14/01/2008 | this study | Illumina HiSeq |
| 16933_5#21 | ERR1045649 | BRA_PR_08 | NA | Curitiba | PR | Brazil | 23/12/2007 | this study | Illumina HiSeq |
| 16933_5#20 | ERR1045648 | BRA_PR_07 | NA | Curitiba | PR | Brazil | 20/12/2007 | this study | Illumina HiSeq |
| 16933_5#2 | ERR1045630 | BRA_PA_44 | NA | Belém | PA | Brazil | 23/11/2004 | this study | Illumina HiSeq |
| 16933_5#19 | ERR1045647 | BRA_PR_06 | NA | Curitiba | PR | Brazil | 17/03/2008 | this study | Illumina HiSeq |
| 16933_5#18 | ERR1045646 | BRA_PR_05 | NA | Curitiba | PR | Brazil | 20/12/2007 | this study | Illumina HiSeq |
| 16933_5#17 | ERR1045645 | BRA_PR_04 | NA | Curitiba | PR | Brazil | 08/03/2008 | this study | Illumina HiSeq |
| 16933_5#16 | ERR1045644 | BRA_PR_03 | NA | Curitiba | PR | Brazil | 03/01/2008 | this study | Illumina HiSeq |
| 16933_5#15 | ERR1045643 | BRA_PR_02 | NA | Curitiba | PR | Brazil | 21/01/2008 | this study | Illumina HiSeq |

| | | | | | | | | | |
|---|---|---|---|---|---|---|---|---|---|
| 16933_5#14 | ERR1045642 | BRA_PR_01 | NA | Curitiba | PR | Brazil | 19/12/2007 | this study | Illumina HiSeq |
| 16933_5#13 | ERR1045641 | BRA_PA_55 | NA | Belém | PA | Brazil | 21/12/2004 | this study | Illumina HiSeq |
| 16933_5#12 | ERR1045640 | BRA_PA_54 | NA | Belém | PA | Brazil | 20/12/2004 | this study | Illumina HiSeq |
| 16933_5#11 | ERR1045639 | BRA_PA_53 | NA | Belém | PA | Brazil | 17/12/2004 | this study | Illumina HiSeq |
| 16933_5#10 | ERR1045638 | BRA_PA_52 | NA | Belém | PA | Brazil | 16/12/2004 | this study | Illumina HiSeq |
| 16933_5#1 | ERR1045629 | BRA_PA_42 | NA | Belém | PA | Brazil | 22/11/2004 | this study | Illumina HiSeq |
| 16933_4#95 | ERR1045628 | BRA_PA_41 | NA | Belém | PA | Brazil | 18/11/2004 | this study | Illumina HiSeq |
| 16933_4#94 | ERR1045627 | BRA_PA_40 | NA | Belém | PA | Brazil | 16/11/2004 | this study | Illumina HiSeq |
| 16933_4#93 | ERR1045626 | BRA_PA_39 | NA | Belém | PA | Brazil | 12/11/2004 | this study | Illumina HiSeq |
| 16933_4#92 | ERR1045625 | BRA_PA_38 | NA | Belém | PA | Brazil | 12/11/2004 | this study | Illumina HiSeq |
| 16933_4#91 | ERR1045624 | BRA_PA_37 | NA | Belém | PA | Brazil | 09/11/2004 | this study | Illumina HiSeq |
| 16933_4#90 | ERR1045623 | BRA_PA_36 | NA | Belém | PA | Brazil | 05/11/2004 | this study | Illumina HiSeq |
| 16933_4#9 | ERR1045542 | BRA_AM_03 | NA | Manaus | AM | Brazil | 18/08/2010 | this study | Illumina HiSeq |
| 16933_4#89 | ERR1045622 | BRA_PA_35 | NA | Belém | PA | Brazil | 05/11/2004 | this study | Illumina HiSeq |
| 16933_4#88 | ERR1045621 | BRA_PA_34 | NA | Belém | PA | Brazil | 04/11/2004 | this study | Illumina HiSeq |
| 16933_4#87 | ERR1045620 | BRA_PA_33 | NA | Belém | PA | Brazil | 04/11/2004 | this study | Illumina HiSeq |
| 16933_4#86 | ERR1045619 | BRA_PA_32 | NA | Belém | PA | Brazil | 29/10/2004 | this study | Illumina HiSeq |
| 16933_4#85 | ERR1045618 | BRA_PA_31 | NA | Belém | PA | Brazil | 29/10/2004 | this study | Illumina HiSeq |
| 16933_4#84 | ERR1045617 | BRA_PA_30 | NA | Belém | PA | Brazil | 26/10/2004 | this study | Illumina HiSeq |
| 16933_4#83 | ERR1045616 | BRA_PA_29 | NA | Belém | PA | Brazil | 25/10/2004 | this study | Illumina HiSeq |
| 16933_4#82 | ERR1045615 | BRA_PA_28 | NA | Belém | PA | Brazil | 21/10/2004 | this study | Illumina HiSeq |
| 16933_4#81 | ERR1045614 | BRA_PA_27 | NA | Belém | PA | Brazil | NA | this study | Illumina HiSeq |
| 16933_4#80 | ERR1045613 | BRA_PA_26 | NA | Belém | PA | Brazil | 13/06/2005 | this study | Illumina HiSeq |
| 16933_4#8 | ERR1045541 | BRA_AM_02 | NA | Manaus | AM | Brazil | 18/08/2010 | this study | Illumina HiSeq |
| 16933_4#79 | ERR1045612 | BRA_PA_25 | NA | Belém | PA | Brazil | 31/05/2005 | this study | Illumina HiSeq |

| | | | | | | | | | |
|---|---|---|---|---|---|---|---|---|---|
| 16933_4#78 | ERR1045611 | BRA_PA_24 | NA | Marituba | PA | Brazil | 30/05/2005 | this study | Illumina HiSeq |
| 16933_4#77 | ERR1045610 | BRA_PA_23 | NA | Belém | PA | Brazil | 14/09/2004 | this study | Illumina HiSeq |
| 16933_4#76 | ERR1045609 | BRA_PA_22 | NA | Marituba | PA | Brazil | 23/05/2005 | this study | Illumina HiSeq |
| 16933_4#75 | ERR1045608 | BRA_PA_21 | NA | Belém | PA | Brazil | NA | this study | Illumina HiSeq |
| 16933_4#74 | ERR1045607 | BRA_PA_20 | NA | Belém | PA | Brazil | 02/05/2005 | this study | Illumina HiSeq |
| 16933_4#73 | ERR1045606 | BRA_PA_19 | NA | Belém | PA | Brazil | 19/04/2005 | this study | Illumina HiSeq |
| 16933_4#72 | ERR1045605 | BRA_PA_18 | NA | Belém | PA | Brazil | 18/04/2005 | this study | Illumina HiSeq |
| 16933_4#71 | ERR1045604 | BRA_PA_17‡ | NA | Belém | PA | Brazil | 18/04/2005 | this study | Illumina HiSeq |
| 16933_4#70 | ERR1045603 | BRA_PA_16 | NA | Belém | PA | Brazil | 14/04/2005 | this study | Illumina HiSeq |
| 16933_4#7 | ERR1045540 | BRA_AM_01 | NA | Manaus | AM | Brazil | 18/08/2010 | this study | Illumina HiSeq |
| 16933_4#69 | ERR1045602 | BRA_PA_15 | NA | Belém | PA | Brazil | 13/04/2005 | this study | Illumina HiSeq |
| 16933_4#68 | ERR1045601 | BRA_PA_14 | NA | Belém | PA | Brazil | 05/04/2005 | this study | Illumina HiSeq |
| 16933_4#67 | ERR1045600 | BRA_PA_13 | NA | Belém | PA | Brazil | 01/04/2005 | this study | Illumina HiSeq |
| 16933_4#66 | ERR1045599 | BRA_PA_12 | NA | Belém | PA | Brazil | 29/03/2005 | this study | Illumina HiSeq |
| 16933_4#65 | ERR1045598 | BRA_PA_11 | NA | Belém | PA | Brazil | 28/03/2005 | this study | Illumina HiSeq |
| 16933_4#64 | ERR1045597 | BRA_PA_10 | NA | Belém | PA | Brazil | 16/03/2005 | this study | Illumina HiSeq |
| 16933_4#63 | ERR1045596 | BRA_PA_09 | NA | Belém | PA | Brazil | 14/03/2005 | this study | Illumina HiSeq |
| 16933_4#62 | ERR1045595 | BRA_PA_08 | NA | Belém | PA | Brazil | 11/03/2005 | this study | Illumina HiSeq |
| 16933_4#61 | ERR1045594 | BRA_PA_07 | NA | Belém | PA | Brazil | 03/03/2005 | this study | Illumina HiSeq |
| 16933_4#60 | ERR1045593 | BRA_PA_06 | NA | Belém | PA | Brazil | 18/02/2005 | this study | Illumina HiSeq |
| 16933_4#6 | ERR1045539 | BRA_RS1_04 | NA | Santo Ãngelo | RS | Brazil | 14/06/2007 | this study | Illumina HiSeq |
| 16933_4#59 | ERR1045592 | BRA_PA_05 | NA | Belém | PA | Brazil | 26/01/2005 | this study | Illumina HiSeq |
| 16933_4#58 | ERR1045591 | BRA_PA_04 | NA | Belém | PA | Brazil | 17/01/2005 | this study | Illumina HiSeq |
| 16933_4#57 | ERR1045590 | BRA_PA_03 | NA | Belém | PA | Brazil | 10/01/2005 | this study | Illumina HiSeq |
| 16933_4#56 | ERR1045589 | BRA_PA_02 | NA | Belém | PA | Brazil | 07/01/2005 | this study | Illumina HiSeq |

| | | | | | | | | | |
|---|---|---|---|---|---|---|---|---|---|
| 16933_4#55 | ERR1045588 | BRA_PA_01 | NA | Belém | PA | Brazil | 05/01/2005 | this study | Illumina HiSeq |
| 16933_4#54 | ERR1045587 | BRA_MT_03 | NA | Cuiabá | MT | Brazil | 23/04/2007 | this study | Illumina HiSeq |
| 16933_4#53 | ERR1045586 | BRA_MT_02 | NA | Cuiabá | MT | Brazil | 29/11/2006 | this study | Illumina HiSeq |
| 16933_4#52 | ERR1045585 | BRA_MT_01 | NA | Cuiabá | MT | Brazil | 17/10/2006 | this study | Illumina HiSeq |
| 16933_4#51 | ERR1045584 | BRA_GO_18 | NA | Goiânia | GO | Brazil | Nov-06 | this study | Illumina HiSeq |
| 16933_4#50 | ERR1045583 | BRA_GO_17 | NA | Goiânia | GO | Brazil | Jul-06 | this study | Illumina HiSeq |
| 16933_4#5 | ERR1045538 | BRA_RJ_19 | NA | Rio de Janeiro | RJ | Brazil | 02/02/2007 | this study | Illumina HiSeq |
| 16933_4#49 | ERR1045582 | BRA_GO_16 | NA | Goiânia | GO | Brazil | Jun-06 | this study | Illumina HiSeq |
| 16933_4#48 | ERR1045581 | BRA_GO_15 | NA | Goiânia | GO | Brazil | Jul-06 | this study | Illumina HiSeq |
| 16933_4#47 | ERR1045580 | BRA_GO_14 | NA | Goiânia | GO | Brazil | Jun-06 | this study | Illumina HiSeq |
| 16933_4#46 | ERR1045579 | BRA_GO_13 | NA | Goiânia | GO | Brazil | Jun-06 | this study | Illumina HiSeq |
| 16933_4#45 | ERR1045578 | BRA_GO_12 | NA | Goiânia | GO | Brazil | May-06 | this study | Illumina HiSeq |
| 16933_4#44 | ERR1045577 | BRA_GO_11 | NA | Goiânia | GO | Brazil | Jun-06 | this study | Illumina HiSeq |
| 16933_4#43 | ERR1045576 | BRA_GO_10 | NA | Goiânia | GO | Brazil | May-06 | this study | Illumina HiSeq |
| 16933_4#42 | ERR1045575 | BRA_GO_09 | NA | Goiânia | GO | Brazil | Aug-06 | this study | Illumina HiSeq |
| 16933_4#41 | ERR1045574 | BRA_GO_08 | NA | Goiânia | GO | Brazil | Jul-06 | this study | Illumina HiSeq |
| 16933_4#40 | ERR1045573 | BRA_GO_07 | NA | Goiânia | GO | Brazil | Jul-06 | this study | Illumina HiSeq |
| 16933_4#4 | ERR1045537 | BRA_RJ_10 | NA | Rio de Janeiro | RJ | Brazil | 27/03/2007 | this study | Illumina HiSeq |
| 16933_4#39 | ERR1045572 | BRA_GO_06‡ | NA | Goiânia | GO | Brazil | Jul-06 | this study | Illumina HiSeq |
| 16933_4#38 | ERR1045571 | BRA_GO_05 | NA | Goiânia | GO | Brazil | Jul-06 | this study | Illumina HiSeq |
| 16933_4#37 | ERR1045570 | BRA_GO_04 | NA | Goiânia | GO | Brazil | May-06 | this study | Illumina HiSeq |
| 16933_4#36 | ERR1045569 | BRA_GO_03 | NA | Goiânia | GO | Brazil | Jun-06 | this study | Illumina HiSeq |
| 16933_4#35 | ERR1045568 | BRA_GO_02 | NA | Goiânia | GO | Brazil | Aug-06 | this study | Illumina HiSeq |
| 16933_4#34 | ERR1045567 | BRA_GO_01 | NA | Goiânia | GO | Brazil | May-07 | this study | Illumina HiSeq |
| 16933_4#33 | ERR1045566 | BRA_ES_22 | NA | Vitória | ES | Brazil | NA | this study | Illumina HiSeq |

| | | | | | | | | | |
|---|---|---|---|---|---|---|---|---|---|
| 16933_4#32 | ERR1045565 | BRA_ES_21 | NA | Vila Velha | ES | Brazil | 09/08/2007 | this study | Illumina HiSeq |
| 16933_4#31 | ERR1045564 | BRA_ES_20 | NA | Vitória | ES | Brazil | 20/07/2007 | this study | Illumina HiSeq |
| 16933_4#30 | ERR1045563 | BRA_ES_19 | NA | Cariacica | ES | Brazil | 03/09/2007 | this study | Illumina HiSeq |
| 16933_4#3 | ERR1045536 | BRA_RJ_03 | NA | Rio de Janeiro | RJ | Brazil | 19/09/2006 | this study | Illumina HiSeq |
| 16933_4#29 | ERR1045562 | BRA_ES_18 | NA | Vitória | ES | Brazil | 01/09/2007 | this study | Illumina HiSeq |
| 16933_4#28 | ERR1045561 | BRA_ES_17 | NA | Vila Velha | ES | Brazil | 01/09/2007 | this study | Illumina HiSeq |
| 16933_4#27 | ERR1045560 | BRA_ES_16 | NA | Cariacica | ES | Brazil | 30/08/2007 | this study | Illumina HiSeq |
| 16933_4#26 | ERR1045559 | BRA_ES_15 | NA | Vitória | ES | Brazil | 29/08/2007 | this study | Illumina HiSeq |
| 16933_4#25 | ERR1045558 | BRA_ES_14 | NA | Vila Velha | ES | Brazil | 30/08/2007 | this study | Illumina HiSeq |
| 16933_4#24 | ERR1045557 | BRA_ES_13 | NA | Serra | ES | Brazil | 28/08/2007 | this study | Illumina HiSeq |
| 16933_4#23 | ERR1045556 | BRA_ES_12 | NA | Serra | ES | Brazil | 24/08/2007 | this study | Illumina HiSeq |
| 16933_4#22 | ERR1045555 | BRA_ES_11 | NA | Cariacica | ES | Brazil | 24/08/2007 | this study | Illumina HiSeq |
| 16933_4#21 | ERR1045554 | BRA_ES_10 | NA | Serra | ES | Brazil | 23/08/2007 | this study | Illumina HiSeq |
| 16933_4#20 | ERR1045553 | BRA_ES_09 | NA | Cariacica | ES | Brazil | 20/08/2007 | this study | Illumina HiSeq |
| 16933_4#2 | ERR1045535 | BRA_PA_43 | NA | Belém | PA | Brazil | 23/11/2004 | this study | Illumina HiSeq |
| 16933_4#19 | ERR1045552 | BRA_ES_08 | NA | Cariacica | ES | Brazil | 18/08/2007 | this study | Illumina HiSeq |
| 16933_4#18 | ERR1045551 | BRA_ES_07 | NA | Cariacica | ES | Brazil | 17/08/2007 | this study | Illumina HiSeq |
| 16933_4#17 | ERR1045550 | BRA_ES_06 | NA | Vila Velha | ES | Brazil | 10/08/2007 | this study | Illumina HiSeq |
| 16933_4#16 | ERR1045549 | BRA_ES_05 | NA | Cariacica | ES | Brazil | 10/08/2007 | this study | Illumina HiSeq |
| 16933_4#15 | ERR1045548 | BRA_ES_04 | NA | Vitória | ES | Brazil | 06/08/2007 | this study | Illumina HiSeq |
| 16933_4#14 | ERR1045547 | BRA_ES_03 | NA | Cariacica | ES | Brazil | 03/08/2007 | this study | Illumina HiSeq |
| 16933_4#13 | ERR1045546 | BRA_ES_01 | NA | Cariacica | ES | Brazil | 27/07/2007 | this study | Illumina HiSeq |
| 16933_4#12 | ERR1045545 | BRA_AM_06 | NA | Manaus | AM | Brazil | NA | this study | Illumina HiSeq |
| 16933_4#11 | ERR1045544 | BRA_AM_05 | NA | Manaus | AM | Brazil | NA | this study | Illumina HiSeq |
| 16933_4#10 | ERR1045543 | BRA_AM_04 | NA | Manaus | AM | Brazil | NA | this study | Illumina HiSeq |

| | | | | | | | | | |
|---|---|---|---|---|---|---|---|---|---|
| 16933_4#1 | ERR1045534 | BRA_ES_02 | NA | Cariacica | ES | Brazil | 02/08/2007 | this study | Illumina HiSeq |
| 12163_2#95 | ERR494978 | SWE1139 | NA | NA | NA | Sweden | NA | Bryant et al., (2016) Manuscript in preperation | illumina HiSeq |
| 12163_2#94 | ERR494977 | SWE1138 | NA | NA | NA | Sweden | NA | Bryant et al., (2016) Manuscript in preperation | illumina HiSeq |
| 12163_2#93 | ERR494976 | SWE1137 | NA | NA | NA | Sweden | NA | Bryant et al., (2016) Manuscript in preperation | illumina HiSeq |
| 12163_2#90 | ERR494973 | BIR1133 | NA | NA | NA | UK | 2012 | Bryant et al., (2016) Manuscript in preperation | illumina HiSeq |
| 12163_2#87 | ERR494970 | BIR1129 | NA | NA | NA | UK | 2012 | Bryant et al., (2016) Manuscript in preperation | illumina HiSeq |
| 12163_2#86 | ERR494969 | BIR1128 | NA | NA | NA | UK | 2012 | Bryant et al., (2016) Manuscript in preperation | illumina HiSeq |
| 12163_2#84 | ERR494967 | BIR1125 | NA | NA | NA | UK | 2011 | Bryant et al., (2016) Manuscript in preperation | illumina HiSeq |
| 12163_2#76 | ERR494959 | AUS1116 | NA | NA | NA | Australia | 2007 | Bryant et al., (2016) Manuscript in preperation | illumina HiSeq |
| 12163_2#72 | ERR494955 | AUS1107 | NA | NA | NA | Australia | NA | Bryant et al., (2016) Manuscript in preperation | illumina HiSeq |
| 12163_2#71 | ERR494954 | AUS1105 | NA | NA | NA | Australia | NA | Bryant et al., (2016) Manuscript in preperation | illumina HiSeq |
| 12163_2#70 | ERR494953 | AUS1103 | NA | NA | NA | Australia | NA | Bryant et al., (2016) Manuscript in preperation | illumina HiSeq |
| 12163_2#7 | ERR494893 | BIR1034 | NA | NA | NA | UK | 2012 | Bryant et al., (2016) Manuscript in preperation | illumina HiSeq |
| 12163_2#68 | ERR494951 | AUS1099 | NA | NA | NA | Australia | NA | Bryant et al., (2016) Manuscript in preperation | illumina HiSeq |
| 12163_2#67 | ERR494950 | AUS1098 | NA | NA | NA | Australia | NA | Bryant et al., (2016) Manuscript in preperation | illumina HiSeq |
| 12163_2#66 | ERR494949 | AUS1097 | NA | NA | NA | Australia | 2013 | Bryant et al., (2016) Manuscript in preperation | illumina HiSeq |
| 12163_2#64 | ERR494947 | AUS1095 | NA | NA | NA | Australia | 2010 | Bryant et al., (2016) Manuscript in preperation | illumina HiSeq |
| 12163_2#59 | ERR494942 | AUS1090 | NA | NA | NA | Australia | 2010 | Bryant et al., (2016) Manuscript in preperation | illumina HiSeq |
| 12163_2#58 | ERR494941 | RVI1086 | NA | NA | NA | UK | 2013 | Bryant et al., (2016) Manuscript in preperation | illumina HiSeq |
| 12163_2#57 | ERR494940 | BIR1085 | NA | NA | NA | UK | 2012 | Bryant et al., (2016) Manuscript in preperation | illumina HiSeq |
| 12163_2#54 | ERR494937 | BIR1082 | NA | NA | NA | UK | 2012 | Bryant et al., (2016) Manuscript in preperation | illumina HiSeq |
| 12163_2#53 | ERR494936 | BIR1080 | NA | NA | NA | UK | 2012 | Bryant et al., (2016) Manuscript in preperation | illumina HiSeq |

| | | | | | | | | | |
|---|---|---|---|---|---|---|---|---|---|
| 12163_2#51 | ERR494934 | BIR1078 | NA | NA | NA | UK | 2012 | Bryant et al., (2016) Manuscript in preperation | illumina HiSeq |
| 12163_2#49 | ERR494932 | BIR1076 | NA | NA | NA | UK | 2012 | Bryant et al., (2016) Manuscript in preperation | illumina HiSeq |
| 12163_2#48 | ERR494931 | BIR1075 | NA | NA | NA | UK | 2012 | Bryant et al., (2016) Manuscript in preperation | illumina HiSeq |
| 12163_2#47 | ERR494930 | BIR1074 | NA | NA | NA | UK | 2012 | Bryant et al., (2016) Manuscript in preperation | illumina HiSeq |
| 12163_2#45 | ERR494928 | BIR1072 | NA | NA | NA | UK | 2012 | Bryant et al., (2016) Manuscript in preperation | illumina HiSeq |
| 12163_2#41 | ERR494925 | BIR1068 | NA | NA | NA | UK | 2012 | Bryant et al., (2016) Manuscript in preperation | illumina HiSeq |
| 12163_2#4 | ERR494890 | BIR1031 | NA | NA | NA | UK | 2012 | Bryant et al., (2016) Manuscript in preperation | illumina HiSeq |
| 12163_2#37 | ERR494921 | BIR1064 | NA | NA | NA | UK | 2012 | Bryant et al., (2016) Manuscript in preperation | illumina HiSeq |
| 12163_2#34 | ERR494918 | BIR1060 | NA | NA | NA | UK | 2012 | Bryant et al., (2016) Manuscript in preperation | illumina HiSeq |
| 12163_2#33 | ERR494917 | BIR1059 | NA | NA | NA | UK | 2012 | Bryant et al., (2016) Manuscript in preperation | illumina HiSeq |
| 12163_2#30 | ERR494915 | BIR1057 | NA | NA | NA | UK | 2012 | Bryant et al., (2016) Manuscript in preperation | illumina HiSeq |
| 12163_2#29 | ERR494914 | BIR1056 | NA | NA | NA | UK | 2012 | Bryant et al., (2016) Manuscript in preperation | illumina HiSeq |
| 12163_2#28 | ERR494913 | BIR1055 | NA | NA | NA | UK | 2012 | Bryant et al., (2016) Manuscript in preperation | illumina HiSeq |
| 12163_2#26 | ERR494911 | BIR1053 | NA | NA | NA | UK | 2012 | Bryant et al., (2016) Manuscript in preperation | illumina HiSeq |
| 12163_2#25 | ERR494910 | BIR1052 | NA | NA | NA | UK | 2012 | Bryant et al., (2016) Manuscript in preperation | illumina HiSeq |
| 12163_2#22 | ERR494907 | BIR1049 | NA | NA | NA | UK | 2012 | Bryant et al., (2016) Manuscript in preperation | illumina HiSeq |
| 12163_2#2 | ERR494888 | BIR1028 | NA | NA | NA | UK | 2012 | Bryant et al., (2016) Manuscript in preperation | illumina HiSeq |
| 12163_2#19 | ERR494904 | BIR1045 | NA | NA | NA | UK | 2012 | Bryant et al., (2016) Manuscript in preperation | illumina HiSeq |
| 12163_2#16 | ERR494901 | BIR1042 | NA | NA | NA | UK | 2012 | Bryant et al., (2016) Manuscript in preperation | illumina HiSeq |
| 12163_2#14 | ERR494899 | BIR1040 | NA | NA | NA | UK | 2012 | Bryant et al., (2016) Manuscript in preperation | illumina HiSeq |
| 12163_2#11 | ERR494896 | BIR1037 | NA | NA | NA | UK | 2012 | Bryant et al., (2016) Manuscript in preperation | illumina HiSeq |
| 12163_2#1 | ERR494887 | BIR1027 | NA | NA | NA | UK | 2012 | Bryant et al., (2016) Manuscript in preperation | illumina HiSeq |

| | | | | | | | | | |
|---|---|---|---|---|---|---|---|---|---|
| 12163_1#96 | ERR494886 | BIR1026 | NA | NA | NA | UK | 2012 | Bryant et al., (2016) Manuscript in preperation | illumina HiSeq |
| 12163_1#91 | ERR494881 | BIR1020 | NA | NA | NA | UK | 2012 | Bryant et al., (2016) Manuscript in preperation | illumina HiSeq |
| 12163_1#9 | ERR494835 | BIR930 | NA | NA | NA | UK | 2009 | Bryant et al., (2016) Manuscript in preperation | illumina HiSeq |
| 12163_1#87 | ERR494877 | BIR1016 | NA | NA | NA | UK | 2011 | Bryant et al., (2016) Manuscript in preperation | illumina HiSeq |
| 12163_1#85 | ERR494875 | BIR1014 | NA | NA | NA | UK | 2011 | Bryant et al., (2016) Manuscript in preperation | illumina HiSeq |
| 12163_1#79 | ERR494869 | BIR1008 | NA | NA | NA | UK | 2011 | Bryant et al., (2016) Manuscript in preperation | illumina HiSeq |
| 12163_1#75 | ERR494865 | BIR1001 | NA | NA | NA | UK | 2011 | Bryant et al., (2016) Manuscript in preperation | illumina HiSeq |
| 12163_1#62 | ERR494852 | BIR987 | NA | NA | NA | UK | 2011 | Bryant et al., (2016) Manuscript in preperation | illumina HiSeq |
| 12163_1#52 | ERR494842 | BIR977 | NA | NA | NA | UK | 2010 | Bryant et al., (2016) Manuscript in preperation | illumina HiSeq |
| 12163_1#51 | ERR494841 | BIR976 | NA | NA | NA | UK | 2010 | Bryant et al., (2016) Manuscript in preperation | illumina HiSeq |
| 12163_1#49 | ERR494839 | BIR974 | NA | NA | NA | UK | 2010 | Bryant et al., (2016) Manuscript in preperation | illumina HiSeq |
| 12163_1#47 | ERR494837 | BIR971 | NA | NA | NA | UK | 2010 | Bryant et al., (2016) Manuscript in preperation | illumina HiSeq |
| 12163_1#46 | ERR494836 | BIR970 | NA | NA | NA | UK | 2010 | Bryant et al., (2016) Manuscript in preperation | illumina HiSeq |
| 12163_1#44 | ERR490703 | BIR968 | NA | NA | NA | UK | 2010 | Bryant et al., (2016) Manuscript in preperation | illumina HiSeq |
| 12163_1#4 | ERR490670 | BIR926 | NA | NA | NA | UK | 2008 | Bryant et al., (2016) Manuscript in preperation | illumina HiSeq |
| 12163_1#39 | ERR490699 | BIR963 | NA | NA | NA | UK | 2010 | Bryant et al., (2016) Manuscript in preperation | illumina HiSeq |
| 12163_1#37 | ERR490697 | BIR961 | NA | NA | NA | UK | 2010 | Bryant et al., (2016) Manuscript in preperation | illumina HiSeq |
| 12163_1#33 | ERR490693 | BIR957 | NA | NA | NA | UK | 2010 | Bryant et al., (2016) Manuscript in preperation | illumina HiSeq |
| 12163_1#30 | ERR490691 | BIR955 | NA | NA | NA | UK | 2010 | Bryant et al., (2016) Manuscript in preperation | illumina HiSeq |
| 12163_1#26 | ERR490687 | BIR948 | NA | NA | NA | UK | 2009 | Bryant et al., (2016) Manuscript in preperation | illumina HiSeq |
| 12082_5#96 | ERR485005 | SMRL1162 | NA | NA | NA | UK | NA | Bryant et al., (2016) Manuscript in preperation | illumina HiSeq |
| 12082_5#95 | ERR485004 | STH1156 | NA | NA | NA | UK | 2013 | Bryant et al., (2016) Manuscript in preperation | illumina HiSeq |

| | | | | | | | | | |
|---|---|---|---|---|---|---|---|---|---|
| 12082_5#94 | ERR485003 | STH1155 | NA | NA | NA | UK | 2013 | Bryant et al., (2016) Manuscript in preperation | illumina HiSeq |
| 12082_5#93 | ERR485002 | STH1153 | NA | NA | NA | UK | 2013 | Bryant et al., (2016) Manuscript in preperation | illumina HiSeq |
| 12082_5#91 | ERR485000 | STH1151 | NA | NA | NA | UK | 2013 | Bryant et al., (2016) Manuscript in preperation | illumina HiSeq |
| 12082_5#87 | ERR484996 | STH1146 | NA | NA | NA | UK | 2013 | Bryant et al., (2016) Manuscript in preperation | illumina HiSeq |
| 12082_5#86 | ERR484995 | RBL1142 | NA | NA | NA | UK | 2012 | Bryant et al., (2016) Manuscript in preperation | illumina HiSeq |
| 12082_5#84 | ERR484993 | KIN1140 | NA | NA | NA | UK | 2013 | Bryant et al., (2016) Manuscript in preperation | illumina HiSeq |
| 12082_5#83 | ERR484992 | SWE1136 | NA | NA | NA | Sweden | NA | Bryant et al., (2016) Manuscript in preperation | illumina HiSeq |
| 12082_5#81 | ERR484990 | BIR1127 | NA | NA | NA | UK | 2012 | Bryant et al., (2016) Manuscript in preperation | illumina HiSeq |
| 12082_5#80 | ERR484989 | RVI1123 | NA | NA | NA | UK | 2013 | Bryant et al., (2016) Manuscript in preperation | illumina HiSeq |
| 12082_5#74 | ERR484983 | AUS1106 | NA | NA | NA | Australia | NA | Bryant et al., (2016) Manuscript in preperation | illumina HiSeq |
| 12082_5#73 | ERR484982 | AUS1100 | NA | NA | NA | Australia | NA | Bryant et al., (2016) Manuscript in preperation | illumina HiSeq |
| 12082_5#70 | ERR484979 | AUS1087 | NA | NA | NA | Australia | 2007 | Bryant et al., (2016) Manuscript in preperation | illumina HiSeq |
| 12082_5#69 | ERR484978 | BIR1070 | NA | NA | NA | UK | 2012 | Bryant et al., (2016) Manuscript in preperation | illumina HiSeq |
| 12082_5#68 | ERR484977 | BIR1063 | NA | NA | NA | UK | 2012 | Bryant et al., (2016) Manuscript in preperation | illumina HiSeq |
| 12082_5#54 | ERR484963 | BIR925 | NA | NA | NA | UK | 2008 | Bryant et al., (2016) Manuscript in preperation | illumina HiSeq |
| 12078_1#71 | ERR473276 | PAP1174 | NA | NA | NA | UK | 2011 | Bryant et al., (2016) Manuscript in preperation | illumina HiSeq |
| 12078_1#70 | ERR473275 | PAP1173 | NA | NA | NA | UK | 2012 | Bryant et al., (2016) Manuscript in preperation | illumina HiSeq |
| 12078_1#67 | ERR473272 | PAP1170 | NA | NA | NA | UK | 2012 | Bryant et al., (2016) Manuscript in preperation | illumina HiSeq |
| 12078_1#65 | ERR473270 | PAP1168 | NA | NA | NA | UK | NA | Bryant et al., (2016) Manuscript in preperation | illumina HiSeq |
| 12078_1#64 | ERR473269 | PAP1167 | NA | NA | NA | UK | NA | Bryant et al., (2016) Manuscript in preperation | illumina HiSeq |
| 12078_1#62 | ERR473267 | PAP1165 | NA | NA | NA | UK | 2012 | Bryant et al., (2016) Manuscript in preperation | illumina HiSeq |
| 12045_8#47 | ERR484961 | STH1159 | NA | NA | NA | UK | 2013 | Bryant et al., (2016) Manuscript in preperation | illumina HiSeq |

| | | | | | | | | | |
|---|---|---|---|---|---|---|---|---|---|
| 12045_8#44 | ERR484958 | BIR1081 | NA | NA | NA | UK | 2012 | Bryant et al., (2016) Manuscript in preperation | illumina HiSeq |
| 12045_8#42 | ERR484957 | SMRL1164 | NA | NA | NA | UK | NA | Bryant et al., (2016) Manuscript in preperation | illumina HiSeq |
| 12045_8#41 | ERR484956 | SMRL1163 | NA | NA | NA | UK | NA | Bryant et al., (2016) Manuscript in preperation | illumina HiSeq |
| 12045_8#40 | ERR484955 | STH1161 | NA | NA | NA | UK | 2013 | Bryant et al., (2016) Manuscript in preperation | illumina HiSeq |
| 12045_8#39 | ERR484954 | STH1160 | NA | NA | NA | UK | 2013 | Bryant et al., (2016) Manuscript in preperation | illumina HiSeq |
| 12045_8#38 | ERR484953 | STH1158 | NA | NA | NA | UK | 2013 | Bryant et al., (2016) Manuscript in preperation | illumina HiSeq |
| 12045_8#36 | ERR484951 | STH1154 | NA | NA | NA | UK | 2013 | Bryant et al., (2016) Manuscript in preperation | illumina HiSeq |
| 12045_8#33 | ERR484948 | RBL1144 | NA | NA | NA | UK | 2013 | Bryant et al., (2016) Manuscript in preperation | illumina HiSeq |
| 11893_6#9 | ERR459927 | AUS842 | NA | NA | NA | Australia | 2013 | Bryant et al., (2016) Manuscript in preperation | illumina HiSeq |
| 11893_6#7 | ERR459926 | AUS840 | NA | NA | NA | Australia | 2006 | Bryant et al., (2016) Manuscript in preperation | illumina HiSeq |
| 11893_6#66 | ERR459982 | BIR911 | NA | NA | NA | UK | 2008 | Bryant et al., (2016) Manuscript in preperation | illumina HiSeq |
| 11893_6#63 | ERR459979 | BIR908 | NA | NA | NA | UK | 2008 | Bryant et al., (2016) Manuscript in preperation | illumina HiSeq |
| 11893_6#60 | ERR459976 | RVI904 | NA | NA | NA | UK | 2013 | Bryant et al., (2016) Manuscript in preperation | illumina HiSeq |
| 11893_6#58 | ERR459974 | SVH901 | NA | NA | NA | Dublin | NA | Bryant et al., (2016) Manuscript in preperation | illumina HiSeq |
| 11893_6#57 | ERR459973 | SVH900 | NA | NA | NA | Dublin | NA | Bryant et al., (2016) Manuscript in preperation | illumina HiSeq |
| 11893_6#56 | ERR459972 | SVH899 | NA | NA | NA | Dublin | NA | Bryant et al., (2016) Manuscript in preperation | illumina HiSeq |
| 11893_6#55 | ERR459971 | SVH898 | NA | NA | NA | Dublin | NA | Bryant et al., (2016) Manuscript in preperation | illumina HiSeq |
| 11893_6#54 | ERR459970 | SVH896 | NA | NA | NA | Dublin | NA | Bryant et al., (2016) Manuscript in preperation | illumina HiSeq |
| 11893_6#53 | ERR459969 | SVH895 | NA | NA | NA | Dublin | NA | Bryant et al., (2016) Manuscript in preperation | illumina HiSeq |
| 11893_6#51 | ERR459967 | SVH892 | NA | NA | NA | Dublin | NA | Bryant et al., (2016) Manuscript in preperation | illumina HiSeq |
| 11893_6#50 | ERR459966 | AUS888 | NA | NA | NA | Australia | 2007 | Bryant et al., (2016) Manuscript in preperation | illumina HiSeq |
| 11893_6#49 | ERR459965 | AUS887 | NA | NA | NA | Australia | 2006 | Bryant et al., (2016) Manuscript in preperation | illumina HiSeq |

| | | | | | | | | | |
|---|---|---|---|---|---|---|---|---|---|
| 11893_6#48 | ERR459964 | AUS886 | NA | NA | NA | Australia | 2009 | Bryant et al., (2016) Manuscript in preperation | illumina HiSeq |
| 11893_6#42 | ERR459959 | AUS881 | NA | NA | NA | Australia | 2007 | Bryant et al., (2016) Manuscript in preperation | illumina HiSeq |
| 11893_6#39 | ERR459956 | AUS878 | NA | NA | NA | Australia | 2011 | Bryant et al., (2016) Manuscript in preperation | illumina HiSeq |
| 11893_6#38 | ERR459955 | AUS875 | NA | NA | NA | Australia | 2011 | Bryant et al., (2016) Manuscript in preperation | illumina HiSeq |
| 11893_6#36 | ERR459953 | AUS872 | NA | NA | NA | Australia | 2012 | Bryant et al., (2016) Manuscript in preperation | illumina HiSeq |
| 11893_6#3 | ERR459922 | AUS836 | NA | NA | NA | Australia | 2012 | Bryant et al., (2016) Manuscript in preperation | illumina HiSeq |
| 11893_6#23 | ERR459941 | AUS857 | NA | NA | NA | Australia | 2011 | Bryant et al., (2016) Manuscript in preperation | illumina HiSeq |
| 11893_6#22 | ERR459940 | AUS856 | NA | NA | NA | Australia | 2011 | Bryant et al., (2016) Manuscript in preperation | illumina HiSeq |
| 11893_6#21 | ERR459939 | AUS855 | NA | NA | NA | Australia | 2011 | Bryant et al., (2016) Manuscript in preperation | illumina HiSeq |
| 11893_6#2 | ERR459921 | AUS835 | NA | NA | NA | Australia | 2013 | Bryant et al., (2016) Manuscript in preperation | illumina HiSeq |
| 11893_6#17 | ERR459935 | AUS850 | NA | NA | NA | Australia | 2012 | Bryant et al., (2016) Manuscript in preperation | illumina HiSeq |
| 11893_6#11 | ERR459929 | AUS844 | NA | NA | NA | Australia | 2013 | Bryant et al., (2016) Manuscript in preperation | illumina HiSeq |
| 11893_6#10 | ERR459928 | AUS843 | NA | NA | NA | Australia | 2013 | Bryant et al., (2016) Manuscript in preperation | illumina HiSeq |
| 11861_8#9 | ERR459847 | HOL786 | NA | NA | NA | Holland | 2012 | Bryant et al., (2016) Manuscript in preperation | illumina HiSeq |
| 11861_8#6 | ERR459845 | HOL783 | NA | NA | NA | Holland | 2012 | Bryant et al., (2016) Manuscript in preperation | illumina HiSeq |
| 11861_8#4 | ERR459843 | AUS765 | NA | NA | NA | Australia | 2009 | Bryant et al., (2016) Manuscript in preperation | illumina HiSeq |
| 11861_8#3 | ERR459842 | AUS745 | NA | NA | NA | Australia | 2011 | Bryant et al., (2016) Manuscript in preperation | illumina HiSeq |
| 11861_8#27 | ERR459865 | SVH897 | NA | NA | NA | Dublin | NA | Bryant et al., (2016) Manuscript in preperation | illumina HiSeq |
| 11861_8#26 | ERR459864 | SVH894 | NA | NA | NA | Dublin | NA | Bryant et al., (2016) Manuscript in preperation | illumina HiSeq |
| 11861_8#20 | ERR459858 | AUS873 | NA | NA | NA | Australia | 2010 | Bryant et al., (2016) Manuscript in preperation | illumina HiSeq |
| 11861_8#18 | ERR459856 | AUS859 | NA | NA | NA | Australia | 2012 | Bryant et al., (2016) Manuscript in preperation | illumina HiSeq |
| 11861_8#15 | ERR459853 | AUS841 | NA | NA | NA | Australia | 2004 | Bryant et al., (2016) Manuscript in preperation | illumina HiSeq |

| | | | | | | | | | |
|---|---|---|---|---|---|---|---|---|---|
| 11861_8#14 | ERR459852 | AUS822 | NA | NA | NA | Australia | 2012 | Bryant et al., (2016) Manuscript in preperation | illumina HiSeq |
| 11861_7#87 | ERR459833 | AUS827 | NA | NA | NA | Australia | 2012 | Bryant et al., (2016) Manuscript in preperation | illumina HiSeq |
| 11861_7#86 | ERR459832 | AUS826 | NA | NA | NA | Australia | 2011 | Bryant et al., (2016) Manuscript in preperation | illumina HiSeq |
| 11861_7#84 | ERR459830 | AUS824 | NA | NA | NA | Australia | 2009 | Bryant et al., (2016) Manuscript in preperation | illumina HiSeq |
| 11861_7#80 | ERR459826 | AUS815 | NA | NA | NA | Australia | 2011 | Bryant et al., (2016) Manuscript in preperation | illumina HiSeq |
| 11861_7#78 | ERR459824 | AUS813 | NA | NA | NA | Australia | 2012 | Bryant et al., (2016) Manuscript in preperation | illumina HiSeq |
| 11861_7#77 | ERR459823 | AUS812 | NA | NA | NA | Australia | 2011 | Bryant et al., (2016) Manuscript in preperation | illumina HiSeq |
| 11861_7#76 | ERR459822 | AUS811 | NA | NA | NA | Australia | 2012 | Bryant et al., (2016) Manuscript in preperation | illumina HiSeq |
| 11861_7#75 | ERR459821 | AUS810 | NA | NA | NA | Australia | 2012 | Bryant et al., (2016) Manuscript in preperation | illumina HiSeq |
| 11861_7#71 | ERR459817 | AUS806 | NA | NA | NA | Australia | 2012 | Bryant et al., (2016) Manuscript in preperation | illumina HiSeq |
| 11861_7#68 | ERR459814 | AUS803 | NA | NA | NA | Australia | 2009 | Bryant et al., (2016) Manuscript in preperation | illumina HiSeq |
| 11861_7#67 | ERR459813 | AUS802 | NA | NA | NA | Australia | 2003 | Bryant et al., (2016) Manuscript in preperation | illumina HiSeq |
| 11861_7#65 | ERR459811 | AUS800 | NA | NA | NA | Australia | 2008 | Bryant et al., (2016) Manuscript in preperation | illumina HiSeq |
| 11861_7#64 | ERR459810 | AUS799 | NA | NA | NA | Australia | 2009 | Bryant et al., (2016) Manuscript in preperation | illumina HiSeq |
| 11861_7#56 | ERR459802 | AUS791 | NA | NA | NA | Australia | 2011 | Bryant et al., (2016) Manuscript in preperation | illumina HiSeq |
| 11861_7#52 | ERR459798 | HOL787 | NA | NA | NA | Holland | NA | Bryant et al., (2016) Manuscript in preperation | illumina HiSeq |
| 11861_7#48 | ERR459794 | HOL779 | NA | NA | NA | Holland | 2010 | Bryant et al., (2016) Manuscript in preperation | illumina HiSeq |
| 11861_7#46 | ERR459792 | AUS777 | NA | NA | NA | Australia | 2006 | Bryant et al., (2016) Manuscript in preperation | illumina HiSeq |
| 11861_7#44 | ERR459790 | AUS775 | NA | NA | NA | Australia | 2009 | Bryant et al., (2016) Manuscript in preperation | illumina HiSeq |
| 11861_7#42 | ERR459789 | AUS774 | NA | NA | NA | Australia | 2002 | Bryant et al., (2016) Manuscript in preperation | illumina HiSeq |
| 11861_7#41 | ERR459788 | AUS773 | NA | NA | NA | Australia | 2006 | Bryant et al., (2016) Manuscript in preperation | illumina HiSeq |
| 11861_7#27 | ERR459775 | AUS759 | NA | NA | NA | Australia | 2011 | Bryant et al., (2016) Manuscript in preperation | illumina HiSeq |

| | | | | | | | | | |
|---|---|---|---|---|---|---|---|---|---|
| 11861_7#26 | ERR459774 | AUS758 | NA | NA | NA | Australia | 2011 | Bryant et al., (2016) Manuscript in preperation | illumina HiSeq |
| 11861_7#25 | ERR459773 | AUS757 | NA | NA | NA | Australia | 2001 | Bryant et al., (2016) Manuscript in preperation | illumina HiSeq |
| 11861_7#24 | ERR459772 | AUS756 | NA | NA | NA | Australia | 2011 | Bryant et al., (2016) Manuscript in preperation | illumina HiSeq |
| 11861_7#23 | ERR459771 | AUS755 | NA | NA | NA | Australia | 2001 | Bryant et al., (2016) Manuscript in preperation | illumina HiSeq |
| 11861_7#22 | ERR459770 | AUS754 | NA | NA | NA | Australia | 2007 | Bryant et al., (2016) Manuscript in preperation | illumina HiSeq |
| 11861_7#12 | ERR459760 | AUS743 | NA | NA | NA | Australia | 2003 | Bryant et al., (2016) Manuscript in preperation | illumina HiSeq |
| 10702_1#96 | ERR374036 | UNC724 | NA | NA | NA | US | NA | Bryant et al., (2016) Manuscript in preperation | illumina HiSeq |
| 10702_1#95 | ERR374035 | UNC717 | NA | NA | NA | US | NA | Bryant et al., (2016) Manuscript in preperation | illumina HiSeq |
| 10702_1#94 | ERR374034 | UNC713 | NA | NA | NA | US | NA | Bryant et al., (2016) Manuscript in preperation | illumina HiSeq |
| 10702_1#93 | ERR374033 | UNC712 | NA | NA | NA | US | NA | Bryant et al., (2016) Manuscript in preperation | illumina HiSeq |
| 10702_1#92 | ERR374032 | UNC711 | NA | NA | NA | US | NA | Bryant et al., (2016) Manuscript in preperation | illumina HiSeq |
| 10702_1#91 | ERR374031 | UNC708 | NA | NA | NA | US | NA | Bryant et al., (2016) Manuscript in preperation | illumina HiSeq |
| 10702_1#90 | ERR374030 | UNC707 | NA | NA | NA | US | NA | Bryant et al., (2016) Manuscript in preperation | illumina HiSeq |
| 10702_1#9 | ERR373950 | UNC576 | NA | NA | NA | US | NA | Bryant et al., (2016) Manuscript in preperation | illumina HiSeq |
| 10702_1#89 | ERR374029 | UNC705 | NA | NA | NA | US | NA | Bryant et al., (2016) Manuscript in preperation | illumina HiSeq |
| 10702_1#88 | ERR374028 | UNC704 | NA | NA | NA | US | NA | Bryant et al., (2016) Manuscript in preperation | illumina HiSeq |
| 10702_1#87 | ERR374027 | UNC702 | NA | NA | NA | US | NA | Bryant et al., (2016) Manuscript in preperation | illumina HiSeq |
| 10702_1#86 | ERR374026 | UNC701 | NA | NA | NA | US | NA | Bryant et al., (2016) Manuscript in preperation | illumina HiSeq |
| 10702_1#85 | ERR374025 | UNC700 | NA | NA | NA | US | NA | Bryant et al., (2016) Manuscript in preperation | illumina HiSeq |
| 10702_1#84 | ERR374024 | UNC698 | NA | NA | NA | US | NA | Bryant et al., (2016) Manuscript in preperation | illumina HiSeq |
| 10702_1#82 | ERR374022 | UNC695 | NA | NA | NA | US | NA | Bryant et al., (2016) Manuscript in preperation | illumina HiSeq |
| 10702_1#81 | ERR374021 | UNC694 | NA | NA | NA | US | NA | Bryant et al., (2016) Manuscript in preperation | illumina HiSeq |

| | | | | | | | | | |
|---|---|---|---|---|---|---|---|---|---|
| 10702_1#80 | ERR374020 | UNC693 | NA | NA | NA | US | NA | Bryant et al., (2016) Manuscript in preperation | illumina HiSeq |
| 10702_1#8 | ERR373949 | UNC574 | NA | NA | NA | US | NA | Bryant et al., (2016) Manuscript in preperation | illumina HiSeq |
| 10702_1#79 | ERR374019 | UNC691 | NA | NA | NA | US | NA | Bryant et al., (2016) Manuscript in preperation | illumina HiSeq |
| 10702_1#78 | ERR374018 | UNC690 | NA | NA | NA | US | NA | Bryant et al., (2016) Manuscript in preperation | illumina HiSeq |
| 10702_1#77 | ERR374017 | UNC689 | NA | NA | NA | US | NA | Bryant et al., (2016) Manuscript in preperation | illumina HiSeq |
| 10702_1#76 | ERR374016 | UNC685 | NA | NA | NA | US | NA | Bryant et al., (2016) Manuscript in preperation | illumina HiSeq |
| 10702_1#74 | ERR374014 | UNC681 | NA | NA | NA | US | NA | Bryant et al., (2016) Manuscript in preperation | illumina HiSeq |
| 10702_1#73 | ERR374013 | UNC680 | NA | NA | NA | US | NA | Bryant et al., (2016) Manuscript in preperation | illumina HiSeq |
| 10702_1#72 | ERR374012 | UNC678 | NA | NA | NA | US | NA | Bryant et al., (2016) Manuscript in preperation | illumina HiSeq |
| 10702_1#71 | ERR374011 | UNC677 | NA | NA | NA | US | NA | Bryant et al., (2016) Manuscript in preperation | illumina HiSeq |
| 10702_1#70 | ERR374010 | UNC673 | NA | NA | NA | US | NA | Bryant et al., (2016) Manuscript in preperation | illumina HiSeq |
| 10702_1#7 | ERR373948 | UNC573 | NA | NA | NA | US | NA | Bryant et al., (2016) Manuscript in preperation | illumina HiSeq |
| 10702_1#68 | ERR374008 | UNC671 | NA | NA | NA | US | NA | Bryant et al., (2016) Manuscript in preperation | illumina HiSeq |
| 10702_1#67 | ERR374007 | UNC666 | NA | NA | NA | US | NA | Bryant et al., (2016) Manuscript in preperation | illumina HiSeq |
| 10702_1#65 | ERR374005 | UNC661 | NA | NA | NA | US | NA | Bryant et al., (2016) Manuscript in preperation | illumina HiSeq |
| 10702_1#64 | ERR374004 | UNC660 | NA | NA | NA | US | NA | Bryant et al., (2016) Manuscript in preperation | illumina HiSeq |
| 10702_1#61 | ERR374001 | UNC655 | NA | NA | NA | US | NA | Bryant et al., (2016) Manuscript in preperation | illumina HiSeq |
| 10702_1#59 | ERR373999 | UNC652 | NA | NA | NA | US | NA | Bryant et al., (2016) Manuscript in preperation | illumina HiSeq |
| 10702_1#57 | ERR373997 | UNC649 | NA | NA | NA | US | NA | Bryant et al., (2016) Manuscript in preperation | illumina HiSeq |
| 10702_1#55 | ERR373995 | UNC645 | NA | NA | NA | US | NA | Bryant et al., (2016) Manuscript in preperation | illumina HiSeq |
| 10702_1#53 | ERR373993 | UNC638 | NA | NA | NA | US | NA | Bryant et al., (2016) Manuscript in preperation | illumina HiSeq |
| 10702_1#52 | ERR373992 | UNC637 | NA | NA | NA | US | NA | Bryant et al., (2016) Manuscript in preperation | illumina HiSeq |

| | | | | | | | | | |
|---|---|---|---|---|---|---|---|---|---|
| 10702_1#51 | ERR373991 | UNC636 | NA | NA | NA | US | NA | Bryant et al., (2016) Manuscript in preperation | illumina HiSeq |
| 10702_1#50 | ERR373990 | UNC634 | NA | NA | NA | US | NA | Bryant et al., (2016) Manuscript in preperation | illumina HiSeq |
| 10702_1#49 | ERR373989 | UNC633 | NA | NA | NA | US | NA | Bryant et al., (2016) Manuscript in preperation | illumina HiSeq |
| 10702_1#48 | ERR373988 | UNC631 | NA | NA | NA | US | NA | Bryant et al., (2016) Manuscript in preperation | illumina HiSeq |
| 10702_1#46 | ERR373986 | UNC629 | NA | NA | NA | US | NA | Bryant et al., (2016) Manuscript in preperation | illumina HiSeq |
| 10702_1#45 | ERR373985 | UNC628 | NA | NA | NA | US | NA | Bryant et al., (2016) Manuscript in preperation | illumina HiSeq |
| 10702_1#44 | ERR373984 | UNC625 | NA | NA | NA | US | NA | Bryant et al., (2016) Manuscript in preperation | illumina HiSeq |
| 10702_1#42 | ERR373983 | UNC622 | NA | NA | NA | US | NA | Bryant et al., (2016) Manuscript in preperation | illumina HiSeq |
| 10702_1#41 | ERR373982 | UNC621 | NA | NA | NA | US | NA | Bryant et al., (2016) Manuscript in preperation | illumina HiSeq |
| 10702_1#40 | ERR373981 | UNC620 | NA | NA | NA | US | NA | Bryant et al., (2016) Manuscript in preperation | illumina HiSeq |
| 10702_1#39 | ERR373980 | UNC619 | NA | NA | NA | US | NA | Bryant et al., (2016) Manuscript in preperation | illumina HiSeq |
| 10702_1#38 | ERR373979 | UNC618 | NA | NA | NA | US | NA | Bryant et al., (2016) Manuscript in preperation | illumina HiSeq |
| 10702_1#36 | ERR373977 | UNC615 | NA | NA | NA | US | NA | Bryant et al., (2016) Manuscript in preperation | illumina HiSeq |
| 10702_1#35 | ERR373976 | UNC613 | NA | NA | NA | US | NA | Bryant et al., (2016) Manuscript in preperation | illumina HiSeq |
| 10702_1#34 | ERR373975 | UNC612 | NA | NA | NA | US | NA | Bryant et al., (2016) Manuscript in preperation | illumina HiSeq |
| 10702_1#33 | ERR373974 | UNC611 | NA | NA | NA | US | NA | Bryant et al., (2016) Manuscript in preperation | illumina HiSeq |
| 10702_1#32 | ERR373973 | UNC607 | NA | NA | NA | US | NA | Bryant et al., (2016) Manuscript in preperation | illumina HiSeq |
| 10702_1#31 | ERR373972 | UNC606 | NA | NA | NA | US | NA | Bryant et al., (2016) Manuscript in preperation | illumina HiSeq |
| 10702_1#30 | ERR373971 | UNC604 | NA | NA | NA | US | NA | Bryant et al., (2016) Manuscript in preperation | illumina HiSeq |
| 10702_1#3 | ERR373944 | DEN541 | NA | NA | NA | Denmark | NA | Bryant et al., (2016) Manuscript in preperation | illumina HiSeq |
| 10702_1#29 | ERR373970 | UNC603 | NA | NA | NA | US | NA | Bryant et al., (2016) Manuscript in preperation | illumina HiSeq |
| 10702_1#25 | ERR373966 | UNC598 | NA | NA | NA | US | NA | Bryant et al., (2016) Manuscript in preperation | illumina HiSeq |

| | | | | | | | | | |
|---|---|---|---|---|---|---|---|---|---|
| 10702_1#24 | ERR373965 | UNC597 | NA | NA | NA | US | NA | Bryant et al., (2016) Manuscript in preperation | illumina HiSeq |
| 10702_1#23 | ERR373964 | UNC596 | NA | NA | NA | US | NA | Bryant et al., (2016) Manuscript in preperation | illumina HiSeq |
| 10702_1#21 | ERR373962 | UNC594 | NA | NA | NA | US | NA | Bryant et al., (2016) Manuscript in preperation | illumina HiSeq |
| 10702_1#20 | ERR373961 | UNC591 | NA | NA | NA | US | NA | Bryant et al., (2016) Manuscript in preperation | illumina HiSeq |
| 10702_1#2 | ERR373943 | DEN540 | NA | NA | NA | Denmark | NA | Bryant et al., (2016) Manuscript in preperation | illumina HiSeq |
| 10702_1#19 | ERR373960 | UNC589 | NA | NA | NA | US | NA | Bryant et al., (2016) Manuscript in preperation | illumina HiSeq |
| 10702_1#18 | ERR373959 | UNC587 | NA | NA | NA | US | NA | Bryant et al., (2016) Manuscript in preperation | illumina HiSeq |
| 10702_1#17 | ERR373958 | UNC586 | NA | NA | NA | US | NA | Bryant et al., (2016) Manuscript in preperation | illumina HiSeq |
| 10702_1#15 | ERR373956 | UNC584 | NA | NA | NA | US | NA | Bryant et al., (2016) Manuscript in preperation | illumina HiSeq |
| 10702_1#14 | ERR373955 | UNC581 | NA | NA | NA | US | NA | Bryant et al., (2016) Manuscript in preperation | illumina HiSeq |
| 10702_1#13 | ERR373954 | UNC580 | NA | NA | NA | US | NA | Bryant et al., (2016) Manuscript in preperation | illumina HiSeq |
| 10702_1#12 | ERR373953 | UNC579 | NA | NA | NA | US | NA | Bryant et al., (2016) Manuscript in preperation | illumina HiSeq |
| 10702_1#11 | ERR373952 | UNC578 | NA | NA | NA | US | NA | Bryant et al., (2016) Manuscript in preperation | illumina HiSeq |
| 10702_1#10 | ERR373951 | UNC577 | NA | NA | NA | US | NA | Bryant et al., (2016) Manuscript in preperation | illumina HiSeq |
| 10702_1#1 | ERR373942 | DEN538 | NA | NA | NA | Denmark | NA | Bryant et al., (2016) Manuscript in preperation | illumina HiSeq |
| 10665_4#9 | ERR369264 | SMRL264 | NA | NA | NA | UK | NA | Bryant et al., (2016) Manuscript in preperation | illumina HiSeq |
| 10665_4#83 | ERR369338 | SMRL338 | NA | NA | NA | UK | NA | Bryant et al., (2016) Manuscript in preperation | illumina HiSeq |
| 10665_4#80 | ERR369335 | SMRL335 | NA | NA | NA | UK | 2007 | Bryant et al., (2016) Manuscript in preperation | illumina HiSeq |
| 10665_4#8 | ERR369263 | SMRL263 | NA | NA | NA | UK | 2005 | Bryant et al., (2016) Manuscript in preperation | illumina HiSeq |
| 10665_4#75 | ERR369330 | SMRL330 | NA | NA | NA | UK | 2007 | Bryant et al., (2016) Manuscript in preperation | illumina HiSeq |
| 10665_4#74 | ERR369329 | SMRL329 | NA | NA | NA | UK | NA | Bryant et al., (2016) Manuscript in preperation | illumina HiSeq |
| 10665_4#71 | ERR369326 | SMRL326 | NA | NA | NA | UK | 2007 | Bryant et al., (2016) Manuscript in preperation | illumina HiSeq |

| | | | | | | | | | |
|---|---|---|---|---|---|---|---|---|---|
| 10665_4#54 | ERR369309 | SMRL309 | NA | NA | NA | UK | 2006 | Bryant et al., (2016) Manuscript in preperation | illumina HiSeq |
| 10665_4#53 | ERR369308 | SMRL308 | NA | NA | NA | UK | 2006 | Bryant et al., (2016) Manuscript in preperation | illumina HiSeq |
| 10665_4#49 | ERR369304 | SMRL304 | NA | NA | NA | UK | 2006 | Bryant et al., (2016) Manuscript in preperation | illumina HiSeq |
| 10665_4#48 | ERR369303 | SMRL303 | NA | NA | NA | UK | 2006 | Bryant et al., (2016) Manuscript in preperation | illumina HiSeq |
| 10665_4#45 | ERR369300 | SMRL300 | NA | NA | NA | UK | 2006 | Bryant et al., (2016) Manuscript in preperation | illumina HiSeq |
| 10665_4#4 | ERR369259 | SMRL259 | NA | NA | NA | UK | NA | Bryant et al., (2016) Manuscript in preperation | illumina HiSeq |
| 10665_4#39 | ERR369294 | SMRL294 | NA | NA | NA | UK | 2006 | Bryant et al., (2016) Manuscript in preperation | illumina HiSeq |
| 10665_4#37 | ERR369292 | SMRL292 | NA | NA | NA | UK | 2006 | Bryant et al., (2016) Manuscript in preperation | illumina HiSeq |
| 10665_4#36 | ERR369291 | SMRL291 | NA | NA | NA | UK | 2006 | Bryant et al., (2016) Manuscript in preperation | illumina HiSeq |
| 10665_4#34 | ERR369289 | SMRL289 | NA | NA | NA | UK | 2006 | Bryant et al., (2016) Manuscript in preperation | illumina HiSeq |
| 10665_4#25 | ERR369280 | SMRL280 | NA | NA | NA | UK | 2005 | Bryant et al., (2016) Manuscript in preperation | illumina HiSeq |
| 10665_4#24 | ERR369279 | SMRL279 | NA | NA | NA | UK | NA | Bryant et al., (2016) Manuscript in preperation | illumina HiSeq |
| 10665_4#23 | ERR369278 | SMRL278 | NA | NA | NA | UK | 2005 | Bryant et al., (2016) Manuscript in preperation | illumina HiSeq |
| 10665_4#22 | ERR369277 | SMRL277 | NA | NA | NA | UK | 2005 | Bryant et al., (2016) Manuscript in preperation | illumina HiSeq |
| 10665_4#20 | ERR369275 | SMRL275 | NA | NA | NA | UK | 2005 | Bryant et al., (2016) Manuscript in preperation | illumina HiSeq |
| 10665_4#10 | ERR369265 | SMRL265 | NA | NA | NA | UK | 2005 | Bryant et al., (2016) Manuscript in preperation | illumina HiSeq |
| 10665_3#8 | ERR369178 | SMRL178 | NA | NA | NA | UK | 2002 | Bryant et al., (2016) Manuscript in preperation | illumina HiSeq |
| 10665_3#78 | ERR369248 | SMRL248 | NA | NA | NA | UK | NA | Bryant et al., (2016) Manuscript in preperation | illumina HiSeq |
| 10665_3#76 | ERR369246 | SMRL246 | NA | NA | NA | UK | 2004 | Bryant et al., (2016) Manuscript in preperation | illumina HiSeq |
| 10665_3#61 | ERR369231 | SMRL231 | NA | NA | NA | UK | 2004 | Bryant et al., (2016) Manuscript in preperation | illumina HiSeq |
| 10665_3#60 | ERR369230 | SMRL230 | NA | NA | NA | UK | 2004 | Bryant et al., (2016) Manuscript in preperation | illumina HiSeq |
| 10665_3#58 | ERR369228 | SMRL228 | NA | NA | NA | UK | 2004 | Bryant et al., (2016) Manuscript in preperation | illumina HiSeq |

| | | | | | | | | | |
|---|---|---|---|---|---|---|---|---|---|
| 10665_3#51 | ERR369221 | SMRL221 | NA | NA | NA | UK | 2004 | Bryant et al., (2016) Manuscript in preperation | illumina HiSeq |
| 10665_3#5 | ERR369175 | SMRL175 | NA | NA | NA | UK | 2001 | Bryant et al., (2016) Manuscript in preperation | illumina HiSeq |
| 10665_3#47 | ERR369217 | SMRL217 | NA | NA | NA | UK | 2003 | Bryant et al., (2016) Manuscript in preperation | illumina HiSeq |
| 10665_3#39 | ERR369209 | SMRL209 | NA | NA | NA | UK | NA | Bryant et al., (2016) Manuscript in preperation | illumina HiSeq |
| 10665_3#28 | ERR369198 | SMRL198 | NA | NA | NA | UK | 2003 | Bryant et al., (2016) Manuscript in preperation | illumina HiSeq |
| 10665_3#27 | ERR369197 | SMRL197 | NA | NA | NA | UK | 2003 | Bryant et al., (2016) Manuscript in preperation | illumina HiSeq |
| 10665_3#26 | ERR369196 | SMRL196 | NA | NA | NA | UK | NA | Bryant et al., (2016) Manuscript in preperation | illumina HiSeq |
| 10665_3#19 | ERR369189 | SMRL189 | NA | NA | NA | UK | 2002 | Bryant et al., (2016) Manuscript in preperation | illumina HiSeq |
| 10665_2#96 | ERR369170 | PAP728 | NA | NA | NA | UK | NA | Bryant et al., (2016) Manuscript in preperation | illumina HiSeq |
| 10665_2#93 | ERR369167 | UNC687 | NA | NA | NA | US | NA | Bryant et al., (2016) Manuscript in preperation | illumina HiSeq |
| 10665_2#91 | ERR369165 | UNC648 | NA | NA | NA | US | NA | Bryant et al., (2016) Manuscript in preperation | illumina HiSeq |
| 10665_2#81 | ERR369155 | RVI551 | NA | NA | NA | UK | 2012 | Bryant et al., (2016) Manuscript in preperation | illumina HiSeq |
| 10665_2#80 | ERR369154 | RVI550 | NA | NA | NA | UK | 2013 | Bryant et al., (2016) Manuscript in preperation | illumina HiSeq |
| 10665_2#79 | ERR369153 | RVI549 | NA | NA | NA | UK | 2013 | Bryant et al., (2016) Manuscript in preperation | illumina HiSeq |
| 10665_2#76 | ERR369150 | DEN537 | NA | NA | NA | Denmark | NA | Bryant et al., (2016) Manuscript in preperation | illumina HiSeq |
| 10665_2#75 | ERR369149 | DEN536 | NA | NA | NA | Denmark | NA | Bryant et al., (2016) Manuscript in preperation | illumina HiSeq |
| 10665_2#74 | ERR369148 | DEN533 | NA | NA | NA | Denmark | NA | Bryant et al., (2016) Manuscript in preperation | illumina HiSeq |
| 10665_2#73 | ERR369147 | DEN532 | NA | NA | NA | Denmark | NA | Bryant et al., (2016) Manuscript in preperation | illumina HiSeq |
| 10665_2#72 | ERR369146 | DEN530 | NA | NA | NA | Denmark | NA | Bryant et al., (2016) Manuscript in preperation | illumina HiSeq |
| 10665_2#71 | ERR369145 | DEN527 | NA | NA | NA | Denmark | NA | Bryant et al., (2016) Manuscript in preperation | illumina HiSeq |
| 10665_2#70 | ERR369144 | DEN524 | NA | NA | NA | Denmark | NA | Bryant et al., (2016) Manuscript in preperation | illumina HiSeq |
| 10665_2#69 | ERR369143 | DEN523 | NA | NA | NA | Denmark | NA | Bryant et al., (2016) Manuscript in preperation | illumina HiSeq |

| | | | | | | | | | |
|---|---|---|---|---|---|---|---|---|---|
| 10665_2#68 | ERR369142 | DEN522 | NA | NA | NA | Denmark | NA | Bryant et al., (2016) Manuscript in preperation | illumina HiSeq |
| 10665_2#67 | ERR369141 | DEN521 | NA | NA | NA | Denmark | NA | Bryant et al., (2016) Manuscript in preperation | illumina HiSeq |
| 10665_2#66 | ERR369140 | DEN520 | NA | NA | NA | Denmark | NA | Bryant et al., (2016) Manuscript in preperation | illumina HiSeq |
| 10665_2#65 | ERR369139 | DEN518 | NA | NA | NA | Denmark | NA | Bryant et al., (2016) Manuscript in preperation | illumina HiSeq |
| 10665_2#64 | ERR369138 | DEN517 | NA | NA | NA | Denmark | NA | Bryant et al., (2016) Manuscript in preperation | illumina HiSeq |
| 10665_2#63 | ERR369137 | DEN516 | NA | NA | NA | Denmark | NA | Bryant et al., (2016) Manuscript in preperation | illumina HiSeq |
| 10665_2#62 | ERR369136 | DEN515 | NA | NA | NA | Denmark | NA | Bryant et al., (2016) Manuscript in preperation | illumina HiSeq |
| 10665_2#61 | ERR369135 | DEN513 | NA | NA | NA | Denmark | NA | Bryant et al., (2016) Manuscript in preperation | illumina HiSeq |
| 10665_2#60 | ERR369134 | DEN511 | NA | NA | NA | Denmark | NA | Bryant et al., (2016) Manuscript in preperation | illumina HiSeq |
| 10665_2#59 | ERR369133 | SMRL510 | NA | NA | NA | UK | 2011 | Bryant et al., (2016) Manuscript in preperation | illumina HiSeq |
| 10660_1#8 | ERR363414 | UNC686 | NA | NA | NA | US | NA | Bryant et al., (2016) Manuscript in preperation | illumina HiSeq |
| 10660_1#7 | ERR363413 | UNC714 | NA | NA | NA | US | NA | Bryant et al., (2016) Manuscript in preperation | illumina HiSeq |
| 10660_1#53 | ERR363459 | DEN539 | NA | NA | NA | Denmark | NA | Bryant et al., (2016) Manuscript in preperation | illumina HiSeq |
| 10660_1#51 | ERR363457 | DEN528 | NA | NA | NA | Denmark | NA | Bryant et al., (2016) Manuscript in preperation | illumina HiSeq |
| 10660_1#5 | ERR363411 | UNC670 | NA | NA | NA | US | NA | Bryant et al., (2016) Manuscript in preperation | illumina HiSeq |
| 10660_1#49 | ERR363455 | DEN512 | NA | NA | NA | Denmark | NA | Bryant et al., (2016) Manuscript in preperation | illumina HiSeq |
| 10660_1#48 | ERR363454 | UNC668 | NA | NA | NA | US | NA | Bryant et al., (2016) Manuscript in preperation | illumina HiSeq |
| 10660_1#45 | ERR363451 | RVI542 | NA | NA | NA | UK | 2013 | Bryant et al., (2016) Manuscript in preperation | illumina HiSeq |
| 10660_1#44 | ERR363450 | DEN534 | NA | NA | NA | Denmark | NA | Bryant et al., (2016) Manuscript in preperation | illumina HiSeq |
| 10660_1#42 | ERR363448 | DEN531 | NA | NA | NA | Denmark | NA | Bryant et al., (2016) Manuscript in preperation | illumina HiSeq |
| 10660_1#4 | ERR363410 | RVI553 | NA | NA | NA | UK | 2013 | Bryant et al., (2016) Manuscript in preperation | illumina HiSeq |
| 10660_1#38 | ERR363444 | UNC626 | NA | NA | NA | US | NA | Bryant et al., (2016) Manuscript in preperation | illumina HiSeq |

| | | | | | | | | | |
|---|---|---|---|---|---|---|---|---|---|
| 10660_1#36 | ERR363442 | DEN526 | NA | NA | NA | Denmark | NA | Bryant et al., (2016) Manuscript in preperation | illumina HiSeq |
| 10660_1#34 | ERR363440 | UNC635 | NA | NA | NA | US | NA | Bryant et al., (2016) Manuscript in preperation | illumina HiSeq |
| 10660_1#33 | ERR363439 | UNC605 | NA | NA | NA | US | NA | Bryant et al., (2016) Manuscript in preperation | illumina HiSeq |
| 10660_1#32 | ERR363438 | DEN514 | NA | NA | NA | Denmark | NA | Bryant et al., (2016) Manuscript in preperation | illumina HiSeq |
| 10660_1#3 | ERR363409 | UNC715 | NA | NA | NA | US | NA | Bryant et al., (2016) Manuscript in preperation | illumina HiSeq |
| 10660_1#28 | ERR363434 | PAP730 | NA | NA | NA | UK | NA | Bryant et al., (2016) Manuscript in preperation | illumina HiSeq |
| 10660_1#27 | ERR363433 | UNC592 | NA | NA | NA | US | NA | Bryant et al., (2016) Manuscript in preperation | illumina HiSeq |
| 10660_1#25 | ERR363431 | UNC643 | NA | NA | NA | US | NA | Bryant et al., (2016) Manuscript in preperation | illumina HiSeq |
| 10660_1#24 | ERR363430 | DEN529 | NA | NA | NA | Denmark | NA | Bryant et al., (2016) Manuscript in preperation | illumina HiSeq |
| 10660_1#23 | ERR363429 | UNC651 | NA | NA | NA | US | NA | Bryant et al., (2016) Manuscript in preperation | illumina HiSeq |
| 10660_1#21 | ERR363427 | UNC688 | NA | NA | NA | US | NA | Bryant et al., (2016) Manuscript in preperation | illumina HiSeq |
| 10660_1#19 | ERR363425 | UNC720 | NA | NA | NA | US | NA | Bryant et al., (2016) Manuscript in preperation | illumina HiSeq |
| 10660_1#18 | ERR363424 | DEN535 | NA | NA | NA | Denmark | NA | Bryant et al., (2016) Manuscript in preperation | illumina HiSeq |
| 10660_1#16 | ERR363422 | DEN519 | NA | NA | NA | Denmark | NA | Bryant et al., (2016) Manuscript in preperation | illumina HiSeq |
| 10660_1#12 | ERR363418 | UNC679 | NA | NA | NA | US | NA | Bryant et al., (2016) Manuscript in preperation | illumina HiSeq |
| 10660_1#11 | ERR363417 | UNC640 | NA | NA | NA | US | NA | Bryant et al., (2016) Manuscript in preperation | illumina HiSeq |
| 10660_1#1 | ERR363407 | UNC706 | NA | NA | NA | US | NA | Bryant et al., (2016) Manuscript in preperation | illumina HiSeq |
| 10625_5#9 | ERR363330 | AHL94 | NA | NA | NA | UK | 2011 | Bryant et al., (2016) Manuscript in preperation | illumina HiSeq |
| 10625_5#82 | ERR363403 | SMRL167 | NA | NA | NA | UK | 2001 | Bryant et al., (2016) Manuscript in preperation | illumina HiSeq |
| 10625_5#8 | ERR363329 | SVH93 | NA | NA | NA | Dublin | NA | Bryant et al., (2016) Manuscript in preperation | illumina HiSeq |
| 10625_5#78 | ERR363399 | SMRL163 | NA | NA | NA | UK | 2001 | Bryant et al., (2016) Manuscript in preperation | illumina HiSeq |
| 10625_5#75 | ERR363396 | SMRL160 | NA | NA | NA | UK | 2001 | Bryant et al., (2016) Manuscript in preperation | illumina HiSeq |

| | | | | | | | | | |
|---|---|---|---|---|---|---|---|---|---|
| 10625_5#74 | ERR363395 | SMRL159 | NA | NA | NA | UK | 2001 | Bryant et al., (2016) Manuscript in preperation | illumina HiSeq |
| 10625_5#73 | ERR363394 | SMRL158 | NA | NA | NA | UK | 2001 | Bryant et al., (2016) Manuscript in preperation | illumina HiSeq |
| 10625_5#71 | ERR363392 | SMRL156 | NA | NA | NA | UK | NA | Bryant et al., (2016) Manuscript in preperation | illumina HiSeq |
| 10625_5#70 | ERR363391 | SMRL155 | NA | NA | NA | UK | 2001 | Bryant et al., (2016) Manuscript in preperation | illumina HiSeq |
| 10625_5#69 | ERR363390 | SMRL154 | NA | NA | NA | UK | 2001 | Bryant et al., (2016) Manuscript in preperation | illumina HiSeq |
| 10625_5#68 | ERR363389 | SMRL153 | NA | NA | NA | UK | 2001 | Bryant et al., (2016) Manuscript in preperation | illumina HiSeq |
| 10625_5#67 | ERR363388 | SMRL152 | NA | NA | NA | UK | NA | Bryant et al., (2016) Manuscript in preperation | illumina HiSeq |
| 10625_5#66 | ERR363387 | SMRL151 | NA | NA | NA | UK | 2001 | Bryant et al., (2016) Manuscript in preperation | illumina HiSeq |
| 10625_5#65 | ERR363386 | SMRL150 | NA | NA | NA | UK | 2001 | Bryant et al., (2016) Manuscript in preperation | illumina HiSeq |
| 10625_5#63 | ERR363384 | SMRL148 | NA | NA | NA | UK | 2001 | Bryant et al., (2016) Manuscript in preperation | illumina HiSeq |
| 10625_5#58 | ERR363379 | BTL143 | NA | NA | NA | UK | 2012 | Bryant et al., (2016) Manuscript in preperation | illumina HiSeq |
| 10625_5#57 | ERR363378 | BTL142 | NA | NA | NA | UK | 2012 | Bryant et al., (2016) Manuscript in preperation | illumina HiSeq |
| 10625_5#56 | ERR363377 | BTL141 | NA | NA | NA | UK | 2012 | Bryant et al., (2016) Manuscript in preperation | illumina HiSeq |
| 10625_5#40 | ERR363361 | JRH125 | NA | NA | NA | UK | 2012 | Bryant et al., (2016) Manuscript in preperation | illumina HiSeq |
| 10625_5#4 | ERR363325 | SVH89 | NA | NA | NA | Dublin | NA | Bryant et al., (2016) Manuscript in preperation | illumina HiSeq |
| 10625_5#39 | ERR363360 | JRH124 | NA | NA | NA | UK | 2012 | Bryant et al., (2016) Manuscript in preperation | illumina HiSeq |
| 10625_5#37 | ERR363358 | JRH122 | NA | NA | NA | UK | 2012 | Bryant et al., (2016) Manuscript in preperation | illumina HiSeq |
| 10625_5#35 | ERR363356 | JRH120 | NA | NA | NA | UK | 2012 | Bryant et al., (2016) Manuscript in preperation | illumina HiSeq |
| 10625_5#34 | ERR363355 | JRH119 | NA | NA | NA | UK | 2012 | Bryant et al., (2016) Manuscript in preperation | illumina HiSeq |
| 10625_5#32 | ERR363353 | JRH117 | NA | NA | NA | UK | 2012 | Bryant et al., (2016) Manuscript in preperation | illumina HiSeq |
| 10625_5#30 | ERR363351 | JRH115 | NA | NA | NA | UK | 2012 | Bryant et al., (2016) Manuscript in preperation | illumina HiSeq |
| 10625_5#3 | ERR363324 | SVH88 | NA | NA | NA | Dublin | NA | Bryant et al., (2016) Manuscript in preperation | illumina HiSeq |

| | | | | | | | | | |
|---|---|---|---|---|---|---|---|---|---|
| 10625_5#29 | ERR363350 | JRH114 | NA | NA | NA | UK | 2012 | Bryant et al., (2016) Manuscript in preperation | illumina HiSeq |
| 10625_5#28 | ERR363349 | JRH113 | NA | NA | NA | UK | 2012 | Bryant et al., (2016) Manuscript in preperation | illumina HiSeq |
| 10625_5#25 | ERR363346 | AHL110 | NA | NA | NA | UK | 2012 | Bryant et al., (2016) Manuscript in preperation | illumina HiSeq |
| 10625_5#24 | ERR363345 | AHL109 | NA | NA | NA | UK | 2012 | Bryant et al., (2016) Manuscript in preperation | illumina HiSeq |
| 10625_5#22 | ERR363343 | AHL107 | NA | NA | NA | UK | 2012 | Bryant et al., (2016) Manuscript in preperation | illumina HiSeq |
| 10625_5#21 | ERR363342 | AHL106 | NA | NA | NA | UK | 2012 | Bryant et al., (2016) Manuscript in preperation | illumina HiSeq |
| 10625_5#20 | ERR363341 | RLB105 | NA | NA | NA | UK | 2011 | Bryant et al., (2016) Manuscript in preperation | illumina HiSeq |
| 10625_5#2 | ERR363323 | SVH87 | NA | NA | NA | Dublin | NA | Bryant et al., (2016) Manuscript in preperation | illumina HiSeq |
| 10625_5#18 | ERR363339 | RLB103 | NA | NA | NA | UK | 2011 | Bryant et al., (2016) Manuscript in preperation | illumina HiSeq |
| 10625_5#13 | ERR363334 | BTL98 | NA | NA | NA | UK | 2012 | Bryant et al., (2016) Manuscript in preperation | illumina HiSeq |
| 10625_5#12 | ERR363333 | BTL97 | NA | NA | NA | UK | 2012 | Bryant et al., (2016) Manuscript in preperation | illumina HiSeq |
| 10625_4#9 | ERR363245 | WRCM9 | NA | NA | NA | UK | NA | Bryant et al., (2016) Manuscript in preperation | illumina HiSeq |
| 10625_4#85 | ERR363321 | SVH85 | NA | NA | NA | Dublin | NA | Bryant et al., (2016) Manuscript in preperation | illumina HiSeq |
| 10625_4#84 | ERR363320 | RVI84 | NA | NA | NA | UK | 2012 | Bryant et al., (2016) Manuscript in preperation | illumina HiSeq |
| 10625_4#8 | ERR363244 | WRCM8 | NA | NA | NA | UK | NA | Bryant et al., (2016) Manuscript in preperation | illumina HiSeq |
| 10625_4#6 | ERR363242 | WRCM6 | NA | NA | NA | UK | NA | Bryant et al., (2016) Manuscript in preperation | illumina HiSeq |
| 10625_4#51 | ERR363287 | RHS51 | NA | NA | NA | UK | 2011 | Bryant et al., (2016) Manuscript in preperation | illumina HiSeq |
| 10625_4#5 | ERR363241 | WRCM5 | NA | NA | NA | UK | NA | Bryant et al., (2016) Manuscript in preperation | illumina HiSeq |
| 10625_4#43 | ERR363279 | RHS43 | NA | NA | NA | UK | 2011 | Bryant et al., (2016) Manuscript in preperation | illumina HiSeq |
| 10625_4#4 | ERR363240 | WRCM4 | NA | NA | NA | UK | NA | Bryant et al., (2016) Manuscript in preperation | illumina HiSeq |
| 10625_4#37 | ERR363273 | RHS37 | NA | NA | NA | UK | 2011 | Bryant et al., (2016) Manuscript in preperation | illumina HiSeq |
| 10625_4#33 | ERR363269 | RHS33 | NA | NA | NA | UK | 2011 | Bryant et al., (2016) Manuscript in preperation | illumina HiSeq |

| | | | | | | | | | |
|---|---|---|---|---|---|---|---|---|---|
| 10625_4#32 | ERR363268 | RVI32 | NA | NA | NA | UK | 2012 | Bryant et al., (2016) Manuscript in preperation | illumina HiSeq |
| 10625_4#31 | ERR363267 | RVI31 | NA | NA | NA | UK | 2012 | Bryant et al., (2016) Manuscript in preperation | illumina HiSeq |
| 10625_4#30 | ERR363266 | RVI30 | NA | NA | NA | UK | 2012 | Bryant et al., (2016) Manuscript in preperation | illumina HiSeq |
| 10625_4#28 | ERR363264 | RVI28 | NA | NA | NA | UK | 2011 | Bryant et al., (2016) Manuscript in preperation | illumina HiSeq |
| 10625_4#26 | ERR363262 | RVI26 | NA | NA | NA | UK | 2011 | Bryant et al., (2016) Manuscript in preperation | illumina HiSeq |
| 10625_4#25 | ERR363261 | RVI25 | NA | NA | NA | UK | 2011 | Bryant et al., (2016) Manuscript in preperation | illumina HiSeq |
| 10625_4#24 | ERR363260 | RVI24 | NA | NA | NA | UK | 2011 | Bryant et al., (2016) Manuscript in preperation | illumina HiSeq |
| 10625_4#23 | ERR363259 | RVI23 | NA | NA | NA | UK | 2011 | Bryant et al., (2016) Manuscript in preperation | illumina HiSeq |
| 10625_4#22 | ERR363258 | RVI22 | NA | NA | NA | UK | 2011 | Bryant et al., (2016) Manuscript in preperation | illumina HiSeq |
| 10625_4#21 | ERR363257 | RVI21 | NA | NA | NA | UK | 2011 | Bryant et al., (2016) Manuscript in preperation | illumina HiSeq |
| 10625_4#20 | ERR363256 | RVI20 | NA | NA | NA | UK | 2011 | Bryant et al., (2016) Manuscript in preperation | illumina HiSeq |
| 10625_4#19 | ERR363255 | RVI19 | NA | NA | NA | UK | 2011 | Bryant et al., (2016) Manuscript in preperation | illumina HiSeq |
| 10625_4#18 | ERR363254 | WRCM18 | NA | NA | NA | UK | NA | Bryant et al., (2016) Manuscript in preperation | illumina HiSeq |
| 10625_4#17 | ERR363253 | WRCM17 | NA | NA | NA | UK | NA | Bryant et al., (2016) Manuscript in preperation | illumina HiSeq |
| 10625_4#16 | ERR363252 | WRCM16 | NA | NA | NA | UK | NA | Bryant et al., (2016) Manuscript in preperation | illumina HiSeq |
| 10625_4#14 | ERR363250 | WRCM14 | NA | NA | NA | UK | NA | Bryant et al., (2016) Manuscript in preperation | illumina HiSeq |
| 10625_4#13 | ERR363249 | WRCM13 | NA | NA | NA | UK | NA | Bryant et al., (2016) Manuscript in preperation | illumina HiSeq |
| 10625_4#11 | ERR363247 | WRCM11 | NA | NA | NA | UK | NA | Bryant et al., (2016) Manuscript in preperation | illumina HiSeq |
| 10625_4#10 | ERR363246 | WRCM10 | NA | NA | NA | UK | NA | Bryant et al., (2016) Manuscript in preperation | illumina HiSeq |
| 10465_1#75 | ERR349294 | UNC722 | NA | NA | NA | US | NA | Bryant et al., (2016) Manuscript in preperation | illumina HiSeq |
| 10465_1#74 | ERR349293 | UNC721 | NA | NA | NA | US | NA | Bryant et al., (2016) Manuscript in preperation | illumina HiSeq |
| 10465_1#71 | ERR349290 | UNC703 | NA | NA | NA | US | NA | Bryant et al., (2016) Manuscript in preperation | illumina HiSeq |

| | | | | | | | | | |
|---|---|---|---|---|---|---|---|---|---|
| 10465_1#70 | ERR349289 | UNC699 | NA | NA | NA | US | NA | Bryant et al., (2016) Manuscript in preperation | illumina HiSeq |
| 10465_1#68 | ERR349287 | UNC692 | NA | NA | NA | US | NA | Bryant et al., (2016) Manuscript in preperation | illumina HiSeq |
| 10465_1#61 | ERR349280 | UNC662 | NA | NA | NA | US | NA | Bryant et al., (2016) Manuscript in preperation | illumina HiSeq |
| 10465_1#60 | ERR349279 | UNC659 | NA | NA | NA | US | NA | Bryant et al., (2016) Manuscript in preperation | illumina HiSeq |
| 10465_1#59 | ERR349278 | UNC656 | NA | NA | NA | US | NA | Bryant et al., (2016) Manuscript in preperation | illumina HiSeq |
| 10465_1#58 | ERR349277 | UNC653 | NA | NA | NA | US | NA | Bryant et al., (2016) Manuscript in preperation | illumina HiSeq |
| 10465_1#57 | ERR349276 | UNC644 | NA | NA | NA | US | NA | Bryant et al., (2016) Manuscript in preperation | illumina HiSeq |
| 10465_1#55 | ERR349274 | UNC617 | NA | NA | NA | US | NA | Bryant et al., (2016) Manuscript in preperation | illumina HiSeq |
| 10465_1#54 | ERR349273 | UNC610 | NA | NA | NA | US | NA | Bryant et al., (2016) Manuscript in preperation | illumina HiSeq |
| 10465_1#53 | ERR349272 | UNC609 | NA | NA | NA | US | NA | Bryant et al., (2016) Manuscript in preperation | illumina HiSeq |
| 10465_1#51 | ERR349270 | UNC593 | NA | NA | NA | US | NA | Bryant et al., (2016) Manuscript in preperation | illumina HiSeq |
| 10465_1#44 | ERR349263 | DEN525 | NA | NA | NA | Denmark | NA | Bryant et al., (2016) Manuscript in preperation | illumina HiSeq |
| 10396_8#8 | ERR351976 | SMRL404 | NA | NA | NA | UK | 2010 | Bryant et al., (2016) Manuscript in preperation | illumina HiSeq |
| 10396_8#5 | ERR351973 | SMRL396 | NA | NA | NA | UK | 2010 | Bryant et al., (2016) Manuscript in preperation | illumina HiSeq |
| 10396_8#11 | ERR351979 | SMRL420 | NA | NA | NA | UK | 2010 | Bryant et al., (2016) Manuscript in preperation | illumina HiSeq |
| 10396_8#10 | ERR351978 | SMRL419 | NA | NA | NA | UK | NA | Bryant et al., (2016) Manuscript in preperation | illumina HiSeq |
| 10250_1#76 | ERR343241 | SMRL416 | NA | NA | NA | UK | 2010 | Bryant et al., (2016) Manuscript in preperation | illumina HiSeq |
| 10250_1#73 | ERR343238 | SMRL413 | NA | NA | NA | UK | 2010 | Bryant et al., (2016) Manuscript in preperation | illumina HiSeq |
| 10250_1#7 | ERR343181 | SMRL347 | NA | NA | NA | UK | 2008 | Bryant et al., (2016) Manuscript in preperation | illumina HiSeq |
| 10250_1#69 | ERR343235 | SMRL409 | NA | NA | NA | UK | 2010 | Bryant et al., (2016) Manuscript in preperation | illumina HiSeq |
| 10250_1#68 | ERR343234 | SMRL408 | NA | NA | NA | UK | NA | Bryant et al., (2016) Manuscript in preperation | illumina HiSeq |
| 10250_1#62 | ERR343230 | SMRL402 | NA | NA | NA | UK | 2010 | Bryant et al., (2016) Manuscript in preperation | illumina HiSeq |

| | | | | | | | | | |
|---|---|---|---|---|---|---|---|---|---|
| 10250_1#6 | ERR343180 | SMRL346 | NA | NA | NA | UK | 2008 | Bryant et al., (2016) Manuscript in preperation | illumina HiSeq |
| 10250_1#49 | ERR343220 | SMRL389 | NA | NA | NA | UK | 2009 | Bryant et al., (2016) Manuscript in preperation | illumina HiSeq |
| 10250_1#44 | ERR343217 | SMRL384 | NA | NA | NA | UK | 2009 | Bryant et al., (2016) Manuscript in preperation | illumina HiSeq |
| 10250_1#37 | ERR343211 | SMRL377 | NA | NA | NA | UK | 2009 | Bryant et al., (2016) Manuscript in preperation | illumina HiSeq |
| 10250_1#33 | ERR343207 | SMRL373 | NA | NA | NA | UK | 2009 | Bryant et al., (2016) Manuscript in preperation | illumina HiSeq |
| 10250_1#30 | ERR343204 | SMRL370 | NA | NA | NA | UK | 2009 | Bryant et al., (2016) Manuscript in preperation | illumina HiSeq |
| 10250_1#3 | ERR343177 | SMRL343 | NA | NA | NA | UK | 2008 | Bryant et al., (2016) Manuscript in preperation | illumina HiSeq |
| 10250_1#26 | ERR343200 | SMRL366 | NA | NA | NA | UK | 2008 | Bryant et al., (2016) Manuscript in preperation | illumina HiSeq |
| 10250_1#20 | ERR343194 | SMRL360 | NA | NA | NA | UK | 2008 | Bryant et al., (2016) Manuscript in preperation | illumina HiSeq |
| 10250_1#16 | ERR343190 | SMRL356 | NA | NA | NA | UK | 2008 | Bryant et al., (2016) Manuscript in preperation | illumina HiSeq |
| 10250_1#15 | ERR343189 | SMRL355 | NA | NA | NA | UK | 2008 | Bryant et al., (2016) Manuscript in preperation | illumina HiSeq |
| 10250_1#13 | ERR343187 | SMRL353 | NA | NA | NA | UK | 2008 | Bryant et al., (2016) Manuscript in preperation | illumina HiSeq |
| 10250_1#10 | ERR343184 | SMRL350 | NA | NA | NA | UK | 2008 | Bryant et al., (2016) Manuscript in preperation | illumina HiSeq |
| 10250_1#1 | ERR343175 | SMRL341 | NA | NA | NA | UK | 2008 | Bryant et al., (2016) Manuscript in preperation | illumina HiSeq |
| 10208_3#84 | ERR340574 | AHL509 | NA | NA | NA | UK | 2012 | Bryant et al., (2016) Manuscript in preperation | illumina HiSeq |
| 10208_3#83 | ERR340573 | AHL508 | NA | NA | NA | UK | 2013 | Bryant et al., (2016) Manuscript in preperation | illumina HiSeq |
| 10208_3#82 | ERR340572 | AHL507 | NA | NA | NA | UK | 2012 | Bryant et al., (2016) Manuscript in preperation | illumina HiSeq |
| 10208_3#81 | ERR340571 | AHL506 | NA | NA | NA | UK | 2012 | Bryant et al., (2016) Manuscript in preperation | illumina HiSeq |
| 10208_3#8 | ERR340499 | SMRL433 | NA | NA | NA | UK | 2011 | Bryant et al., (2016) Manuscript in preperation | illumina HiSeq |
| 10208_3#79 | ERR340569 | LTH504 | NA | NA | NA | UK | 2012 | Bryant et al., (2016) Manuscript in preperation | illumina HiSeq |
| 10208_3#78 | ERR340568 | LTH503 | NA | NA | NA | UK | 2012 | Bryant et al., (2016) Manuscript in preperation | illumina HiSeq |
| 10208_3#77 | ERR340567 | LTH502 | NA | NA | NA | UK | 2012 | Bryant et al., (2016) Manuscript in preperation | illumina HiSeq |

| | | | | | | | | | |
|---|---|---|---|---|---|---|---|---|---|
| 10208_3#76 | ERR340566 | LTH501 | NA | NA | NA | UK | 2012 | Bryant et al., (2016) Manuscript in preperation | illumina HiSeq |
| 10208_3#75 | ERR340565 | LTH500 | NA | NA | NA | UK | 2012 | Bryant et al., (2016) Manuscript in preperation | illumina HiSeq |
| 10208_3#74 | ERR340564 | LTH499 | NA | NA | NA | UK | 2012 | Bryant et al., (2016) Manuscript in preperation | illumina HiSeq |
| 10208_3#73 | ERR340563 | LTH498 | NA | NA | NA | UK | 2012 | Bryant et al., (2016) Manuscript in preperation | illumina HiSeq |
| 10208_3#71 | ERR340562 | LTH496 | NA | NA | NA | UK | 2012 | Bryant et al., (2016) Manuscript in preperation | illumina HiSeq |
| 10208_3#70 | ERR340561 | LTH495 | NA | NA | NA | UK | 2012 | Bryant et al., (2016) Manuscript in preperation | illumina HiSeq |
| 10208_3#69 | ERR340560 | LTH494 | NA | NA | NA | UK | 2012 | Bryant et al., (2016) Manuscript in preperation | illumina HiSeq |
| 10208_3#68 | ERR340559 | LTH493 | NA | NA | NA | UK | 2012 | Bryant et al., (2016) Manuscript in preperation | illumina HiSeq |
| 10208_3#67 | ERR340558 | LTH492 | NA | NA | NA | UK | 2012 | Bryant et al., (2016) Manuscript in preperation | illumina HiSeq |
| 10208_3#66 | ERR340557 | LTH491 | NA | NA | NA | UK | 2012 | Bryant et al., (2016) Manuscript in preperation | illumina HiSeq |
| 10208_3#65 | ERR340556 | RBL490 | NA | NA | NA | UK | NA | Bryant et al., (2016) Manuscript in preperation | illumina HiSeq |
| 10208_3#64 | ERR340555 | RBL489 | NA | NA | NA | UK | NA | Bryant et al., (2016) Manuscript in preperation | illumina HiSeq |
| 10208_3#63 | ERR340554 | RBL488 | NA | NA | NA | UK | NA | Bryant et al., (2016) Manuscript in preperation | illumina HiSeq |
| 10208_3#62 | ERR340553 | RBL487 | NA | NA | NA | UK | NA | Bryant et al., (2016) Manuscript in preperation | illumina HiSeq |
| 10208_3#61 | ERR340552 | RBL486 | NA | NA | NA | UK | NA | Bryant et al., (2016) Manuscript in preperation | illumina HiSeq |
| 10208_3#60 | ERR340551 | RBL485 | NA | NA | NA | UK | NA | Bryant et al., (2016) Manuscript in preperation | illumina HiSeq |
| 10208_3#6 | ERR340497 | SMRL431 | NA | NA | NA | UK | 2011 | Bryant et al., (2016) Manuscript in preperation | illumina HiSeq |
| 10208_3#59 | ERR340550 | RBL484 | NA | NA | NA | UK | NA | Bryant et al., (2016) Manuscript in preperation | illumina HiSeq |
| 10208_3#58 | ERR340549 | RBL483 | NA | NA | NA | UK | NA | Bryant et al., (2016) Manuscript in preperation | illumina HiSeq |
| 10208_3#57 | ERR340548 | RBL482 | NA | NA | NA | UK | NA | Bryant et al., (2016) Manuscript in preperation | illumina HiSeq |
| 10208_3#56 | ERR340547 | RBL481 | NA | NA | NA | UK | NA | Bryant et al., (2016) Manuscript in preperation | illumina HiSeq |
| 10208_3#55 | ERR340546 | RBL480 | NA | NA | NA | UK | NA | Bryant et al., (2016) Manuscript in preperation | illumina HiSeq |

| | | | | | | | | | |
|---|---|---|---|---|---|---|---|---|---|
| 10208_3#54 | ERR340545 | RBL479 | NA | NA | NA | UK | NA | Bryant et al., (2016) Manuscript in preperation | illumina HiSeq |
| 10208_3#53 | ERR340544 | RBL478 | NA | NA | NA | UK | NA | Bryant et al., (2016) Manuscript in preperation | illumina HiSeq |
| 10208_3#52 | ERR340543 | RBL477 | NA | NA | NA | UK | NA | Bryant et al., (2016) Manuscript in preperation | illumina HiSeq |
| 10208_3#51 | ERR340542 | RBL476 | NA | NA | NA | UK | NA | Bryant et al., (2016) Manuscript in preperation | illumina HiSeq |
| 10208_3#50 | ERR340541 | RBL475 | NA | NA | NA | UK | NA | Bryant et al., (2016) Manuscript in preperation | illumina HiSeq |
| 10208_3#5 | ERR340496 | SMRL430 | NA | NA | NA | UK | 2011 | Bryant et al., (2016) Manuscript in preperation | illumina HiSeq |
| 10208_3#49 | ERR340540 | RBL474 | NA | NA | NA | UK | NA | Bryant et al., (2016) Manuscript in preperation | illumina HiSeq |
| 10208_3#48 | ERR340539 | RBL473 | NA | NA | NA | UK | NA | Bryant et al., (2016) Manuscript in preperation | illumina HiSeq |
| 10208_3#47 | ERR340538 | RBL472 | NA | NA | NA | UK | NA | Bryant et al., (2016) Manuscript in preperation | illumina HiSeq |
| 10208_3#46 | ERR340537 | RBL471 | NA | NA | NA | UK | NA | Bryant et al., (2016) Manuscript in preperation | illumina HiSeq |
| 10208_3#45 | ERR340536 | RBL470 | NA | NA | NA | UK | NA | Bryant et al., (2016) Manuscript in preperation | illumina HiSeq |
| 10208_3#44 | ERR340535 | RVI469 | NA | NA | NA | UK | 2012 | Bryant et al., (2016) Manuscript in preperation | illumina HiSeq |
| 10208_3#42 | ERR340533 | SMRL467 | NA | NA | NA | UK | 2011 | Bryant et al., (2016) Manuscript in preperation | illumina HiSeq |
| 10208_3#40 | ERR340531 | SMRL465 | NA | NA | NA | UK | 2011 | Bryant et al., (2016) Manuscript in preperation | illumina HiSeq |
| 10208_3#39 | ERR340530 | SMRL464 | NA | NA | NA | UK | 2011 | Bryant et al., (2016) Manuscript in preperation | illumina HiSeq |
| 10208_3#36 | ERR340527 | SMRL461 | NA | NA | NA | UK | 2011 | Bryant et al., (2016) Manuscript in preperation | illumina HiSeq |
| 10208_3#35 | ERR340526 | SMRL460 | NA | NA | NA | UK | 2011 | Bryant et al., (2016) Manuscript in preperation | illumina HiSeq |
| 10208_3#32 | ERR340523 | SMRL457 | NA | NA | NA | UK | 2011 | Bryant et al., (2016) Manuscript in preperation | illumina HiSeq |
| 10208_3#31 | ERR340522 | SMRL456 | NA | NA | NA | UK | 2011 | Bryant et al., (2016) Manuscript in preperation | illumina HiSeq |
| 10208_3#30 | ERR340521 | SMRL455 | NA | NA | NA | UK | 2011 | Bryant et al., (2016) Manuscript in preperation | illumina HiSeq |
| 10208_3#3 | ERR340494 | SMRL428 | NA | NA | NA | UK | 2011 | Bryant et al., (2016) Manuscript in preperation | illumina HiSeq |
| 10208_3#29 | ERR340520 | SMRL454 | NA | NA | NA | UK | 2011 | Bryant et al., (2016) Manuscript in preperation | illumina HiSeq |

| | | | | | | | | | |
|---|---|---|---|---|---|---|---|---|---|
| 10208_3#27 | ERR340518 | SMRL452 | NA | NA | NA | UK | 2011 | Bryant et al., (2016) Manuscript in preperation | illumina HiSeq |
| 10208_3#26 | ERR340517 | SMRL451 | NA | NA | NA | UK | 2011 | Bryant et al., (2016) Manuscript in preperation | illumina HiSeq |
| 10208_3#25 | ERR340516 | SMRL450 | NA | NA | NA | UK | 2011 | Bryant et al., (2016) Manuscript in preperation | illumina HiSeq |
| 10208_3#21 | ERR340512 | SMRL446 | NA | NA | NA | UK | 2011 | Bryant et al., (2016) Manuscript in preperation | illumina HiSeq |
| 10208_3#20 | ERR340511 | SMRL445 | NA | NA | NA | UK | NA | Bryant et al., (2016) Manuscript in preperation | illumina HiSeq |
| 10208_3#2 | ERR340493 | SMRL427 | NA | NA | NA | UK | 2011 | Bryant et al., (2016) Manuscript in preperation | illumina HiSeq |
| 10208_3#19 | ERR340510 | SMRL444 | NA | NA | NA | UK | 2011 | Bryant et al., (2016) Manuscript in preperation | illumina HiSeq |
| 10208_3#16 | ERR340507 | SMRL441 | NA | NA | NA | UK | NA | Bryant et al., (2016) Manuscript in preperation | illumina HiSeq |
| 10208_3#15 | ERR340506 | SMRL440 | NA | NA | NA | UK | 2011 | Bryant et al., (2016) Manuscript in preperation | illumina HiSeq |
| 10071_6#80 | ERR337811 | SMRL165 | NA | NA | NA | UK | 2001 | Bryant et al., (2016) Manuscript in preperation | illumina HiSeq |
| 10071_6#72 | ERR337803 | SMRL157 | NA | NA | NA | UK | 2001 | Bryant et al., (2016) Manuscript in preperation | illumina HiSeq |
| 10060_7#2 | ERR330752 | WRCM2 | NA | NA | NA | UK | NA | Bryant et al., (2016) Manuscript in preperation | illumina HiSeq |

*For these isolates the raw reads for mapping were generated from the draft assembly. †This column consists of the identifiers used for these isolates in Bryant et al 2013. ‡These isolates have been previously sequenced. BRA-GO-06 is GO-06, BRA_RJ_08 is CRM0020 and BRA_PA_17 is INCQS 00594.

**Supplementary Table 2**: Annotation of Del_12078_1#71_01025_01041

The PROKKA, InterPro and GO-term annotations of the CDSs present within the globally circulating clone cluster 2 isolate, PAP1174, but lost in the Brazil lineage isolate, BRA_PA_42, starting at position 639781. (This is the start position of the deletion in BRA_PA_42 when the BRA_PA_42 assembly begins with CDS LgrD_9).

| Coding Sequence Locus Tag | Annotation (PROKKA) | Annotation (InterPro) | GO-term† |
|---|---|---|---|
| 12078_1#71_01025 | site-specific recombinase XerD,site-specific tyrosine recombinase XerC,Site-specific recombinase XerD,tyrosine recombinase XerC,Phage integrase family | Domain: Integrase, catalytic, Integrase, SAM-like, N terminal,DNA breaking-rejoining enzyme, catalytic core; integrase/recombinase N-terminal | F: DNA binding P: DNA recombination; P: DNA integration |
| 12078_1#71_01026 | Hypothetical protein | Domain: integrase-like catalytic domain | F: DNA binding P: DNA recombination; P: DNA integration |
| 12078_1#71_01027 | phage integrase family protein | Domain: DNA breaking-rejoining enzyme, catalytic core, integrase-like catalytic domain | F: DNA binding P: DNA recombination; P: DNA integration |
| 12078_1#71_01028 | Hypothetical protein | Domain: Homeodomain-like | F: DNA binding |
| 12078_1#71_01029 | Type III restriction enzyme, res subunit | Domain: Helicase/UvrB, N-terminal, Helicase superfamily, ATP-binding domain, P-loop containing nucleoside triphosphate hydrolase | F: DNA bidning F: ATP bidnding; F: hydrolase activity |
| 12078_1#71_01030 | Domain: Homeodomain-like | Cro/C1-type helix-turn-helix domain, Lambda repressor-like, DNA binding domain | F: sequence specific DNA binding |
| 12078_1#71_01031 | Hypothetical protein | no IPS | No GO-terms predicted |
| 12078_1#71_01032 | Hypothetical protein | no IPS | No GO-terms predicted |
| 12078_1#71_01033 | Predicted ATPase | Domain: ATPase, AAA-type core, P-loop containing nucleoside triphosphate hydorlases | F: ATP binding |
| 12078_1#71_01034 | amidohydrolase,Predicted metal-dependent hydrolase of the TIM-barrel fold,Amidohydrolase | Domain: Amidohydrolase-related, metal dependent hydorlase, amidohydrolase related | F: hydrolase activity |

| | | | |
|---|---|---|---|
| 12078_1#71_01035 | Hypothetical protein | no IPS | No GO-terms predicted |
| 12078_1#71_01036 | RNA polymerase sigma factor RpoS, TnsA endonuclease N terminal | Domain: RNA polymerase sigma-70, RNA polymerase sigma-70 region 4, winged helix-turn-helix DNA binding domain, RNA polymerase sigma factor, region 3/4, TnsA endonuclease, N-terminal | F: DNA binding; F: sigma factor activity, sequence specific DNA binding; P: regulation of transcription, DNA-templated; P: DNA-templated transcription, initiation |
| 12078_1#71_01037 | Hypothetical protein | no IPS | No GO-terms predicted |
| 12078_1#71_01038 | Hypothetical protein | Domain: bacterial toxin 33 | No GO-terms predicted |
| 12078_1#71_01039 | Hypothetical protein | DUF4279 | No GO-terms predicted |
| 12078_1#71_01040 | Putative liporotein LppU | no IPS | No GO-terms predicted |
| 12078_1#71_01041 | Hypothetical protein | no IPS | No GO-terms predicted |

†GO-terms: F = Function, P= Process; Annotation (InterPro): no IPS stands for "no Interpro scan result"

**Supplementary Table 3: Annotation of Del_12078_1#71_02620_02653**

The PROKKA, InterPro and GO-term annotations of the CDSs present within the globally circulating clone cluster 2 isolate, PAP1174, but lost in the Brazil lineage isolate, BRA_PA_42, starting at position 2076766 (This is the start position of the deletion in BRA_PA_42 when the BRA_PA_42 assembly begins with CDS LgrD_9).

| Coding Sequence Locus Tag | Annotation (PROKKA) | Annotation(InterPro) | GO terms† |
|---|---|---|---|
| 12078_1#71_02602 | Conserved hypothetical protein (carboxymuconolactone decarboxylase?),Carboxymuconolactone decarboxylase family | Domain: carboxymuconolactone decarboxylase-like, AhpD-like, Carboxymuconolactone decarboxylase-like | p: oxidation-reduction process; F: peroxiredoxin activity |
| 12078_1#71_02603 | Zn-dependent alcohol dehydrogenase,Sorbitol dehydrogenase,L-threonine 3-dehydrogenase,putative phosphonate catabolism associated alcohol dehydrogenase,Alcohol dehydrogenase GroES-like domain | Family: alcohol dehyrogenase superfamily, zinc-type, Domain: GroES-like, Alcohol dehyrogenase, C-terminal and N-terminal, NAD(P)-binding domains | F: zinc ion binding; F: oxidoreductase activity; p: oxidation reduction process |
| 12078_1#71_02604 | acetyl-CoA acetyltransferase,acetyl-CoA acetyltransferase | Domain: Thiolase like | F: ctalytic activity; P: metabolic process |
| 12078_1#71_02605 | TetR family transcriptional regulator,Bacterial regulatory proteins, tetR family | Domain: DNA-binding HTH tetR type, homeodomain-like, tetracycline transcriptional regulator, TetR-like, C-terminal. | F: DNA binding |
| 12078_1#71_02606 | Probable phenylacetic acid degradation-related protein,Putative esterase HI_1161,thioesterase,Uncharacterized protein, possibly involved in aromatic compounds catabolism,uncharacterized domain 1,Thioesterase superfamily | Domains: Phenylacetic acid degradation-related domain, thioseterase, HotDog | No GO-terms predicted |
| 12078_1#71_02607 | Putative hydrolase (alpha/beta fold),short chain dehydrogenase,Esterase/lipase,3-oxoadipate enol-lactonase,Alpha/beta hydrolase family | Domain: serine aminopeptidase, S33, alpha/beta hydrolase fold | No GO-terms predicted |
| 12078_1#71_02608 | Possible transcriptional regulator, TetR family,Bacterial regulatory proteins, tetR family | Domain: DNA-binding HTH tetR type, homeodomain-like, tetracycline transcriptional regulator, TetR-like, C-terminal. | F: DNA binding |

| | | | |
|---|---|---|---|
| 12078_1#71_02609 | Probable ferredoxin reductase,Putidaredoxin reductase,phenylpropionate dioxygenase ferredoxin reductase subunit,Uncharacterized conserved protein,nitrite reductase [NAD(P)H], large subunit,Pyridine nucleotide-disulphide oxidoreductase | Domain: Pyridine nucleotide-disulphide oxidoreductase, dimersiation, FAD/NAD-linked reductase, dimerisation, FAD/NAD(P)-binding, Reductase C-terminal | F: oxidoreductase activity; F: flavin adenine dinucleotide binding; P: cell redox homeostatis; P: oxidation-reduction process |
| 12078_1#71_02610 | putative Linalool 8-monooxygenase,Linalool 8-monooxygenase,Uncharacterized protein conserved in bacteria,Cytochrome P450 | Family: cytochrome P450, B-class | F: iron ion binding; F: oscidoreductase activity, acting on paired condonrs, with incorporation or reduction of molecular oxygen; P: oxidation-reduction process; F: heme binding |
| 12078_1#71_02611 | Probable ferredoxin,Ferredoxin VI,Na( )- translocating NADH-quiNo GO-terms predicted reductase subunit F,ferredoxin, 2Fe-2S type, ISC system,2Fe-2S iron-sulfur cluster binding domain | Domains: 2Fe-2S ferredoxin-type iron-sulfur binding, beta-grasp | P: obsolete electron transport, F: electron carrier activity F: iron-sulfur cluster binding |
| 12078_1#71_02612 | Putative HTH-type transcriptional regulator AraC,Colonization factor antigen I subunit D,DNA-binding transcriptional regulator GadX,Adenosine deaminase,YSIRK- targeted surface antigen transcriptional regulator,Helix- turn-helix domain | Domains: Homeodomain-like, AraC-type HTH domain, N-terminal; Family: transcription regulation HTH, AraC-type | C: transcription factor complex; F: transcription factor activity, sequence specific DNA binding; P: regulation of transcription |
| 12078_1#71_02613 | Conserved hypothetical protein (thiolase?),thiolase,Predicted nucleic-acid-binding protein containing a Zn-ribbon | Domains: thiolase-like | F: catalytic activity; P: metabolic process |
| 12078_1#71_02614 | putative nucleic-acid-binding protein containing a Zn-ribbon,Predicted nucleic-acid-binding protein containing a Zn-ribbon,DUF35 OB-fold domain | Domains: DUF35, OB-fold, C-terminal, Nucleic acid binding, OB-fold, rubredoxin-like zinc ribbon domain. | No GO-terms predicted |
| 12078_1#71_02615 | Luciferase-like,F420-dependent glucose-6- phosphate dehydrogenase,methylenetetrahydromethanopterin reductase,probable F420-dependent oxidoreductase, | Domains: luciferase like | F: oxidorecutaxe activity, acting on paired donors, with |

| | | | |
|---|---|---|---|
| | MSMEG_2256 family,Luciferase-like monooxygenase | | incorporation or reduction of molecular oxygen; P: oxidation-reduction process |
| 12078_1#71_02616 | acyl dehydratase,(3R)-hydroxyacyl-ACP dehydratase subunit HadC | Family: UPF0336; Domain: HotDog | No GO-terms predicted |
| 12078_1#71_02617 | MaoC-like dehydratase,(3R)-hydroxyacyl-ACP dehydratase subunit HadB,Enoyl reductase domain of yeast- type FAS1,MaoC like domain | Domains: MaoC_like domain, HotDog domain | No GO-terms predicted |
| 12078_1#71_02618 | acyl dehydratase | Domains: HotDog | No GO-terms predicted |
| 12078_1#71_02619 | acyl dehydratase | Domains: MaoC-like, HotDog | No GO-terms predicted |
| 12078_1#71_02620 | carnitinyl-CoA dehydratase,Carnitinyl-CoA dehydratase,enoyl-CoA hydratase,naphthoate synthase,Enoyl- CoA hydratase/isomerase family | Family: Crotonase Domains: Crotonase C-terminal, ClpP/crotonase-like domain | F: catalytic activity; P: metabolic process |
| 12078_1#71_02621 | TIGR03084 family protein,conserved hypothetical protein,Mycothiol maleylpyruvate isomerase N-terminal domain | Family:Conserved hypotheical protein CHP03083, actinobacteria-type Domains: tRNA wybutosine-synthesis, Mycothiol dependent maleylpyruvate isomerase, metal binding, DinB/YfiT-like putative metal-dependent hydrolase | F: metal ion binding |
| 12078_1#71_02622 | protein of unknown function (DUF1446),Protein of unknown function (DUF1446) | Family: DUF1446 | No GO-terms predicted |
| 12078_1#71_02623 | Putative transcriptional regulator, TetR family,HTH-type transcriptional repressor KstR2,DNA- binding transcriptional repressor AcrR,mycofactocin system transcriptional regulator,Bacterial regulatory proteins, tetR family | Domain: DNA-binding HTH tetR type, homeodomain-like, tetracycline transcriptional regulator, TetR-like, C-terminal. | F: DNA binding |
| 12078_1#71_02624 | acyl-CoA synthetase (AMP-forming)/AMP-acid ligase II,Long-chain-fatty-acid--CoA ligase,long-chain- fatty-acid--CoA ligase,Uncharacterized protein conserved in bacteria,O-succinylbenzoate-CoA ligase,AMP-binding enzyme | Domain: AMP-dependent synthetase/ligase, AMP-binding enzyme C-terminal | F: catalytic activity; P: metabolic process |
| 12078_1#71_02625 | Probable acyl-CoA dehydrogenase FadE,Acyl-CoA | Domains: Acyl-CoA | F: acyl-CoA |

| | | | |
|---|---|---|---|
| | dehydrogenase fadE12,putative acyl-CoA dehydrogenase,Rubredoxin,cyclohexanecarboxyl-CoA dehydrogenase,Acyl-CoA dehydrogenase, C-terminal domain | oxidase/dehydrogenase, central domain, C-terminal domain, N-terminal and middle domain. | dehydrogenase activity; F: flavidn adenine dinucleotide binding; P: oxidation reduction process |
| 12078_1#71_02626 | Probable acetyl/propionyl carboxylase alpha subunit AccA2,Acetyl-/propionyl-coenzyme A carboxylase alpha chain,pyruvate carboxylase,Acetyl/propionyl-CoA carboxylase, alpha subunit,acetyl-CoA carboxylase, biotin carboxylase subunit,Carbamoyl-phosphate synthase L chain, ATP binding domain | Domains: Biotin/lipoyl attachment, Carbamoyl-phosphate synthetase large subunit-like ATP-binding domain, Biotin carboxylase like N-terminal/C-terminal domain, single hyrid motif, Rudiment single hybrid motif, ATP-grasp fold, Biotin carboxylation domain, ATP-grasp fold | F: ATP binding; F: metal ion binding; F: biotin carboxylase activity |
| 12078_1#71_02627 | carboxyl transferase,Methylmalonyl-CoA carboxyltransferase 12S subunit,acetyl-CoA carboxylase carboxyltransferase subunit alpha,Acetyl-CoA carboxylase, carboxyltransferase component (subunits alpha and beta),methylmalonyl-CoA decarboxylase alpha subunit,Carboxyl transferase domain | Domains: Carboxyl transferase, Acetyl-coenzyme A carboxyltransferase, C-terminal and N-termainl, clpP/crotonase-like | F: ligase activity |
| 12078_1#71_02628 | transposase,Transposase and inactivated derivatives | Domains: winged helix-turn-helix DNA binding domain | No GO-terms predicted |
| 12078_1#71_02629 | transposase,Transposase and inactivated derivatives | Domains: Integrase, catalytic core, Ribonuclease H-like domain | F: nucleic acid binding; P: DNA integration |
| 12078_1#71_02630 | protein DnaA,IstB-like ATP binding protein | Family: DNA replication protein DnaC/insertion sequence putative ATP-binding protein; Domains: IstB-like ATP-binding, AAA+ ATPase domain | F: ATP binding |
| 12078_1#71_02631 | transcriptional regulator,Bacterial regulatory proteins, tetR family | Domain: DNA-binding HTH tetR type, homeodomain-like, tetracycline transcriptional regulator, TetR-like, C-terminal. | F: DNA binding |
| 12078_1#71_02632 | putative metal-dependent hydrolase,Predicted metal-dependent hydrolase | Family: UCP07580 | No GO-terms predicted |

| | | | |
|---|---|---|---|
| 12078_1#71_02633 | TetR family transcriptional regulator,Bacterial regulatory proteins, tetR family | Domain: DNA-binding HTH tetR type, homeodomain-like, tetracycline transcriptional regulator, TetR-like, C-terminal. | F: DNA binding |
| 12078_1#71_02634 | Probable short-chain dehydrogenase/reductase,Diacetyl reductase [(S)-acetoin forming],short chain dehydrogenase,Uncharacterized conserved protein,3-hydroxybutyrate dehydrogenase,short chain dehydrogenase | Family: Short-chain, dehydrogenase/reductase SDR; Domain: NAD(P)-binding domain | No GO-terms predicted |
| 12078_1#71_02635 | reductase | Domain: Ferritin-like superfamily | No GO-terms predicted |
| 12078_1#71_02636 | putative flavoprotein involved in K transport,Phenylacetone monooxygenase,Uncharacterized protein conserved in bacteria,flavin-dependent oxidoreductase, MSMEG_0569 family,Flavin-binding monooxygenase-like | Domain: FAD/NAD(P) -binding domain | F: oxidoreductase activity P: oxidation-reduction process |
| 12078_1#71_02637 | putative flavoprotein involved in K transport,4-hydroxyacetopheNo GO-terms predicted monooxygenase,Uncharacterized protein conserved in bacteria,flavin-dependent oxidoreductase, MSMEG_0569 family,Flavin-binding monooxygenase-like | Domain: FAD/NAD(P) -binding domain | F: oxidoreductase activity P: oxidation-reduction process |
| 12078_1#71_02638 | Putative esterase/acetyl hydrolase,Monoterpene epsilon-lactone hydrolase,acetyl esterase,alpha/beta hydrolase fold | Domains: Alpha/beta hydrolase fold-3 | P: metabloic process; F: hydrolase activity |
| 12078_1#71_02639 | monooxygenase,4-hydroxyacetopheNo GO-terms predicted monooxygenase,dihydropyrimidine dehydrogenase subunit A,Protoporphyrinogen oxidase,flavin-dependent oxidoreductase, MSMEG_0569 family,Flavin-binding monooxygenase-like | Domains: FAD/NAD(P)-binding domain | F: oxidoreductase activity P: oxidation-reduction process |
| 12078_1#71_02640 | Probable cytochrome P450,Putative cytochrome P450 120,Cytochrome P450 | Family: Cytochrome P450, E-class, group IV | F: iron ion binding, F: oxidoreductase activity, acting on paired donors, with incorporation or reduction of molecular oxygen, F: mono-oxygenase activity, F:heme binding, P: oxidation-reduction process |

| | | | |
|---|---|---|---|
| 12078_1#71_02641 | Conserved hypothetical protein (transglutaminase?),Transglutaminase-like superfamily | Domains: Transglutaminase-like | No GO-terms predicted |
| 12078_1#71_02642 | acyl-CoA synthetase (AMP-forming)/AMP-acid ligase II,Short-chain-fatty-acid--CoA ligase,long-chain- fatty-acid--CoA ligase,Uncharacterized protein conserved in bacteria,O-succinylbenzoate-CoA ligase,AMP-binding enzyme | Domains: AMP-dependent synthetase/ligase, AMP-binding enzyme C-terminal domain | F: catalytic activity, P: metabolic process |
| 12078_1#71_02643 | Probable acyl CoA dehydrogenase,Acyl-CoA dehydrogenase, short-chain specific,putative acyl-CoA dehydrogenase,Rubredoxin,cyclohexanecarboxyl-CoA dehydrogenase,Acyl-CoA dehydrogenase, C-terminal domain | Domains: Acyl-CoA oxidase/dehyrogenase, central domain, N terminal and Middle Domain | F: acyl-CoA dehydrogenase activity; F: flavidn adenine dinucleotide binding; P: oxidation reduction process |
| 12078_1#71_02644 | hypothetical protein | Domain: Butirosin biosynthesis protein H, N-terminal, DUF4872 | No GO-terms predicted |
| 12078_1#71_02645 | putative sterol carrier protein | Domain: SCP2 sterol binding domain | No GO-terms predicted |
| 12078_1#71_02646 | Probable zinc-type alcohol dehydrogenase AdhD,S-(hydroxymethyl)glutathione dehydrogenase,putative alcohol dehydrogenase,Branched-chain amino acid aminotransferase/4- amino-4-deoxychorismate lyase,NDMA-dependent alcohol dehydrogenase, Rxyl_3153 family,Alcohol dehydrogenase GroES-like domain | Family: alcohol dehyrogenase superfamily, zinc-type, Domain:polyketide synthase enoylreductase doamin, Alcohol dehyrogenase, C-terminal and N-terminal, NAD(P)-binding domains, (no IPR) GroES-like | F: zinc ion binding F: oxidoreductase activity; P: oxidation-reduction process |
| 12078_1#71_02647 | Probable acyl CoA dehydrogenase,Acyl-CoA dehydrogenase, short-chain specific,putative acyl-CoA dehydrogenase,Rubredoxin,cyclohexanecarboxyl-CoA dehydrogenase,Acyl-CoA dehydrogenase, C-terminal domain | Domains: Acyl-CoA oxidase/dehyrogenase, central domain, C-terminal, N-terminal and Middle domain | F: acyl-CoA dehydrogenase activity; F: flavin adenine dinucleotide binding; P: oxidation-reduction process |
| 12078_1#71_02648 | amidase,Creatinine amidohydrolase,creatinine amidohydrolase family protein, mycofactocin system,Creatinine amidohydrolase | Family: Creatininase/formamide hydrolase; Domain: Creatininase-like | No GO-terms predicted |
| 12078_1#71_02649 | hypothetical protein | Domains: lambda repressor-like, DNA-binding | F: DNA binding |
| 12078_1#71_02650 | regulatory protein | no IPS | No GO-terms predicted |

| | | | |
|---|---|---|---|
| 12078_1#71_02651 | DNA segregation ATPase FtsK,DNA translocase FtsK,DNA translocase FtsK,DNA segregation ATPase FtsK/SpoIIIE and related proteins,type VII secretion protein EccCb,FtsK/SpoIIIE family | Domains: FtsK, AAA+ ATPase, P-loop containing nucleoside triphosphate | F: DNA binding; F: ATP binding |
| 12078_1#71_02652 | putative plasmid replication initiator protein | no IPS | No GO-terms predicted |
| 12078_1#71_02653 | hypothetical protein | no IPS | No GO-terms predicted |

†GO-terms: F = Function, P= Process; Annotation (InterPro): no IPS stands for “no Interpro scan result”

**Supplementary table 4:** Annotations of the coding sequences identified on pMAB02

| Coding Sequences | Annotation (PROKKA) | Annotation (Interpro) | Annotation (Pfam) |
|---|---|---|---|
| 16933_5#1_04630 (YAFL) | Invasion associated secreted endopeptidase | Endopeptidase, NLPC/P60 domain | Endopeptidase |
| 16933_5#1_04631 | Hypothetical protein | no IPS | no pfam |
| 16933_5#1_04632 | Hypothetical protein | ESX-1 secretion associated protein EspC | excrete virulence factor t7ss |
| 16933_5#1_04633 | Possible PPE protein | PPE | PPE |
| 16933_5#1_04634 | Hypothetical protein | no IPS | no pfam |
| 16933_5#1_04635 | Hypothetical protein | no IPS | no pfam |
| 16933_5#1_04636 | Hypothetical protein | no IPS | no pfam |
| 16933_5#1_04637 | Hypothetical protein | no IPS | no pfam |
| 16933_5#1_04638 | Hypothetical protein | no IPS | no pfam |
| 16933_5#1_04639 | Hypothetical protein | no IPS | no pfam |
| 16933_5#1_04640 | Conjugative transposon protein TcpC | Conjugative transposon protein TcpC | Conjugative transposon protein TcpC |
| 16933_5#1_04641 | Hypothetical protein | no IPS | no pfam |
| 16933_5#1_04642 | Hypothetical protein | no IPS | no pfam |
| 16933_5#1_04643 | Type IV secretory pathway, VirB4 components, AAA-like domain | Unitegrated signature (PF12846) | AAA-like domain |
| 16933_5#1_04644 | Hypothetical protein | no IPS | no pfam |
| 16933_5#1_04645 | Hypothetical protein | no IPS | no pfam |
| 16933_5#1_04646 | Hypothetical protein | no IPS | no pfam |
| 16933_5#1_04647 | Hypothetical protein | no IPS | no pfam |
| 16933_5#1_04648 | Hypothetical protein | no IPS | no pfam |
| 16933_5#1_04649 | Hypothetical protein | no IPS | no pfam |
| 16933_5#1_04650 | Hypothetical protein | no IPS | no pfam |
| 16933_5#1_04651 | Hypothetical protein | no IPS | no pfam |
| 16933_5#1_04652 | Hypothetical protein | no IPS | no pfam |
| 16933_5#1_04653 | Hypothetical protein | no IPS | no pfam |
| 16933_5#1_04654 | Hypothetical protein | no IPS | no pfam |
| 16933_5#1_04655 | Hypothetical protein | no IPS | no pfam |
| 16933_5#1_04656 | Hypothetical protein | no IPS | no pfam |
| 16933_5#1_04657 | Hypothetical protein | no IPS | no pfam |
| 16933_5#1_04658 | Hypothetical protein | no IPS | no pfam |

| | | | |
|---|---|---|---|
| 16933_5#1_04659 | Hypothetical protein | no IPS | no pfam |
| 16933_5#1_04660 | Hypothetical protein | no IPS | no pfam |
| 16933_5#1_04661 | Hypothetical protein | no IPS | no pfam |
| 16933_5#1_04662 | PilT domain-containing protein,PIN domain | PIN domain-like | PIN domain |
| 16933_5#1_04663 | Hypothetical protein | no IPS | no pfam |
| 16933_5#1_04664 | Hypothetical protein | no IPS | no pfam |
| 16933_5#1_04665 | Hypothetical protein | no IPS | no pfam |
| 16933_5#1_04666 | Hypothetical protein | no IPS | no pfam |
| 16933_5#1_04667 | putative transcriptional regulator, y4mf | Cro/C1-type h-t-h domain; Lambda repressor-like, DNA-binding domain | H-t-H domain |
| 16933_5#1_04668 (EspR_6) | espR_6 ESX-1 secretion-associated regulator EspR | Cro/C1-type h-t-h domain; Lambda repressor-like, DNA-binding domain | H-t-H domain |
| 16933_5#1_04669 | Putative regulator component | no IPS | no pfam |
| 16933_5#1_04670 | Hypothetical protein | no IPS | no pfam |
| 16933_5#1_04671 | Cytochrome p450 | Cytochrome P450, E-class, group 1 | Cytochrome p450 |
| 16933_5#1_04672 | Hypothetical protein | no IPS | no pfam |
| 16933_5#1_04673 | Hypothetical protein | no IPS | no pfam |
| 16933_5#1_04674 | Hypothetical protein | no IPS | no pfam |
| 16933_5#1_04675 | Putative methyl accepting chemotaxis sensory transducer | no IPS | no pfam |
| 16933_5#1_04676 (EccB1_2) | EccB5 | T7SS EccB | T7SS ESX-1, transport TM domain B |
| 16933_5#1_04677 (essC) | essC Putative FtsK/SpoIIIE family protein, type VII secretion protein EccCa | FtsK domain; P-loop containing nucleoside triphosphate hydrolase; | FtsK/SpoIIIE family |
| 16933_5#1_04678 | PE(?) | no IPS | no pfam |
| 16933_5#1_04679 | PPE protein | PPE family protein | PPE |
| 16933_5#1_04680 | WXG100 TypeVII secretion target | T7SS ESAT-6-like | WXG100 |
| 16933_5#1_04681 | Hypothetical protein | T7SS ESAT-6-like | no pfam |
| 16933_5#1_04682 | espG type VII | EspG family | ESX-1_EspG |
| 16933_5#1_04683 | Hypothetical protein | no IPS | no pfam |
| 16933_5#1_04684 | ATPase,Flp pilus assembly protein, ATPase CpaE,CobQ/CobB/MinD/ParA nucleotide binding domain | Domain: P-loop containing nucleoside triphosphate hydrolase; Domain: CobQ/CobB/MinD/ParA nucleotide binding domain | CobQ/cobB/MinD/ParA nucleotide binding domain |
| 16933_5#1_04685 | type VII secretion integral membrane protein EccD | no IPS | no pfam |
| 16933_5#1_04686 | peptidase S8 and S53, subtilisin, kexin, sedolisin,Subtilisin BL,Regulatory P domain of the | Family: Peptidase S8, subtilisin-related; T7SS peptidase S8A, | Peptidase_S8 |

| | | | |
|---|---|---|---|
| | subtilisin-like proprotein convertases and other proteases,type VII secretion-associated serine protease mycosin,Subtilase family, mycP (mycosin peptidase) | mycosin-1; Domain: Peptidase S8/S53 domain | |
| 16933_5#1_04687 | type VII secretion protein EccE | no IPS | no pfam |
| 16933_5#1_04688 (eccA1_2) | eccA1_2 type VII secretion AAA-ATPase EccA | Family:T7SS AAA-ATPase, EccA; Domain: AAA+ ATPase domain; P-loop containing nucleoside triphosphate hydrolase; ATPase, AAA-type, core | AAA family protein |
| 16933_5#1_04689 | Hypothetical protein | no IPS | no pfam |
| 16933_5#1_04690 | type IV secretory pathway VirD4 family protein,Type IV secretory pathway, VirD4 components,TraM recognition site of TraD and TraG | Domain: P-loop containing nucleoside triphosphate hydrolase; TraD/TraG, TraM recognition site | TraM recognition site of TraD and TraG |
| 16933_5#1_04691 | Hypothetical protein | no IPS | no pfam |
| 16933_5#1_04692 | Hypothetical protein | no IPS | no pfam |
| 16933_5#1_04693 | Hypothetical protein | no IPS | no pfam |
| 16933_5#1_04694 | Hypothetical protein | no IPS | no pfam |
| 16933_5#1_04695 (nucH) | nucH micrococcal nuclease-like nuclease,Thermonuclease precursor,Staphylococcal nuclease homologue | Domain: Staphylococcal nuclease (Snase-like), OB-fold; | Snase family, Staphyloccocal nuclease homologue |
| 16933_5#1_04696 | Hypothetical protein | no IPS | no pfam |
| 16933_5#1_04697 | Hypothetical protein | no IPS | no pfam |
| 16933_5#1_04698 (TraI) | TraI TrwC relaxase,Multifunctional conjugation protein TraI,Dtr system oriT relaxase,Ti-type conjugative transfer relaxase TraA,TrwC relaxase | Domain: P-loop containing nucleoside triphosphate hydrolase; TrwC relaxase | Family: TrwC, TrwC relaxase; AAA_30 protein |
| 16933_5#1_04699 | Hypothetical protein | no IPS | no pfam |
| 16933_5#1_04700 | Hypothetical protein | no IPS | no pfam |
| 16933_5#1_04701 | Hypothetical protein | no IPS | no pfam |
| 16933_5#1_04702 | Hypothetical protein | no IPS | no pfam |
| 16933_5#1_04703 | Hypothetical protein | no IPS | no pfam |
| 16933_5#1_04704 | Hypothetical protein | no IPS | no pfam |
| 16933_5#1_04705 | endopeptidase | Conserved Site: Twin-arginine translocation pathway, signal sequence; Domain: Peptidase S1, PA clan | no pfam |

| | | | |
|---|---|---|---|
| 16933_5#1_04706 | Hypothetical protein | no IPS | no pfam |
| 16933_5#1_04707 | Hypothetical protein | no IPS | no pfam |
| 16933_5#1_04708 | DNAB-like helicase N-terminal domain | no IPS | no pfam |
| 16933_5#1_04709 | Hypothetical protein | no IPS | no pfam |
| 16933_5#1_04710 (nrdH) | nrdH_2 glutaredoxin-like protein NrdH | Domain: Glutaredoxin; Thioredoxin-like fold; Family:Glutaredoxin-like protein NrdH | Glutaredoxin |
| 16933_5#1_04711 | Hypothetical protein | no IPS | no pfam |
| 16933_5#1_04712 | Hypothetical protein | no IPS | no pfam |
| 16933_5#1_04713 | Hypothetical protein | no IPS | no pfam |
| 16933_5#1_04714 | Hypothetical protein | no IPS | no pfam |
| 16933_5#1_04715 | Hypothetical protein | no IPS | no pfam |
| 16933_5#1_04716 | Hypothetical protein | no IPS | no pfam |
| 16933_5#1_04717 | Hypothetical protein | no IPS | no pfam |
| 16933_5#1_04718 | Hypothetical protein | no IPS | no pfam |
| 16933_5#1_04719 | phage/plasmid like protein | Family: DUF932; Phage/plasmid-like protein | Family: DUF932 |
| 16933_5#1_04720 | Hypothetical protein | no IPS | no pfam |
| 16933_5#1_04721 | hypothetical protein | no IPS | no pfam |
| 16933_5#1_04722 | Hypothetical protein | no IPS | no pfam |
| 16933_5#1_04723 | nuclease,ParB/RepB/Spo0J family partition protein,ParB-like nuclease domain | Domain: ParB/Sulfiredoxin | Family: ParBc (ParB-like nuclease domain) |
| 16933_5#1_04724 | Hypothetical protein | no IPS | no pfam |
| 16933_5#1_04725 | Hypothetical protein | no IPS | no pfam |
| 16933_5#1_04726 | Hypothetical protein | no IPS | no pfam |
| 16933_5#1_04727 (ssb_3) | Single stranded DNA binding protein | Domain: Nucleic acid binding, OB-fold; Family: Primosome PriB/single-strand DNA-binding | Family: single stranded DNA binding protein |
| 16933_5#1_04728 | Hypothetical protein | no IPS | no pfam |
| 16933_5#1_04729 | Biofilm formation regulator (?) | no IPS | no pfam |
| 16933_5#1_04730 | Hypothetical protein | no IPS | no pfam |
| 16933_5#1_04731 | Hypothetical protein | no IPS | no pfam |
| 16933_5#1_04732 | Hypothetical protein | no IPS | no pfam |
| 16933_5#1_04733 | Hypothetical protein | no IPS | no pfam |
| 16933_5#1_04734 | Hypothetical protein | no IPS | no pfam |
| 16933_5#1_04735 | Hypothetical protein | no IPS | no pfam |
| 16933_5#1_04736 | Hypothetical protein | no IPS | no pfam |

| | | | |
|---|---|---|---|
| 16933_5#1_04737 | Hypothetical protein | no IPS | no pfam |
| 16933_5#1_04738 (SOJ_4) | Plasmid partitioning protein repA/parA | Domain: P-loop containing nucleoside triphosphate hydrolase; AAA domain | AAA domain |
| 16933_5#1_04739 | Hypothetical protein | no IPS | no pfam |
| 16933_5#1_04740 (lppP) | Lipoprotein lppP | Family: LppP/LprE lipoprotein; uncharacterised protein family, liproprotein LppP | Family: Lipoprotein LppP/LprE |
| 16933_5#1_04741 | Helix-turn-helix (regulator?) | no IPS | no pfam |
| 16933_5#1_04742 | Hypothetical protein | no IPS | no pfam |
| 16933_5#1_04743 | Hypothetical protein | no IPS | no pfam |
| 16933_5#1_04744 | Hypothetical protein | no IPS | no pfam |
| 16933_5#1_04745 | Hypothetical protein | Domain: lambda repressor-like, DNA-binding domain | no pfam |
| 16933_5#1_04746 | putative transcriptional regulator | Domain: Cro/C1-type h-t-h domain; lambda repressor-like, DNA-binding domain | Family: h-t-h |
| 16933_5#1_04747 | Hypothetical protein | no IPS | no pfam |
| 16933_5#1_04748 | Hypothetical protein | no IPS | no pfam |
| 16933_5#1_04749 | Hypothetical protein | no IPS | no pfam |
| 16933_5#1_04750 | Hypothetical protein | no IPS | no pfam |
